# Controlled Optically Active Hierarchical Nanostructures of Side‐Chain Sequence‐Regulated Graphene Nanoribbons

**DOI:** 10.1002/advs.202523362

**Published:** 2026-02-03

**Authors:** Baiyang Chen, Kaiyuan Song, Li Yu, Lujia You, Yiming Wang, Zhaoguo Zhang, Yongfeng Zhou, Xinyuan Zhu, Ruijiao Dong

**Affiliations:** ^1^ Shanghai Center for Systems Biomedicine Key Laboratory of Systems Biomedicine (Ministry of Education) Shanghai Jiao Tong University Shanghai China; ^2^ School of Chemistry and Chemical Engineering Frontiers Science Centre for Transformative Molecules State Key Laboratory of Metal Matrix Composites Shanghai Jiao Tong University Shanghai China

**Keywords:** controlled side‐chain sequence, graphene nanoribbons, hierarchical nanostructures, tunable optical properties

## Abstract

Synthetic graphene nanoribbons (GNRs) have drawn significant attention due to their unique optical, electronic, and magnetic properties. Considerable efforts have been exerted in developing versatile synthetic methods to manipulate the architectures and properties of GNRs. However, these synthetic methodologies have still struggled to achieve a delicate control over the sequence and function of GNRs at the molecular level, thereby limiting their application potentials across a range of scientific and technological fields. Herein, we report a robust liquid‐phase bottom‐up synthesis strategy for the creation of sequence‐regulated functional GNRs, enabling precise control over the side‐chain sequence of GNRs. Using this approach, diversified sequence‐regulated GNRs have been produced, demonstrating readily regulated hierarchical nanostructures and optical properties, attributing to their sequence‐dependent molecular stacking mode. This liquid‐phase bottom‐up synthetic technology enabled the incorporation of the side‐chain structural and functional diversity into GNRs, further holding great promise for optoelectronic and biomedical applications.

## Graphical abstract

The restrained side‐chain accessibility and versatility of GNRs extremely impede their multifunctionality, solution processability, and numerous solution‐based applications. We report herein a robust liquid‐phase bottom‐up synthesis approach for high‐efficiency preparation of side‐chain sequence‐regulated functional GNRs. The structurally defined GNRs demonstrate a unique sequence‐encoded molecular stacking mode, enabling precise manipulation of hierarchical nanostructures and optical properties.

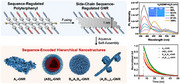

## 1 Introduction

In recent years, tremendous efforts have been made in manipulating the molecular architecture of graphene nanoribbons (GNRs) in order to upgrade their optical, electronic, and magnetic properties [1, 2, 3, 4, 5, 6, 7]. Unlike the top‐down methodology, the regulation over the backbone structures of GNRs, involving length, width, edge structure, and hybridization, have been achieved via bottom‐up chemical synthesis, expanding their application potential in miscellaneous scientific fields, such as electronics, energy, nanotechnology, and biotechnology [8, 9, 10, 11, 12, 13, 14, 15, 16]. For instance, bottom‐up on‐surface synthesis of GNRs with atomically precise zigzag edges was achieved through surface‐assisted polymerization followed by subsequent cyclodehydrogenation of predesigned molecular precursors [4, 14, 15, 16]. The bottom‐up liquid‐phase synthesis strategies have been developed to generate numerous highly ordered GNRs with large‐scale manufacturing capacity. Length‐controlled GNRs have been synthesized through accelerated liquid‐phase iterative synthesis, thereby establishing systematic correlations between chain length and optoelectronic properties. Moreover, a recently developed solution‐phase method allows the synthesis of backbone‐hybrid porphyrin‐fused GNRs, enabling the precise engineering of their electrical and magnetic properties [8, 9, 10]. However, the restrained side‐chain accessibility and versatility of GNRs extremely impede their multifunctionality, solution processability, and numerous solution‐based applications [17, 18, 19, 20, 21].

**FIGURE 1:**
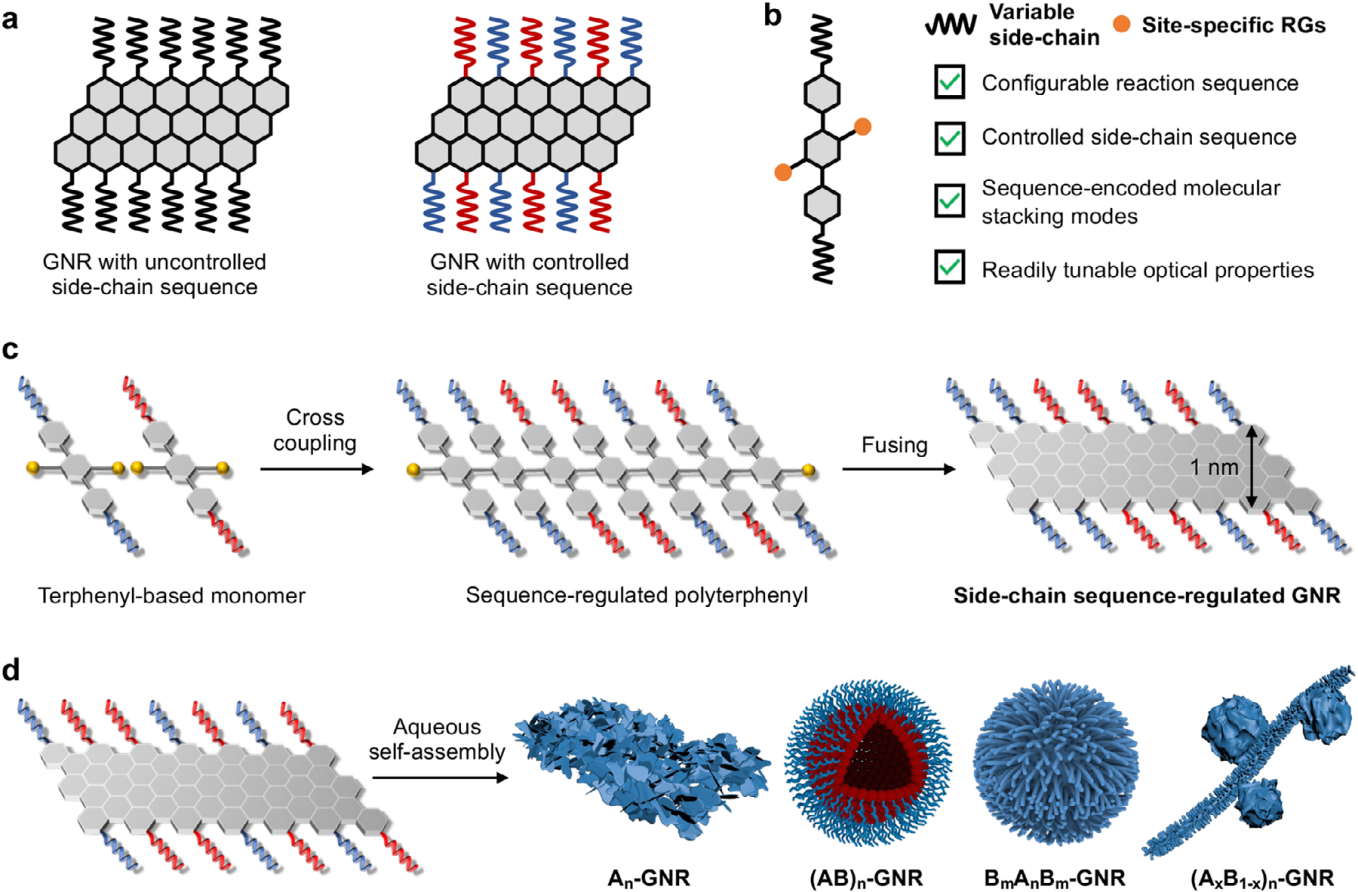
Design of hierarchical functional nanostructures from structurally defined GNRs with precisely regulated side‐chain sequence. (a) Previously reported GNRs functionalized with homogeneous side‐chains (left), and side‐chain sequence‐regulated functional GNRs in our work (right). (b) Delicately designed terphenyl‐based monomer with variable side‐chains and site‐specific reactive groups (RGs). (c) Illustration of the facile synthesis of GNRs with readily regulated side‐chain sequence. (d) Schematic controllable self‐assembly behaviors of side‐chain sequence‐regulated functional GNRs in solution.

A number of side‐chain functionalization strategies have been proposed to extend the application horizons of GNRs [18, 19]. Numerous studies have demonstrated that the self‐assembly behavior of polymers is strongly governed by their monomer sequences. For instance, Liu [22, 23, 24], Zhang [25, 26, 27], Du [28, 29], Feng [17, 18, 19, 30], Mai [20, 21, 31], and others have reported significant advances in the self‐assembly of sequence‐controlled polymers. The Feng group has demonstrated the bottom‐up solution synthesis of flexible polyethylene oxide (PEO)‐functionalized GNRs (GNR‐PEO) with excellent dispersibility in both common organic solvents and water. The remarkable solution processability of the GNR‐PEO enabled the fabrication of thin‐film‐based field‐effect transistors (FETs) with higher carrier mobility through directly drop‐casting the THF dispersions on Si/SiO_2_ substrates, far beyond those of previously reported GNR thin‐film based FETs [18]. Furthermore, Mai and coworkers have promoted the bottom‐up solution synthesis of GNRs grafting with diversified side‐chains, such as hydrophilic PEOs with variable molecular weights and benzyl ether‐type dendrons, thereby unlocking their potential for photothermal tumor therapy [19, 20, 21]. In nature, the defined sequence regulation in biomacromolecules typically administrated intramolecular and intermolecular interactions to generate versatile hierarchical nanostructures with specific functions, such as the double helix of DNA, as well as *α*‐helix and *β*‐sheet of proteins [32, 33]. Consequently, the sequence of bioactive monomers, such as amino acids and nucleotides, is widely recognized to exert a profound influence on the structure and function of living systems. With advances in sequence‐controlled synthetic chemistry, monomer sequence has emerged as a powerful handle for regulating polymer properties across multiple length scales. Precise sequence control enables modulation of intrinsic molecular‐level physicochemical properties, including charge‐transport mobility [34], optical properties [35, 36], and glass‐transition temperature [37, 38], as well as nanoscale intermolecular interactions [39, 40] and self‐assembly behaviors [22, 23, 40], thereby expanding applications in optoelectronics and biomedicine. For example, random‐sequence copolymers based on poly(3‐hexylthiophene) backbones with fullerene side chains outperform block and gradient analogues in stabilizing organic photovoltaics, underscoring the significance of sequence effects in optoelectronic applications [41]. Furthermore, the Liu group has established quantitative correlations between polymer sequence and cellular uptake efficiency, pharmacokinetics, and biodistribution at both organ‐extract and tissue‐slice levels using MALDI–TOF MS and imaging within a single animal model, highlighting the critical role of sequence control in biomedicine [22]. However, the uncontrolled side‐chain sequence in GNRs essentially limited the regulation and optimization of GNR‐based nanostructures and properties [22, 23, 42] (Figure 1a). Therefore, it is timely and highly challenging to devise a reliable methodology toward the creation of structurally defined functional GNRs with precisely regulated side‐chain sequence for real‐world applications.

**FIGURE 2:**
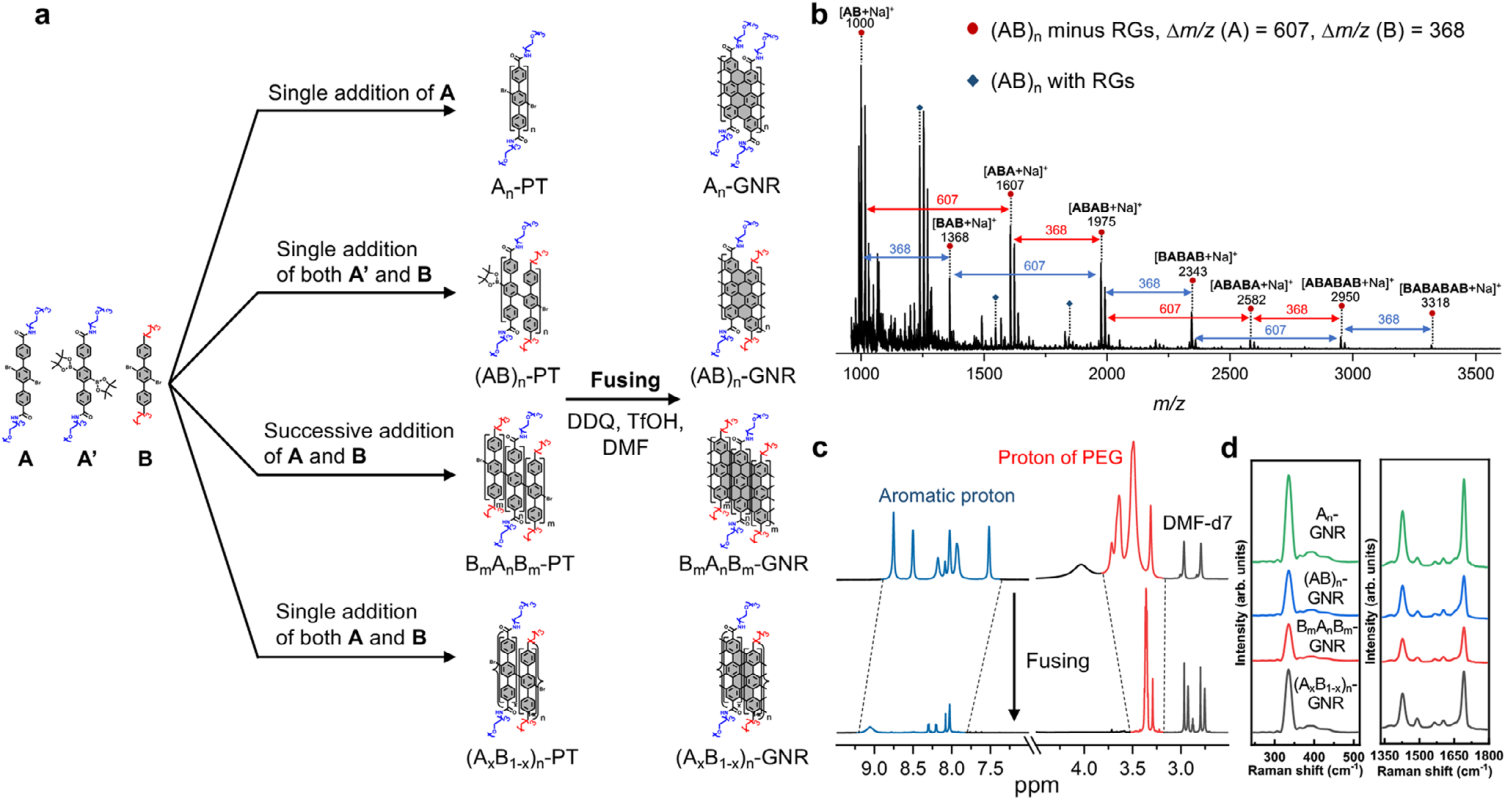
Synthesis and characterization of side‐chain sequence‐regulated GNRs. (a) Synthesis of diverse GNRs with distinct side‐chain sequences, including A_n_‐, (AB)_n_‐, B_m_A_n_B_m_‐, and (A_x_B_1‐x_)_n_‐GNR. (b) MALDI‐TOF‐MS of the precursor polyterphenyl with (AB)_n_ sequence ((AB)_n_‐PT), and the *m/z* peak intervals of A and B monomers were annotated. (c) High‐temperature ^1^H NMR spectra of A_n_‐GNR before and after cyclodehydrogenation in DMF‐d_7_. (d) Raman spectra of side‐chain sequence‐regulated GNRs (*λ_ex_* = 532 nm).

Herein, we report a robust liquid‐phase bottom‐up synthesis approach for high‐efficiency preparation of side‐chain sequence‐regulated functional GNRs via cross polymerization combining Yamamoto and Suzuki coupling of EG_3_‐ and pentane‐functionalized terphenyl‐based monomers, followed by subsequent cyclodehydrogenation (Figure 1a–c). This strategy enabled the precise regulation over the side‐chain sequence of functional GNRs, ultimately yielding four structurally defined functional GNRs with distinct side‐chain sequences, namely *homo*‐GNR (A_n_), *alternating*‐GNR ((AB)_n_), *block*‐GNR (B_m_A_n_B_m_), and *statistical*‐GNR ((A_x_B_1‐x_)_n_). The specific side‐chain sequence of the precursor of GNRs before fusing could be facilely decoded by mass spectrometry (MS) (Figure 2b). The cyclodehydrogenation process could be in‐time monitored using high‐temperature proton nuclear magnetic resonance spectroscopy (^1^H NMR) featuring an evident disappearance of the aromatic proton signals (Figure 2c), further verifying the formation of completely fused target GNRs. By meticulously controlling side‐chain sequence in GNRs, their hierarchically self‐assembled nanostructures could be readily regulated attributing to a unique sequence‐encoded molecular stacking mode, accompanied with subtly tunable optical properties. This synthetic approach paves a new avenue toward the integration of side‐chain structural and functional diversity into functional GNRs, making it a promising candidate for miscellaneous applications in optoelectronics, informatics, and pharmaceutics.

## 2 Results and Discussion

### 2.1 Synthesis of Side‐Chain Sequence‐Regulated GNRs

The liquid‐phase bottom‐up synthesis of side‐chain sequence‐regulated functional GNRs was depicted in Figure 2a. Initially, we attempted to synthesize the anthracene‐based monomer with site‐specific functional reactive groups (RGs). Unfortunately, the anthracene‐based monomer exhibited low polymerization activity possibly due to severe dehalogenation, leading to a trace of low‐molecular‐weight species even at 120°C for 7 days (Section S3). Thereupon, the side‐chain functionalized terphenyl‐based monomers with ether linkage were designed to exhibit improved polymerization activity. However, the cleavage of the ether linkage during the polymerization led to loss of control over the side‐chain sequence of GNRs. To this end, three side‐chain functionalized terphenyl‐based monomers with stable amide linkage (symbolized by A, A’, and B) were then synthesized (Section S4), and their identity was verified by ^1^H NMR, ^13^C NMR, and matrix‐assisted laser desorption/ionization time of flight mass spectrometry (MALDI‐TOF‐MS) (Figures S43–S50 and S57–S60).

These terphenyl‐based monomers A, A’, and B were subsequently polymerized in a given order to generate four side‐chain sequence‐regulated precursor polyterphenyls (PTs) via cross‐coupling, combining the Yamamoto reaction featuring AA‐type polymerization with the Suzuki reaction facilitating AB‐type polymerization. Yamamoto coupling is a nickel‐catalyzed carbon–carbon bond‐forming reaction that proceeds via a Ni(0) species, involving oxidative addition and subsequent reductive elimination. Suzuki coupling is a palladium‐catalyzed cross‐coupling between aryl boronic acids or esters and aryl halides, in which a base activates the boronic species, affording mild reaction conditions and broad functional‐group tolerance. First, the precursor *homo*‐PT with sole triethylene glycol (EG_3_) side chain (A_n_‐PT) was acquired in ∼56% yield via Yamamoto coupling catalyzed by Ni(COD)_2_, COD, and dipyridyl in DMF/PhMe (1:3, v/v) by a single addition of monomer A [43, 44, 45]. After 12 h, the HPLC peak of monomer A disappeared completely, yielding An‐PT and confirming near‐quantitative conversion prior to monomer B addition (Figure S37a). SEC analysis showed a corresponding shift to higher molecular weight, consistent with A_n_‐PT formation (Figure S37b), indicating preserved chain‐end reactivity. Upon addition of monomer B, A_n_‐PT was fully consumed within 24 h, producing higher‐molecular‐weight *triblock* B_m_A_n_B_m_‐PT with a clear SEC peak shift (Figure S37b). Consistently, ^1^H NMR revealed EG_3_ signals for An‐PT and additional pentane resonances for B_m_A_n_B_m_‐PT, confirming successful chain extension at both ends (Figure S37c). Afterwards, the precursor *triblock*‐PT (B_m_A_n_B_m_‐PT) was successfully fabricated in ∼51% yield via the sequential Yamamoto polymerization of monomer A and B (Section S16). The precursor *alternating*‐PT ((AB)_n_‐PT) composed of alternating EG_3_ and pentane side chains was ultimately obtained in ∼39% yield using Suzuki coupling catalyzed by Pd(PPh_3_)_4_, K_2_CO_3_, and Aliquat 336 in DMF/PhMe/H_2_O (2:2:1, v/v/v), in which pinacol boronic ester end groups in monomer A’ could merely react with bromine terminal groups in monomer B [46, 47, 48]. Similarly, the simultaneous addition of monomer A and B led to the formation of the precursor *statistical*‐PT ((A_x_B_1‐x_)_n_‐PT) in ∼56% yield via Yamamoto coupling. Integration of the ^1^H NMR signals from EG_3_ and pentane side‐chain termini in (A_x_B_1‐x_)_n_‐PT gave an EG_3_‐to‐pentane ratio of ∼1.05:1, consistent with the 1:1 feed ratio of monomers A and B (Figure S38). This result indicates comparable reactivity of monomers A and B under Yamamoto coupling conditions and confirms that side‐chain structure does not significantly affect terphenyl monomer reactivity.

The high‐temperature ^1^H NMR, mass spectroscopy, and size exclusion chromatography (SEC) were performed to characterize all the precursor PTs with meticulously steerable side‐chain sequences, further verifying their identities, structural characteristics, and defined side‐chain sequences (Figures S11 and S51–S56 and S61–S63). Notably, the encoded side‐chain sequence could be corroborated by conventional MS through the mass‐to‐charge ratio (*m/z*) analysis of a series of PT derivatives with varied chain lengths in polydisperse PTs (Figure 2b; Figures S61–S63), in which the *m/z* difference value (Δ*m/z*) between two neighbouring PT species was well consistent with the *m/z* value of either A unit (607) or B unit (368), respectively. To take (AB)_n_‐PT, for example (Figure 2b), the observed *m/z* value (1975) was in good agreement with the theoretical value of the ABAB‐PT molecular ion [M + Na]^+^ with the removal of RGs due to the fragmentation during the MALDI‐TOF‐MS test [49]. Given that Δ*m/z* of 607 and 368 were severally subtracted from this value, the observed ion peak values of 1368 and 1607 corresponded to the BAB‐PT and ABA‐PT molecular ion [M + Na]^+^, respectively. Similarly, when Δ*m/z* of 607 and 368 were severally added to this value, the observed ion peak values of 2582 and 2343 corresponded to the ABABA‐PT and BABAB‐PT molecular ion [M + Na]^+^, respectively. The aforementioned results manifested the successful preparation of versatile side‐chain sequence‐regulated functional GNRs via this liquid‐phase bottom‐up approach.

The subsequent cyclodehydrogenation of the resultant precursor PTs (A_n_‐, (AB)_n_‐, B_m_A_n_B_m_‐ and (A_x_B_1‐x_)_n_‐PTs) catalyzed by DDQ/TfOH in DMF led to the formation of side‐chain sequence‐regulated functional GNRs, including A_n_‐, (AB)_n_‐, B_m_A_n_B_m_‐ and (A_x_B_1‐x_)_n_‐GNRs, with isolated yields of ∼87%, 76%, 56%, and 71%, respectively. These sequence‐regulated functional GNRs exhibited superior solubility in common organic solvents, such as DMF and MeOH, possibly attributing to greatly decreased intermolecular π−π interactions between GNR segments by the grafted EG_3_ and pentane side‐chains [10, 18, 20]. The excellent solution processability enabled facile identification and validation of these structurally defined functional GNRs by liquid‐phase NMR (Figure 2c; Figures S51–S56) instead of solid‐phase NMR [10]. In Figure 2c, the high‐temperature ^1^H NMR spectrum of A_n_‐PT in deuterated DMF displayed stronger proton signals in the aromatic and PEG regions. Following cyclodehydrogenation, the solubility of the A_n_‐GNR in deuterated DMF decreased markedly, leading to the almost complete disappearance of its aromatic proton signals, while the corresponding PEG‐related proton peaks became distinctly sharper (Figure 2c). For the other side‐chain‐sequenced GNRs, deuterated toluene was used as the solvent owing to the presence of pentyl side chains, which rendered them completely insoluble in DMF. Similarly, the subsequent cyclodehydrogenation caused a pronounced reduction or nearly complete disappearance of both aromatic proton signals and PEG‐related proton peaks in (AB)_n_‐, B_m_A_n_B_m_‐ and (A_x_B_1‐x_)_n_‐GNRs (Figures S51–S56).

Raman spectra of these side‐chain sequence‐regulated functional GNRs further revealed typical D and G peaks (Figure 2d; Figure S21), and were not highly dependent on the side‐chain sequence of GNRs [18, 50]. Notably, a distinct peak, corresponding to the radial breathing‐like mode (RBLM), was observed at around 336 cm^−1^ for all four GNRs, verifying the characteristic structure of GNRs. Employing the relation: *w* = 3222/*v*_RBLM_, where *w* (Å) is the width of GNR and *v*_RBLM_ is the wavenumber of RBLM, the experimentally estimated mean width of these GNRs was approximately 0.96 nm, which aligned well with the calculated value of 1 nm [3, 18] (Figure 1c). In addition, compared with UV–vis and photoluminescence (PL) spectra of GNRs before fusing, obvious bathochromic shifts by enhanced electron delocalization through the extendedly π‐conjugated system substantiated the successful cyclodehydrogenation to accomplish accurate sequence regulation in the resultant functional GNRs [51, 52] (Figures S12–S15). Fourier transform infrared (FTIR) analysis revealed remarkable attenuation of the signals from aromatic C‐H stretching vibrations after fusing, further demonstrating the successful cyclodehydrogenation [3, 18, 50] (Figures S16–S20).

### 2.2 Sequence‐Encoded Nanostructures from Structurally Defined GNRs

**FIGURE 3:**
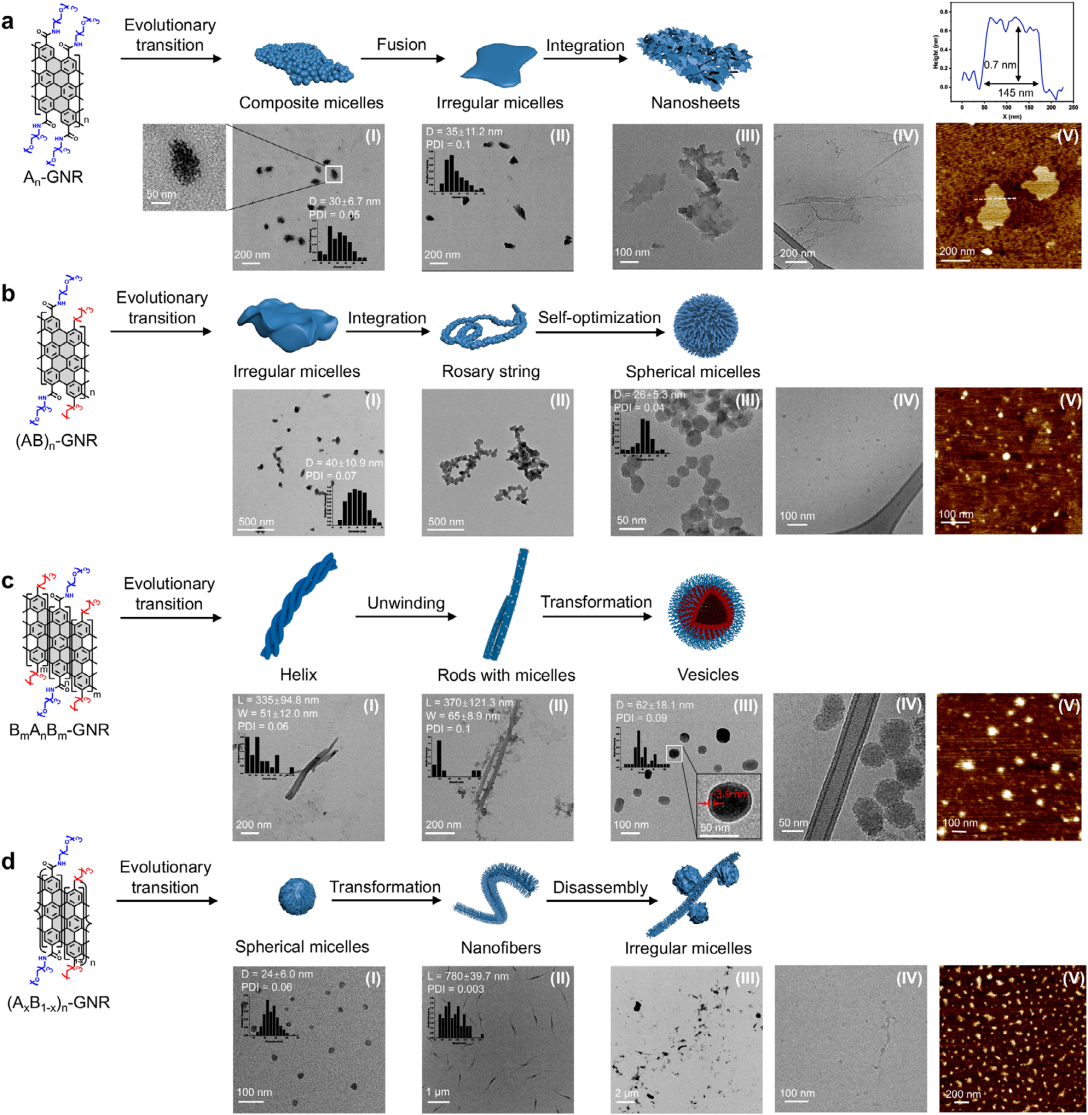
Sequence‐encoded hierarchical nanostructures of structurally defined GNRs. (a‐d) TEM (I‐III), Cryo‐TEM (IV), and AFM images (V) of nanostructures self‐assembled from side‐chain sequence‐regulated GNRs, including A_n_‐GNR (a), (AB)_n_‐GNR (b), B_m_A_n_B_m_‐GNR (c), and (A_x_B_1‐x_)_n_‐GNR (d) in H_2_O/DMF solutions of varied volume proportions, including 50% H_2_O (I), 75% H_2_O (II), and 100% H_2_O (III‐V). AFM height profile from AFM image in a‐V.

The self‐assembly behaviours of flexible polymers have been demonstrated to be highly sequence‐dependent [24, 25, 53, 54]. We aimed to explore the impact of side‐chain sequence regulation in functionalized GNRs on their self‐assembled nanostructures in solution. Transmission electron microscopy (TEM), cryo‐transmission electron microscopy (Cryo‐TEM), and atomic force microscopy (AFM) were employed to comprehensively analyze the morphologies and sizes of the resulting nanoscale assemblies (Figures S22–S35). First, TEM images clearly showed A_n_‐GNR self‐assembled into composite micelles in 50% aqueous solution (Figure 3a‐I). With increasing water content in H_2_O/DMF to 75%, the composite micelles were fused to form irregular micelles, which tended to ulteriorly integrate into nanosheets in aqueous solution (Figure 3a‐II/III). Cryo‐TEM characterization further confirmed that the A_n_‐GNR self‐assembles into nanosheets in aqueous solution (Figure 3a‐IV). AFM observations corroborated the nanosheet morphology of A_n_‐GNR assemblies in aqueous solution with an average thickness of ca. 0.7 nm (Figure 3a‐V). In contrast, (AB)_n_‐GNR exhibited distinct irregular micellular nanostructures in 50% H_2_O, which further merged into rosary‐string‐like assemblies in 75% H_2_O (Figure 3b‐I/II). Then, the self‐assemblies of (AB)_n_‐GNR underwent spontaneous transformation to form spherical micelles with a mean diameter of 26±5.3 nm in aqueous solution (Figure 3b‐III). Additionally, Cryo‐TEM and AFM images collectively verified that (AB)_n_‐GNR‐derived assemblies adopt a spherical micellar morphology in aqueous solution (Figure 3b‐IV/V).

Both B_m_A_n_B_m_‐GNR and (A_x_B_1‐x_)_n_‐GNR, whose stacking mode altered with the increase of water proportion (Figure 5c,d), demonstrated a significant morphology transformation in solution. The triblock GNR (B_m_A_n_B_m_‐GNR) self‐assembled into spring‐like helices with a mean length of 335±94.8 nm and width of 51±12.0 nm in 50% H_2_O solution (Figure 3c‐I). With the increase of water proportion to 75%, the helices underwent progressive unwinding and were converted into nanorods along with small micelles, revealing a mean length of 370±121.3 nm and width of 65±8.9 nm (Figure 3c‐II). Finally, the self‐assembly morphology of B_m_A_n_B_m_‐GNR underwent an evolutionary transformation from rod‐like structures to vesicles with an average diameter of 62±18.1 nm and a shell thickness of around 3.9 nm in water (Figure 3c‐III). Cryo‐TEM and AFM images further validated that B_m_A_n_B_m_‐GNR assemblies exhibit a vesicular morphology in aqueous solution (Figure 3c‐IV/V). In particular, the significant morphological transformation of assemblies might be attributed to the alteration of the stacking modes in varied H_2_O/DMF solutions (Figure 5c). (A_x_B_1‐x_)_n_‐GNR exhibited spherical micellular structures featuring an average diameter of 24±6.0 nm in 50% H_2_O solution, and it demonstrated an evolutionary transformation to nanofibers with a mean length of 780±39.7 nm in 75% aqueous solution (Figure 3d‐I/II). Subsequently, (A_x_B_1‐x_)_n_‐GNR further underwent a disassembly process to form irregular micelles in aqueous solution (Figure 3d‐III). Furthermore, Cryo‐TEM and AFM confirmed the irregular micellar structure of (A_x_B_1‐x_)_n_‐GNR assemblies in aqueous media (Figure 3d‐IV/V). Images of the aqueous GNR dispersions showed macroscopic homogeneity, with all samples remaining clear and transparent without visible precipitation after 60 days (Figure S35a). Tyndall effect images further confirmed sustained dispersion in the aqueous phase, indicating stable colloidal behavior. Together, these observations demonstrate the long‐term macroscopic colloidal stability of the GNR dispersions.

### 2.3 Formation Mechanism of Hierarchical Nanostructures

**FIGURE 4:**
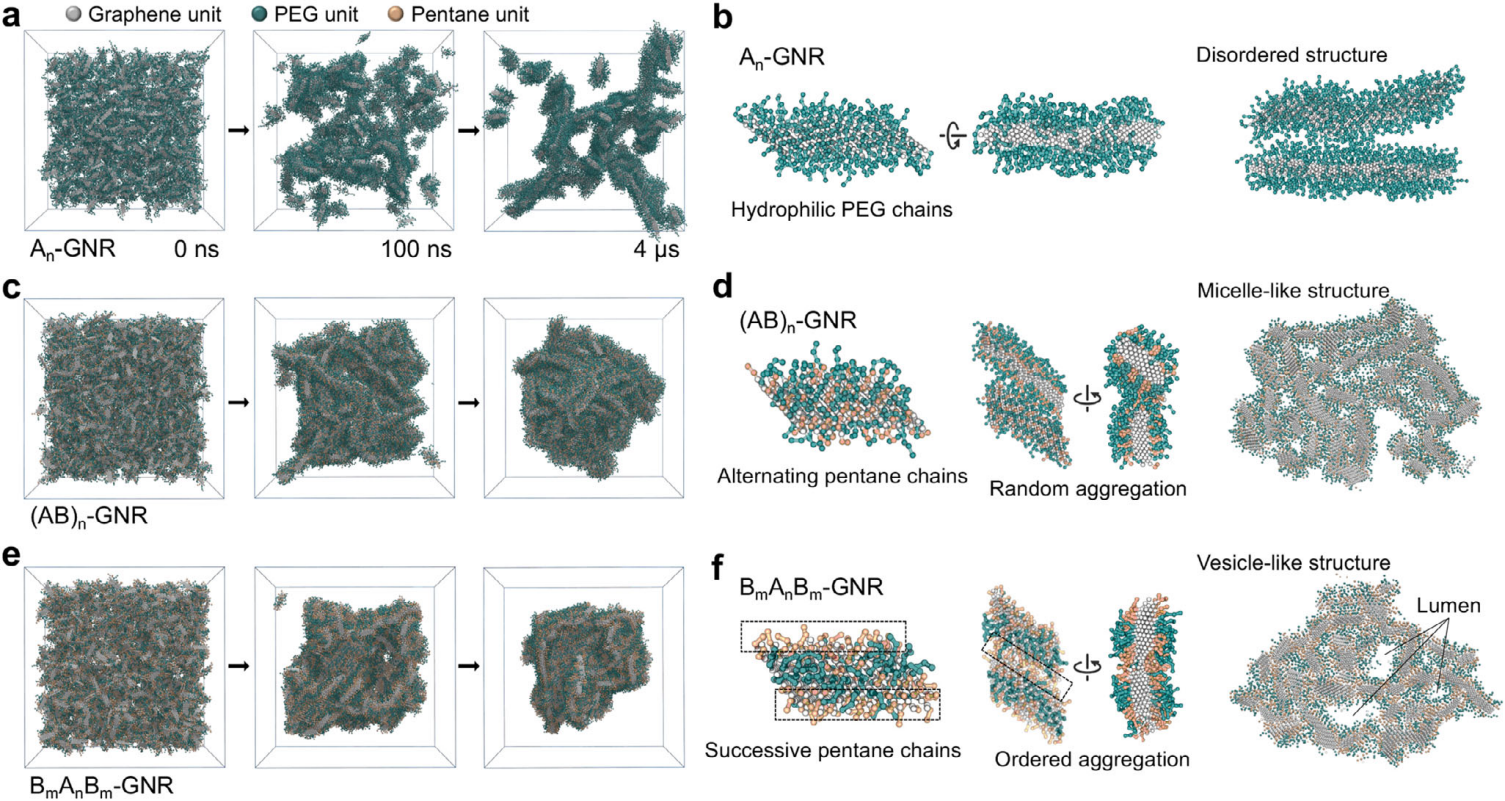
Self‐assembly mechanism of hierarchical nanostructures by coarse‐grained MD simulation. (a, c, e) Solution self‐assembly of side‐chain sequence‐regulated GNRs, including A_n_‐GNR(a), (AB)_n_‐GNR (c), and B_m_A_n_B_m_‐GNR (e), and (b, d, f) their distinctive stacking modes. The snapshots were extracted at 0 ns, 100 ns, and 4 µs of simulation trajectories. The planar graphene, hydrophilic PEG, and hydrophobic pentane segments were shown in white, green, and orange spheres, respectively.

To further elucidate the self‐assembly mechanism of side‐chain sequence‐regulated GNRs in solution, we conducted coarse‐grained molecular dynamics (CG‐MD) simulations using the MARTINI 3 force field [55] to investigate the self‐assembly processes of A_n_‐, (AB)_n_‐, and B_m_A_n_B_m_‐GNR in aqueous solution (Figure S41). The results show that all three GNRs rapidly aggregated into short nanorods, driven by strong hydrophobic interactions between the graphene segments of side‐chain functionalized GNRs. However, the distinctive side‐chain sequence effectively regulated the stacking modes between GNR‐based nanorods, leading to the formation of versatile self‐assembled morphologies. For A_n_‐GNR, the successive EG_3_ side‐chains were located on the surface of the nanorods, generating a hydrophilic shell to prevent A_n_‐GNR nanorods from aggregating into hierarchically ordered assemblies (Figure 4a,b).

For (AB)_n_‐ and B_m_A_n_B_m_‐GNRs containing hydrophobic pentane side‐chains, the resultant side‐chain shell was far more hydrophobic than that of A_n_‐GNR‐based nanorods, thereby enabling the formation of spherical assemblies (Figure 4c–f). Compared with (AB)_n_‐GNR with alternating pentane/EG_3_ side‐chain sequence, the block B_m_A_n_B_m_‐GNR first assembled into amphiphilic nanorods, which further drive the ordered aggregation of nanorods into a vesicle‐like nanostructure containing typical lumina instead of a micelle‐like architecture generated by (AB)_n_‐GNRs (Figure 4c–f). As the water fraction increased from 50% to 100%, B_m_A_n_B_m_‐GNR assemblies transitioned from helices to vesicles, indicating morphology is governed primarily by solute–solvent interactions. Dimerization free‐energy calculations in pure DMF and water show that dimers stabilized by hydrophobic graphene‐core interactions are thermodynamically favored in both solvents (center‐of‐mass distance ≈ 0.35–0.5 nm; Figure S42a). Variation in solvent composition can reshape the free‐energy landscape by introducing metastable states, and the coexistence of rods and micelles in DMF/water (25/75) is likely due to kinetic trapping. Therefore, the CG‐MD simulation demonstrated that the precise manipulation of side‐chain sequences of functional GNRs empowers facile and delicate regulation of self‐assembly behaviours in solution, attributing to their sequence‐encoded stacking modes.

### 2.4 Optical Properties of GNR‐Based Nanostructures

**FIGURE 5:**
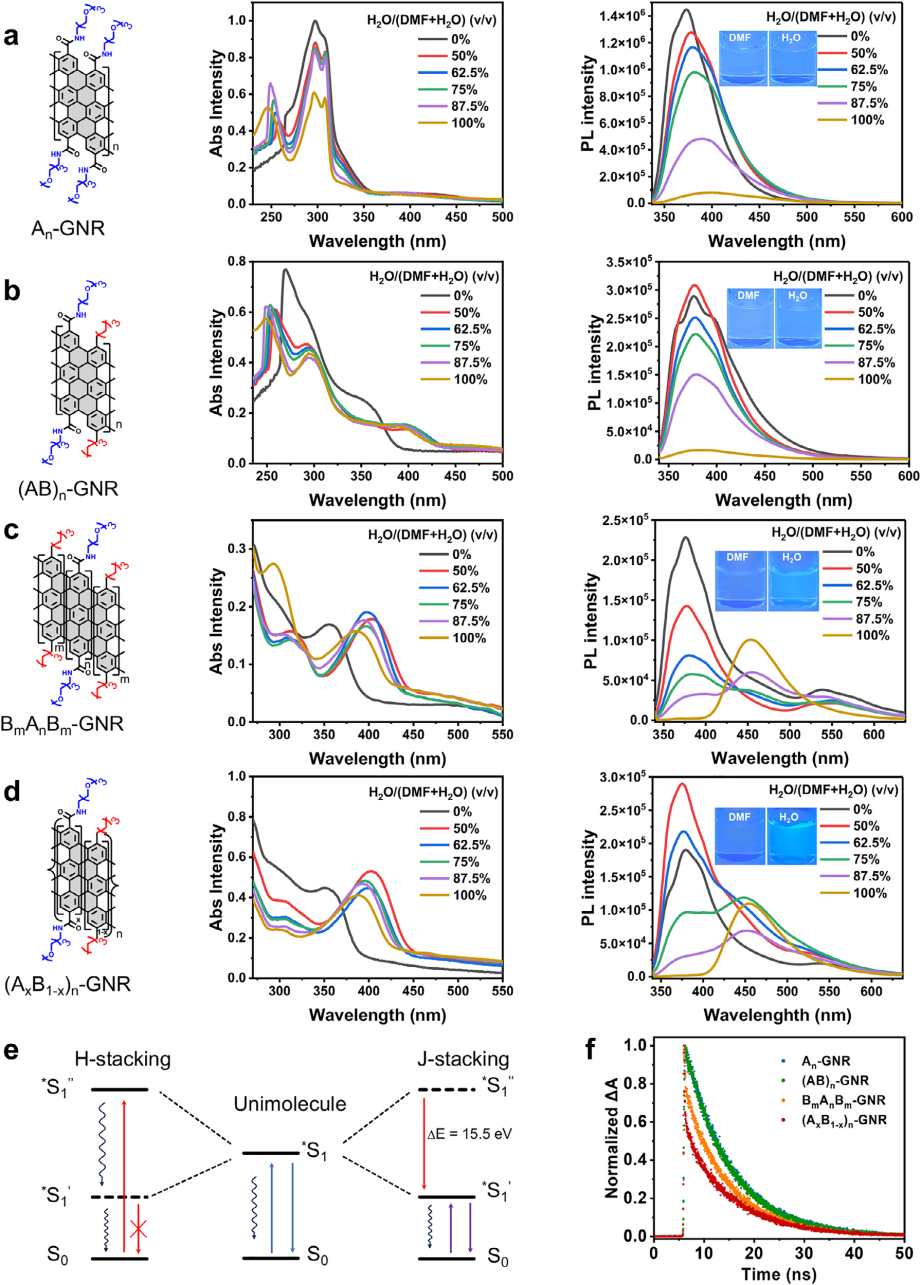
Tunable optical properties of side‐chain sequence‐regulated GNR‐based nanostructures. (a‐d) UV–vis absorption spectra (left) and steady‐state fluorescence spectra (right) of side‐chain sequence‐regulated GNRs, including A_n_‐GNR (a), (AB)_n_‐GNR (b), B_m_A_n_B_m_‐GNR (c), and (A_x_B_1‐x_)_n_‐GNR (d) in H_2_O/DMF mixed solutions. (e) Proposed energy‐level diagram of diverse side‐chain sequence‐regulated GNRs in their unimolecular state and H‐ and J‐aggregated stacking modes. (f) Transient time‐resolved photoluminescence spectra of side‐chain sequence‐regulated GNRs in aqueous solution.

To investigate the influence of side‐chain sequence in GNRs on the optical properties of these resultant GNR‐based nanostructures in solution, UV–vis absorption spectroscopy, steady‐state photoluminescence (PL) spectroscopy, and transient time‐resolved photoluminescence spectroscopy (PL) were conducted (Section S15). In Figure 5a,b, no evident peak shift in UV–vis absorption spectra was observed for both A_n_‐ and (AB)_n_‐GNRs in varied mixed solutions, demonstrating that the variation of water content in solutions did not remarkably alter the molecular stacking modes of A_n_‐ and (AB)_n_‐GNRs. With increasing water content in H_2_O/DMF systems, the PL spectra of both A_n_‐GNR and (AB)_n_‐GNR in solutions exhibited a significant fluorescence quenching along with almost invariable FL wavelength, signifying the predominant H‐stacking mode in aqueous solution, verified by continuously self‐assembled morphological transitions as mentioned above.

In Figure 5c,d, the characteristic UV–vis absorption peaks of both B_m_A_n_B_m_‐ and (A_x_B_1‐x_)_n_‐GNRs demonstrated a remarkable red‐shift from ca. 356 nm to ca. 400 nm with the increase of water content in H_2_O/DMF solutions, confirming the conversion of the predominant stacking mode from H‐stacking to J‐stacking. With increasing water content from 0% to 100% in H_2_O/DMF solutions, both B_m_A_n_B_m_‐ and (A_x_B_1‐x_)_n_‐GNRs revealed a slight fluorescence quenching accompanied with a notable red‐shift spanning from 375 to 455 nm. To assess the relationship between block length and PL red shift, the B‐block length in B_m_A_n_B_m_‐GNR was increased by varying monomer B from 1.0 to 2.0 equiv. This increased the A/B block‐length ratio from ∼1:0.42 to ∼1:0.79 (Figure S39a,b). However, no appreciable PL peak shift was observed, indicating no direct quantitative correlation between block length and PL red shift (Figure S39c). Furthermore, transient time‐resolved PL spectroscopy revealed that the lifetimes of both B_m_A_n_B_m_‐ and (A_x_B_1‐x_)_n_‐GNRs were shorter than those of A_n_‐ and (AB)_n_‐GNRs in aqueous media, demonstrating that B_m_A_n_B_m_‐ and (A_x_B_1‐x_)_n_‐GNRs adopt a J‐aggregated stacking mode (Figure 5f). In Figure S36a, A_n_‐ and (AB)_n_‐GNRs predominantly adopt H‐stacking, showing negligible shifts in UV–vis and PL spectra and pronounced PL quenching, consistent with dominant nonradiative decay in which excited states relax by dissipating energy as heat. In contrast, BmAnBm‐ and (A_x_B_1‐x_)_n_‐GNRs preferentially adopt J‐stacking, exhibiting clear red shifts in UV–vis (30 and 38 nm) and PL spectra (77 and 76 nm) without significant quenching, consistent with predominantly radiative decay, in which excited electronic states relax via photon emission, leading to a lower‐energy, more stable state. Correspondingly, updated energy‐level diagrams include the numerical optical energy variation for J‐stacking (≈15.5 eV; Figure 5e; Figure S36b), and the wavelength‐to‐photon‐energy conversion formula is provided in Section S15. Transient PL measurements further show that B_m_A_n_B_m_‐ and (A_x_B_1‐x_)_n_‐GNRs possess much shorter radiative lifetimes (0.24 and 0.18 ns) than A_n_‐ and (AB)_n_‐GNRs (8.9 and 9.1 ns), confirming the dominance of J‐stacking behavior (Figure S36c). The variation in optical behaviors of GNRs was highly dependent on their corresponding stacking mode in solutions, which was precisely in line with the evolutionary morphological transformation of the resultant hierarchical assembled nanostructures (Sections S17 and S18). The PL spectra of side‐chain‐regulated GNRs remained stable over 60 days, whereas their UV–vis spectra showed sequence‐dependent intensity variations, likely due to gradual colloidal aggregation sensitive to dispersion conditions (Figure S35b,c). B_m_A_n_B_m_‐GNR exhibited the largest change, highlighting sequence‐dependent optical behavior and colloidal stability. Stability may be further improved by optimizing side‐chain sequences, introducing longer or additional PEG chains [19], or employing non‐covalent modifiers such as surfactants (cetyltrimethylammonium bromide, CTAB, and sodium dodecyl sulfate, SDS) to suppress π–π stacking and enhance aqueous dispersion [56, 57].

**FIGURE 6:**
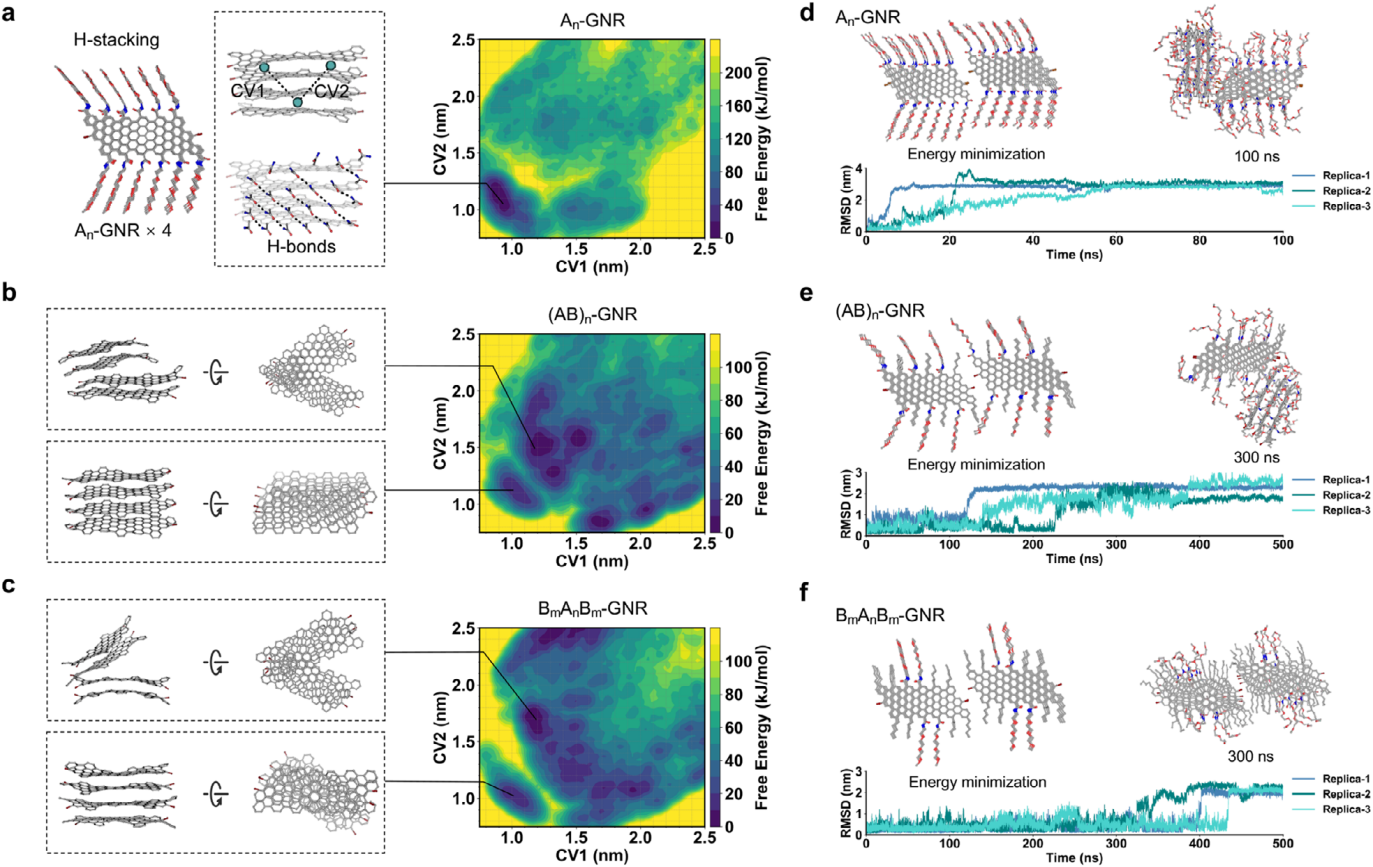
Molecular stacking mechanism by all‐atom MD simulation. (a–c) H‐aggregated stacking free energy surfaces (FES) of A_n_‐GNR (a), (AB)_n_‐GNR (b), and B_m_A_n_B_m_‐GNR (c). Representative conformations are shown in the left panels with side‐chains omitted for clarity. (d‐f) Stability of J‐aggregated stacking model for A_n_‐GNR (d), (AB)_n_‐GNR (e), and B_m_A_n_B_m_‐GNR (f) as revealed by MD simulations.

To further elucidate the effect of the molecular stacking mode of side‐chain sequence‐regulated GNRs on their optical properties, we initially built H‐stacking models consisting of four GNR molecules for A_n_‐GNR, (AB)_n_‐GNR, and B_m_A_n_B_m_‐GNR, respectively. The on‐the‐fly probability enhanced sampling (OPES) method [58] was employed to explore GNR conformations and evaluate the stacking free energy. Two distances between the centers of mass (COMs) of the GNRs were biased as collective variables (CVs) to enhance conformational sampling (Figure 6a). The free energy surface (FES) of the A_n_‐GNR model exhibited a single metastable state, in which the four GNR molecules packed together in a layer‐by‐layer mode (H‐stacking). However, the FES of (AB)_n_‐ and B_m_A_n_B_m_‐GNR models showed several metastable states where GNR molecules partially disassembled (Figure 6b,c), indicating a relatively weak H‐stacking mode compared with the A_n_‐GNR. The stability of the H‐stacking mode in A_n_‐GNR was attributed to the extensive interlayer hydrogen bonds between the amide groups of EG_3_ side‐chains (Figure 6a), and the number of hydrogen bonds formed between the H‐stacked GNR models was quantified (Figure S41c). A pre‐stacked A_n_‐GNR model of 14 molecules and an A^ester^_n_‐GNR analogue with amide groups replaced by esters was constructed. After 500 ns of MD simulation, the A_n_‐GNR stack remained stable and ordered, whereas the A^ester^_n_‐GNR stack showed pronounced bending (Figure S42b). These results indicate that π–π and hydrophobic graphene‐core interactions are sufficient to drive GNR assembly, while interlayer hydrogen bonds play a reinforcing role in stabilizing larger GNR stacks. In addition, we also established J‐stacking models consisting of two adjacent GNR trimers and performed unbiased MD simulations to evaluate the stability (Figure 6d–f). The root‐mean‐square deviation (RMSD) demonstrated that the J‐stacking model of B_m_A_n_B_m_‐GNR remained stable for at least 300 ns during MD simulations. In contrast, the A_n_‐ and (AB)_n_‐GNR models collapsed much faster, such as ∼10 ns for A_n_‐GNR and ∼100–200 ns for (AB)_n_‐GNR, respectively (Figure 6d,e). The ability of B_m_A_n_B_m_‐GNR to form J‐stacking mode arised from the hydrophobic interactions strengthened by the successive hydrophobic pentane side‐chains. All atom MD simulation demonstrated that B_m_A_n_B_m_‐GNR had the strongest ability to form J‐stacking mode (Figure 6c,f), which accounts for the substantial red‐shift (from 375 to 455 nm) and align with the preceding PL spectroscopic findings (Figure 5c,e). Uncontrolled GNR assemblies in solution typically exhibit severe fluorescence quenching [18], whereas J‐type stacking enables red‐shifted emission without quenching, supporting bioimaging applications. In addition, side‐chain‐functionalized GNRs disperse well in common organic solvents, enabling uniform spin‐coated films on glass substrates (Figure S40). Therefore, these functional GNRs with exquisitely regulated side‐chain sequences represent a viable and robust strategy to attain highly tunable optical characteristics via rational manipulation of molecular stacking modes, ultimately facilitating efficient and precise modulation of their physicochemical properties and functionalities in practical settings.

## 3 Conclusions

We have successfully exploited a feasible liquid‐phase bottom‐up synthesis strategy toward the creation of structurally defined functional GNRs with delicately regulated side‐chain sequences. Benefiting from the precisely regulated sequence of hydrophilic EG_3_ and hydrophobic pentane side‐chains grafted onto the rigid GNR backbone geometry, the resultant side‐chain sequence‐regulated GNRs demonstrated readily controllable hierarchical nanostructures accompanied with exquisitely tunable optical properties. Both all‐atom and coarse‐grained MD simulations were conducted to elucidate the structural dynamics of the resultant side‐chain sequence‐regulated functional GNRs, further demonstrating their unique sequence‐encoded molecular stacking mode, leading to precise manipulation of hierarchical nanostructures and optical properties. We envision that this work will pave an avenue for the development of hierarchically ordered GNR‐based nanostructures with precisely controlled morphologies and functions, and holds boundless potential for optoelectronic and biomedical applications.

## Author Contributions

R.D. conceived and supervised this project. R.D., B.C., and L.Y. designed the experiments. B.C. performed the synthesis and characterization of polymers. B.C. conducted the UV–vis and fluorescence spectrometry measurements. K.S. performed the MD simulations. R.D., B.C., K.S., L.Y., L.Y., Y.W., Z.Z., Y.Z., and X.Z. analysed the data. R.D., B.C., and K.S. wrote the manuscript. R.D. guided the project. All of the authors discussed the results and edited the manuscript.

## Funding

This work was financially supported by the National Natural Science Foundation of China (22375124 and 22175114), the Natural Science Foundation of Shanghai (22ZR1429400), the National Science Fund for Excellent Young Scholars (Overseas) (23Z990202541), the High‐Level Overseas Talents Introduction Program of Shanghai, the Research Startup Foundation of Shanghai Jiao Tong University, Taizhou High‐level Innovative and Entrepreneurial Talent Program, and the “Green Valley Elite‐Innovation Leading Action” Program of Zhejiang.

## Conflicts of Interest

The authors declare no conflicts of interest.

## Supporting information




**Supporting File**: advs74208‐sup‐0001‐SuppMat.pdf.

## Data Availability

The data that support the findings of this study are available from the corresponding author upon reasonable request.
