# Supplementary material for: Controlled Optically Active Hierarchical Nanostructures of Side‐Chain Sequence‐Regulated Graphene Nanoribbons
Source: Adv Sci (Weinh). 2026 Feb 3;13(20):e23362. doi: 10.1002/advs.202523362 (PMC13067861; doi:10.1002/advs.202523362)
Supplement: Supplementary file 1 — Supporting File: advs74208‐sup‐0001‐SuppMat.pdf. [file ADVS-13-e23362-s001.pdf]

Controlled Optically Active Hierarchical Nanostructures of Side-Chain Sequence-Regulated Graphene Nanoribbons

Baiyang Chen[1†], Kaiyuan Song[1†], Li Yu[1†], Lujia You[1], Yiming Wang[1], Zhaoguo Zhang[2], Yongfeng Zhou[2], Xinyuan Zhu[2], and Ruijiao Dong[1]*

[1] Key Laboratory of Systems Biomedicine (Ministry of Education), Shanghai Center for Systems Biomedicine, Shanghai Jiao Tong University, 800 Dongchuan Road, Shanghai 200240, China

[2] School of Chemistry and Chemical Engineering, Frontiers Science Centre for Transformative Molecules, Shanghai Key Laboratory for Molecular Engineering of Chiral Drugs, Shanghai Jiao Tong University, 800 Dongchuan Road, Shanghai 200240, China

* e-mail: drj021@sjtu.edu.cn

[†] These authors contributed equally.

Table of content

1. Materials ... **2**

2. Characterization and measurements ... **3**

3. Failed synthesis route for site-specific functionalized monomers ... **6**

4. Synthesis of terphenyl-based monomers with site-specific functional RGs ... **12**

5. Synthesis of sequence-regulated graphene nanoribbon, A_n-GNR ... **16**

6. Synthesis of sequence-regulated graphene nanoribbon, $(AB)_n$-GNR ... **17**

7. Synthesis of sequence-regulated graphene nanoribbon, $B_mA_nB_m$-GNR ... **18**

8. Synthesis of sequence-regulated graphene nanoribbon, $(A_xB_{1-x})_n$-GNR ... **20**

9. Supplementary SEC spectra ... **21**

10. Supplementary UV-vis and PL spectra ... **21**

11. Supplementary FTIR spectra ... **23**

12. Supplementary Raman spectra ... **27**

13. Supplementary TEM images ... **28**

14. Solubility and long-term stability of sequence-regulated GNRs ... **35**

15. Quantitative analysis of UV-Vis, PL and transient PL ... **38**

16. Synthesis of the triblock precursor $B_mA_nB_m$-PT and $(A_xB_{1-x})_n$-PT ... **40**

17. Correlation between the block length and the red-shift degree of PL ... **42**

18. Potential applications in fields of biomedicine and optoelectronics. ... **44**

19. Molecular dynamics simulations ... **45**

20. Supplementary NMR spectra ... **49**

21. Supplementary mass spectra ... **56**

22. Supplementary references ... **59**

1. Materials

4-Carboxylphenylboronic acid pinacol ester (Reagent grade, ≥99%, Admas), 2-(2-(2-methoxyethoxy)ethoxy)ethan-1-amine (Reagent grade, 97%, Admas), 1-(3-dimethylaminopropyl)-3-ethylcarbodiimide (Reagent grade, ≥98%, Admas), hydroxybenzotriazole (Reagent grade, 99%, Admas), 1,4-dibromo-2,5-diiodobenzene (Reagent grade, 98%, Admas), tetrakis(triphenylphosphine)palladium (Reagent grade, ≥99.9% metals basis, Admas), potassium carbonate (Reagent grade, 99.99%, Admas), Aliquat 336 (Reagent grade, ≥90%, Admas), bis(pinacolato)diboron (Reagent grade, ≥98%, Admas), [1,1'-bis(diphenylphosphino)ferrocene]dichloropalladium(II) (Reagent grade, ≥99.9% metals basis, Admas), potassium acetate (Reagent grade, ≥99%, Admas), 4-*n*-pentylbenzeneboronic acid (Reagent grade, 98%, Admas), bis(1,5-cyclooctadiene)Nickel(0) (Reagent grade, 97%, Admas), 1,5-cyclooctadiene (Reagent grade, 99%, stabilized with 50-150ppm TBC, Admas), 2,2'-bipyridine (Reagent grade, 99%, Admas), 2,3-dichloro-5,6-dicyano-1,4-benzoquinone (Reagent grade, 98%, Admas), and trifluoromethanesulfonic acid (reagent grade, ≥99%, Admas) were used as received.

N,N-Dimethylformamide (DMF), dichloromethane (CH_2Cl_2), tetrahydrofuran (THF), acetonitrile (MeCN), methanol (MeOH) and toluene (PhMe) for anhydrous reactions were from Admas, and dried over baked 4A molecular sieves. All reactions were conducted under argon, unless specified. Ethanol (EtOH), *tert*-butyl alcohol (*t*BuOH), diethyl ether (Et_2O), acetone, chloroform ($CHCl_3$), ethyl acetate (EtOAc), carbon tetrachloride (CCl_4), petroleum ether (PE) and *n*-heptane etc. (Admas) were used as received.

2. Characterization and measurements

Nuclear Magnetic Resonance (NMR). ^1H NMR spectra were recorded at 400 MHz on a Bruker AVANCE III HD 400 NMR spectrometer. NMR spectra were measured at 298 K in $CDCl_3$, DMSO-d_6 or at 378 K in Tolune-d_8. Chemical shifts are given in ppm relative to the signals corresponding to the residual non-deuterated solvents: $CDCl_3$, δ_H = 7.26 ppm; DMSO-d_6, δ_H = 2.50 ppm; Tolune-d_8, δ_H = 7.09 ppm. Note: Dmtr is an acid-labile protecting group, and trace Et_3N was usually added into the NMR samples ($CDCl_3$ as solvent) in order to maintain the stability of all Dmtr-protected compounds.

MALDI-TOF-MS. Samples were analyzed using a Bruker 7.0T SolariX Fourier transform ion cyclotron resonance mass spectrometry (Bruker Daltonics, Germany), equipped with MALDI ion source, this MALDI source has a pulsed smartbeam-II UV laser with an attenuator that allows fine adjustment of laser fluency (355 nm, Azura Laser AG, Berlin, Germany). The scanning range is *m/z* 150.48–3,000 in positive ion mode. The MS instrument was tuned and calibrated with Bruker peptide II standards in a matrix mix (CHCA) in MALDI source. The laser beam focus was set at the small value. The detector plate was 210 V, plate offset was 80 V, laser power was 20%–30%, frequency was 500 Hz; laser shots were 500, time of light was 1.1 ms. MALDI-TOF-MS data were viewed and processed using Bruker DataAnalysis 5.0.

ESI FT-ICR MS. The MS analysis was carried out on a 7.0 T Bruker SolariX FT-ICR mass spectrometer coupled with a positive-ion ESI. Samples were injected into the ionization source through a syringe pump. The mass range was set at *m/z* 150−2000. The data size was set to 1M words. Capillary voltage: 4000V; Nebulizer gas: 2.0 bar, dry gas: 4.0L/min, dry temperature: 220 ℃, end plate offset is 800V, RF frequency is 2 MHz. Time of flight is 1.0 ms. Sodium formate solution (10% formic acid: 0.1 mol/L sodium hydroxide/, acetonitrile: 1:1:8) was used for calibration. DataAnalysis 5.0 software was used for mass spectral viewing and processing.

UV-vis absorption spectrometry. The UV-vis absorption spectra were recorded on a Shimadzu UV 3600 spectrometer. The slit-width was set as 1 nm, and scan speed was set as 480 nm/min.

The wavelength range was from 200 to 700 nm.

Steady-state fluorescence spectrometry. Fluorescence emission spectra were collected on a Shimadzu RF-6000 spectrofluorophotometer. The excitation wavelength of the samples was set at 325 nm. The slit-width was set as 2 nm, and the scan speed was set as 480 nm/min. The fluorescence range was from 300 to 800 nm.

Time-resolved fluorescence spectroscopy. Fluorescence lifetimes were measured using a Horiba Scientific DeltaFlex time-correlated single-photon counting (TCSPC) system equipped with pulsed NanoLED sources. The excitation wavelength was set to 325 nm, and emission was collected at 425 nm. Photon decay curves were fitted using a tri-exponential tail-fitting model until optimal χ^2 values, standard deviations, and residuals were achieved. During tail fitting, the ultrafast short-lived component was neglected. The average lifetime was calculated using an amplitude-weighted method, expressed as:

$$\tau_{av} = (\Sigma\ (\tau_i * A_i)) / \Sigma A \ldots\ldots\ldots\ldots \text{(i)}$$

where τ_{av} is the average lifetime, A is the amplitude, τ_i and A_i are the lifetime and amplitude of the ith component.

Size exclusion chromatography (SEC). The SEC analyses of the sequence-regulated PTs were carried out on an Agilent 1260 Infinity II-instrument containing a refractive index detector using DMF with high-performance liquid chromatography (HPLC) grade as an eluent, at a flow rate of 1.0 mL min^{-1} at 40 °C. One PLgel 5 μm Guard and two PLgel 5 μm Mixed D columns were used in series. Linear polystyrene standards were used for calibration: All sequence-regulated PTs samples were diluted with HPLC-grade DMF and filtered through a polytetrafluoroethylene (PTFE) membrane with a 0.2 μm pore size prior to injection (100 μm).

High performance liquid chromatography (HPLC). HPLC analysis was performed on a Classical 3200 Elite HPLC system equipped with an ultraviolet diode array detector using a reverse-phase C4-300 column (250 mm × 4.6 mm, ACE Hichrom). The mobile phases for HPLC were deionized water (solvent A) and tetrahydrofuran (solvent B). The samples were

injected using an Elite ECLASSICAL S3200 autosampler, and the injected volume was 20 μl. The samples were eluted with 15% solvent B followed by a linear gradient to 100% solvent B over 35 min, and the column back to 15% solvent B over 5 min. The HPLC pump flow rate was set at 1 mL min^{-1}, and the column temperature was kept at 40 °C. The detector was an Elite ECLASSICAL D3200 variable-wavelength detector and the eluent was monitored at 325 nm.

Transmission electron microscopy (TEM). TEM observations were performed on a Talos L120C G2 (ThermoFisher Scientific, USA) with an accelerating voltage of 120 kV. TEM samples were obtained by dropping solutions of GNR-based nanoassemblies (50 μg/mL) onto carbon-coated copper grids, followed by removing excess liquid with filter paper after 5 min, and no staining treatment was performed.

Cryo-transmission electron microscopy (Cryo-TEM). Cryo-TEM observations were performed on a Talos F200C G2 (ThermoFisher Scientific, USA) with an accelerating voltage of 200 kV. Cryo-TEM samples were obtained by dropping solutions of GNR-based nanoassemblies (500 μg/mL) onto a lacey carbon-film-supported TEM copper grid. A thin film of the sample solution was prepared via a Vitrobot Mark IV/Leica EM GP2 (ThermoFisher Scientific, USA) equipped with a humidity-controlled chamber. Following sample solution loading, the lacey carbon grid was blotted under preset parameters and immediately plunged into a liquid ethane reservoir precooled with liquid nitrogen. The vitrified samples were then transferred to a cryo-holder and cryo-transfer stage, both cooled with liquid nitrogen. To prevent sublimation of vitreous water, the cryo-holder temperature was kept below −170 °C throughout imaging.

Atomic force microscope (AFM). AFM visualization of GNR-based nanosheet-like assemblies were performed using a high-resolution multi-mode atomic force microscopy (Dimension XR, Bruker, USA) operated in tapping mode by using silicon nitride cantilevers with a force constant of 0.12 N/m.

3. Failed synthesis route for site-specific functionalized monomers

Failed synthesis route 1 (Anthracene-based monomer):

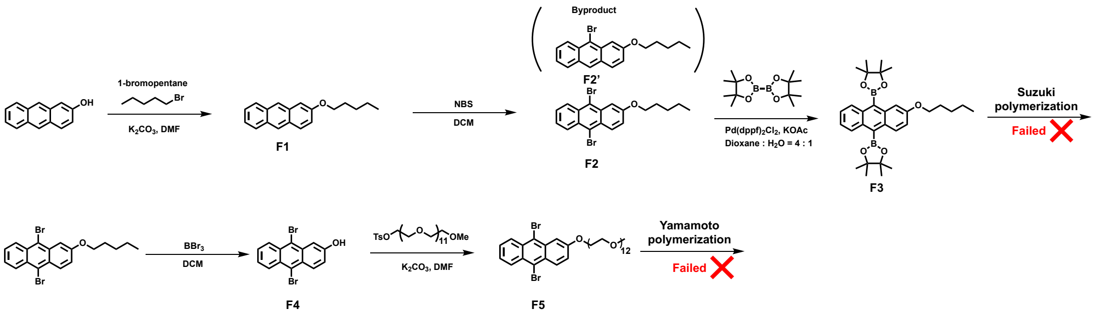

As shown in failed synthesis route 1, we initially attempted to synthesize anthracene-based monomer with site-specific functional RGs. However, the resultant anthracene-based monomer had low polymerization activity possibly due to the severe dehalogenation instead of polymerization. As a result, trace low-molecular-weight species was obtained even if the reaction temperature increased to 120 °C and the reaction time increased to 7 days. The ^{1}H NMR spectra of the corresponding compounds were shown below.

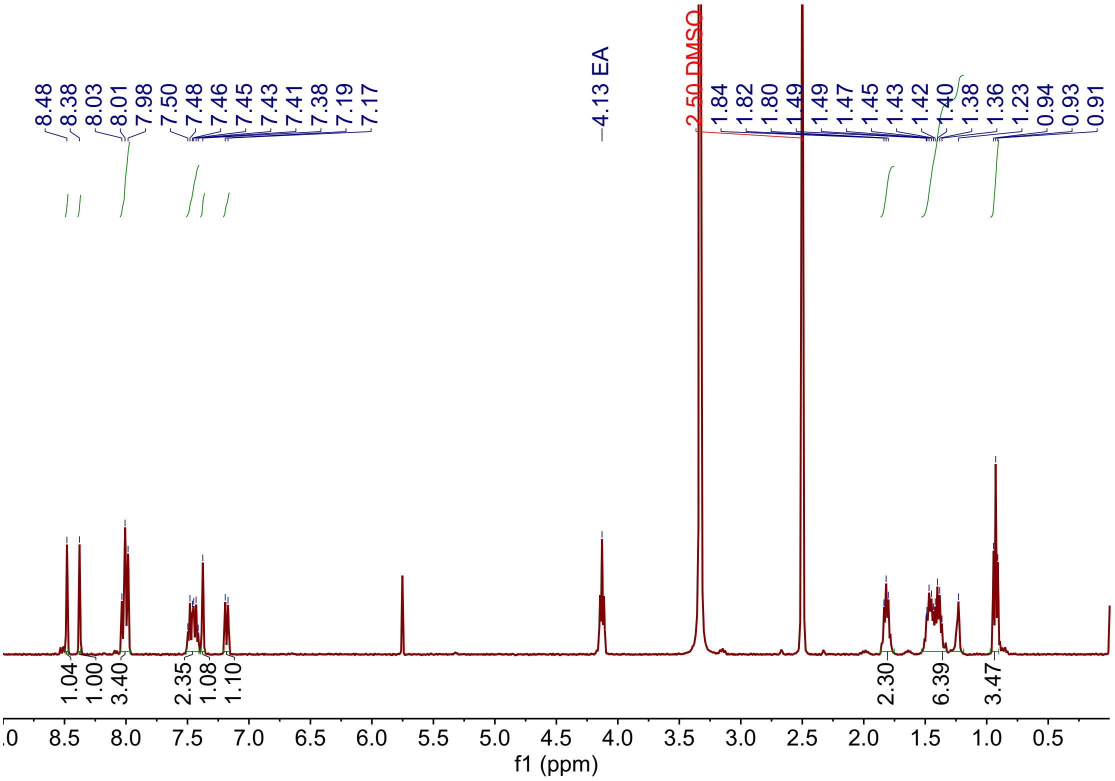

Supplementary Figure 1. ^{1}H NMR spectrum of **F1** in DMSO-d_6 (298 K).

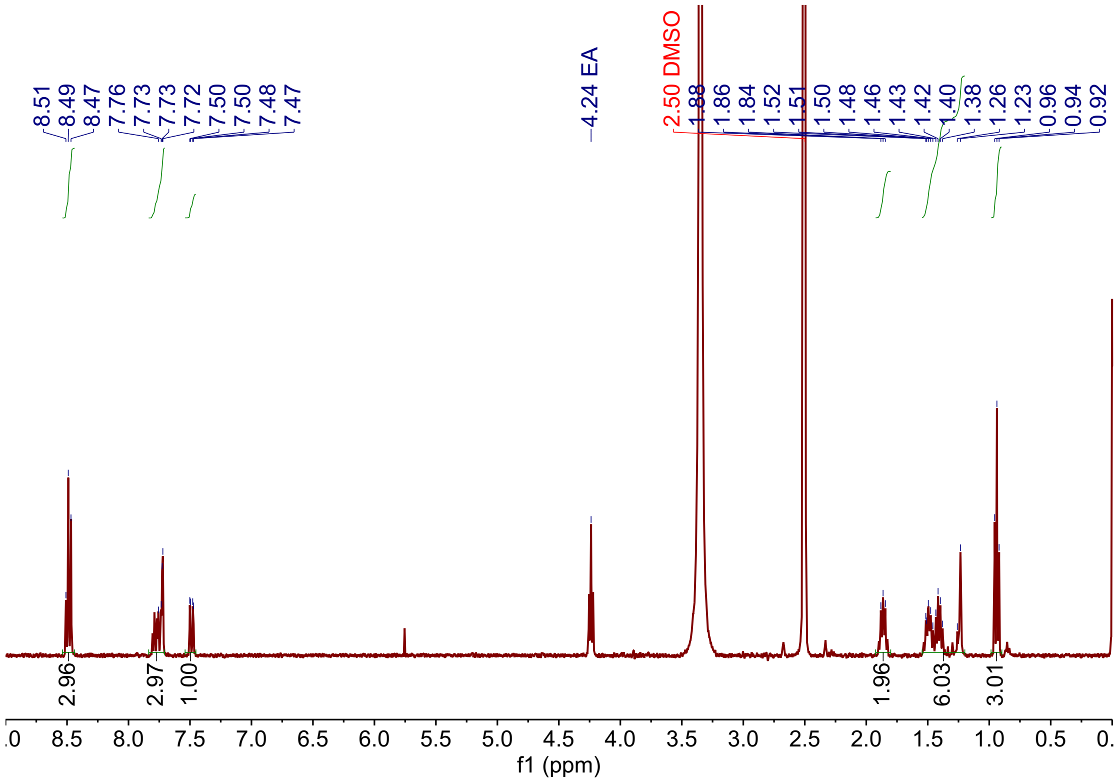

Supplementary Figure 2. ^{1}H NMR spectrum of **F2** in DMSO-d_6 (298 K).

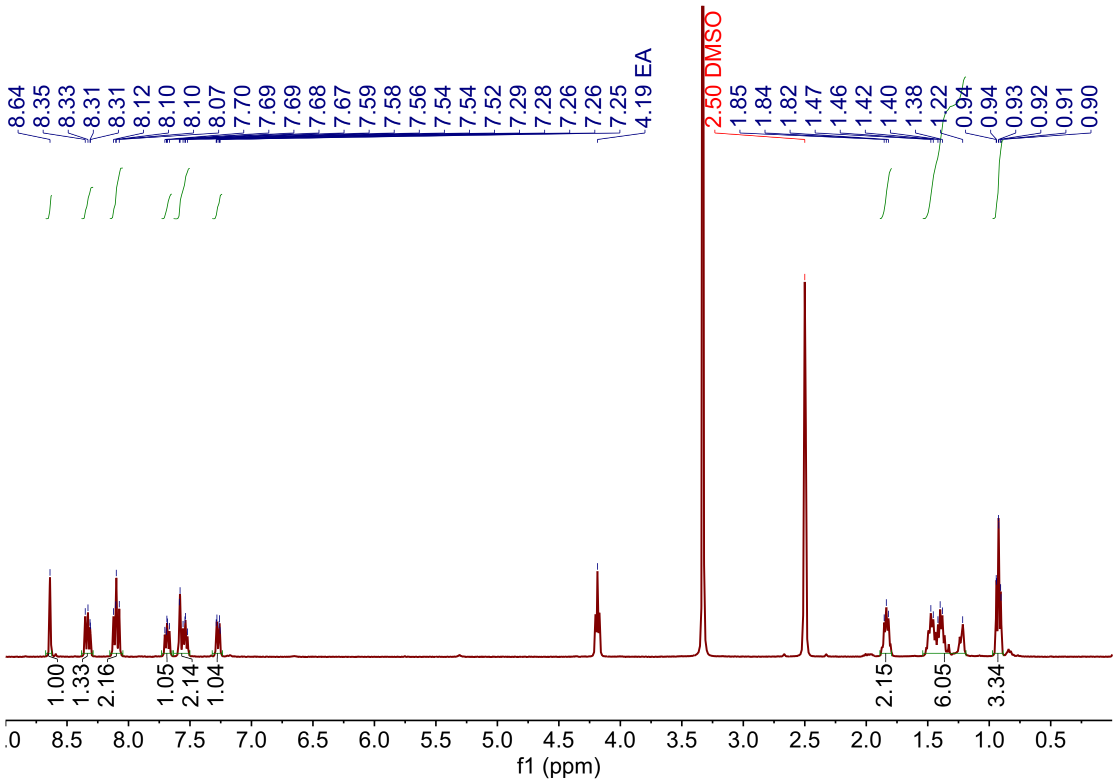

Supplementary Figure 3. ^{1}H NMR spectrum of **F2’** in DMSO-d_6 (298 K).

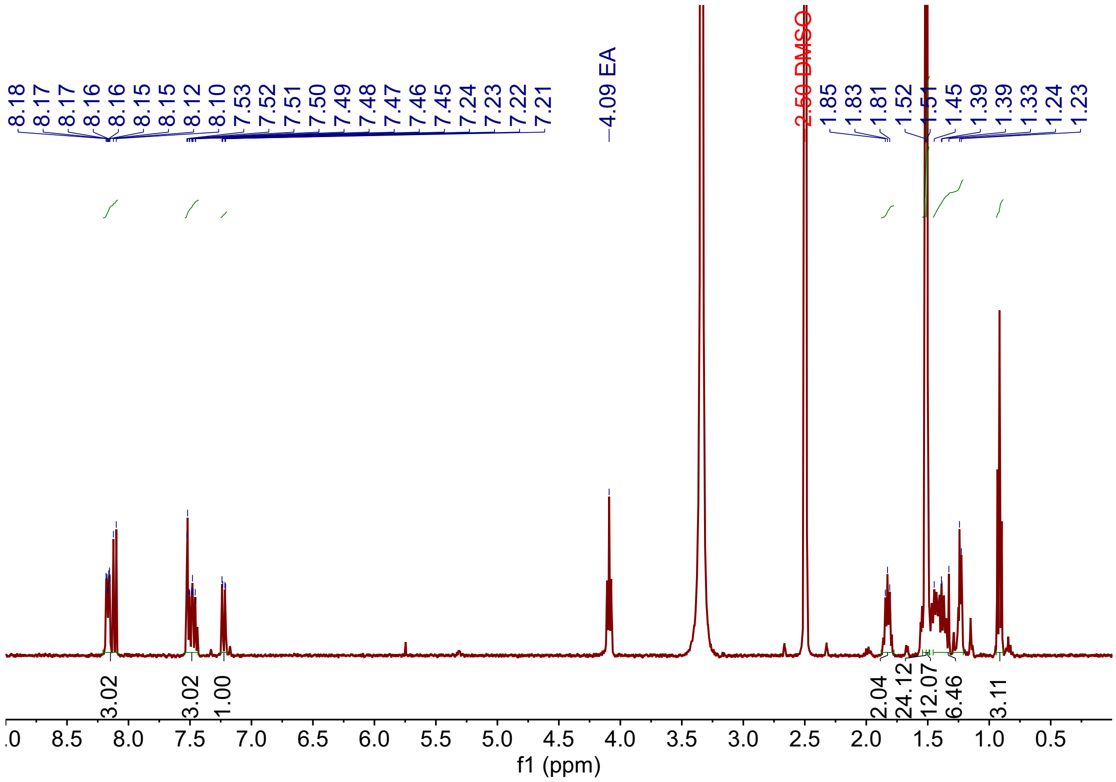

Supplementary Figure 4. ^{1}H NMR spectrum of **F3** in DMSO-d_6 (298 K).

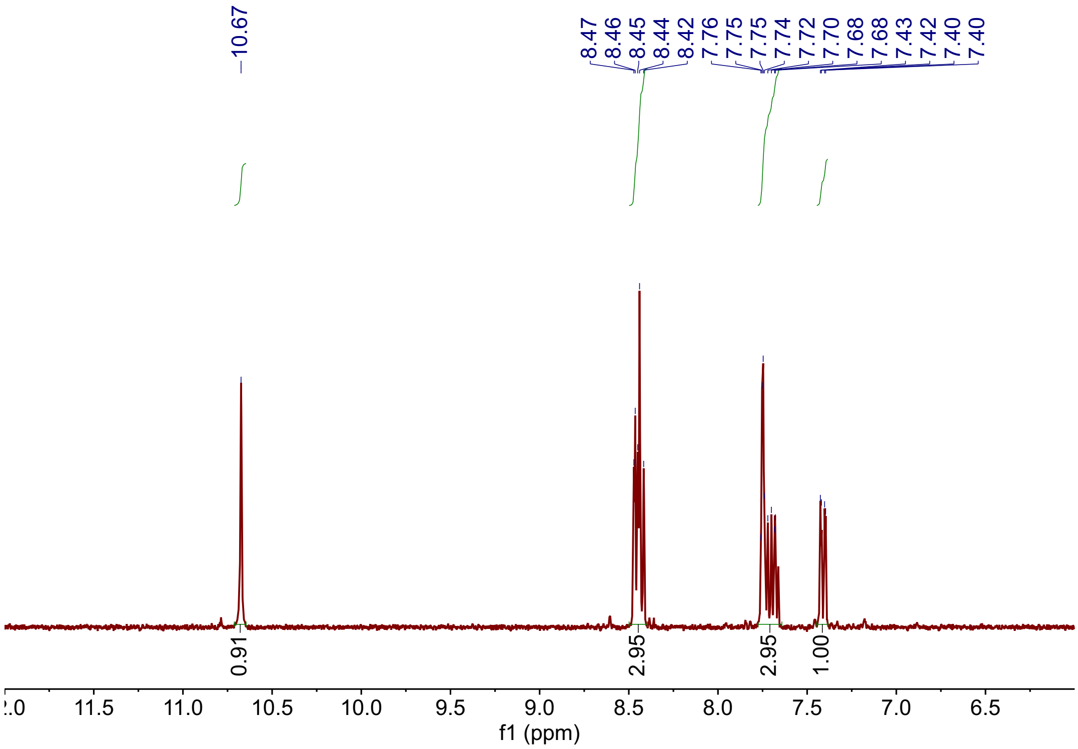

Supplementary Figure 5. ^{1}H NMR spectrum of **F4** in DMSO-d_6 (298 K).

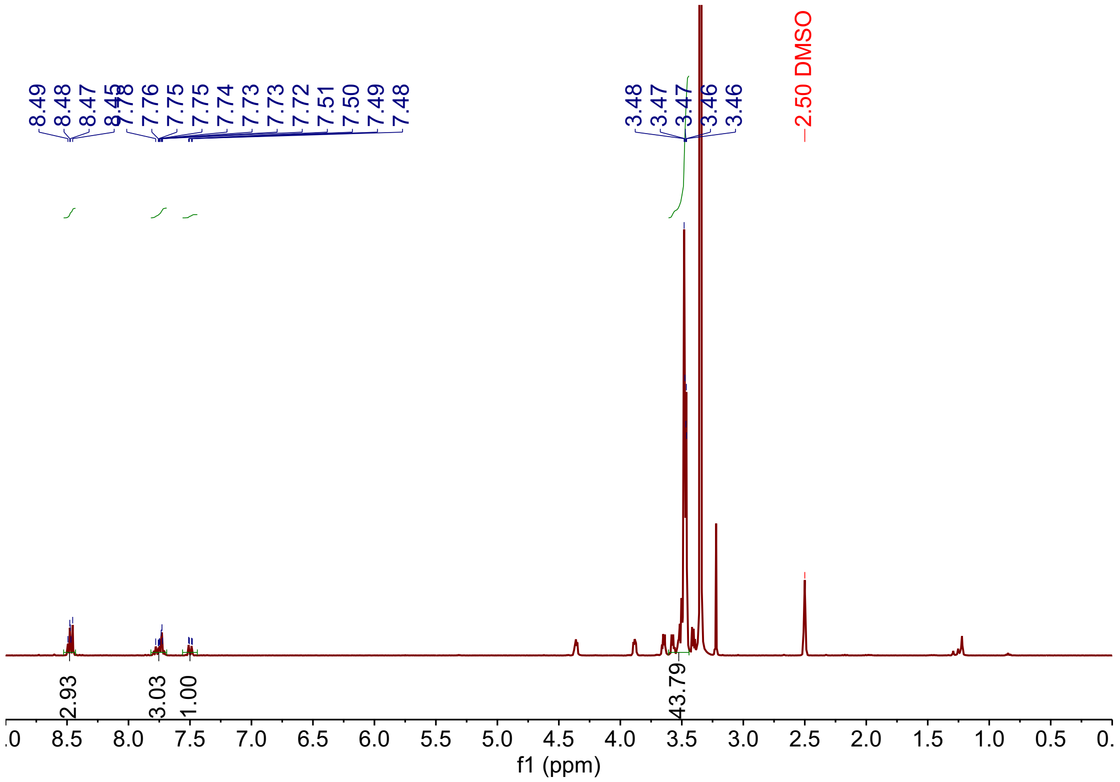

Supplementary Figure 6. ^{1}H NMR spectrum of **F5** in DMSO-d_6 (298 K).

Failed synthesis route 2 (Terphenyl-based monomers with ether linkage):

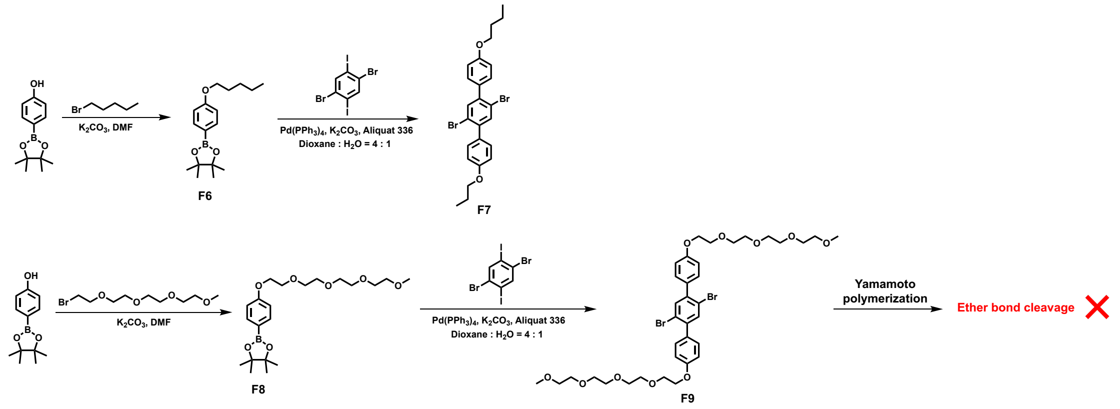

As shown in failed synthesis route 2, we attempted to synthesize terphenyl-based monomer with the ether linkage. However, the ether bond was unstable and easily cleaved under the polymerization conditions. Consequently, the side-chains of the polymerized products were completely removed, making it impossible to achieve the control over the side-chain sequence. The ^{1}H NMR spectra and the MALDI-TOF-MS spectrum of the related compounds were shown below.

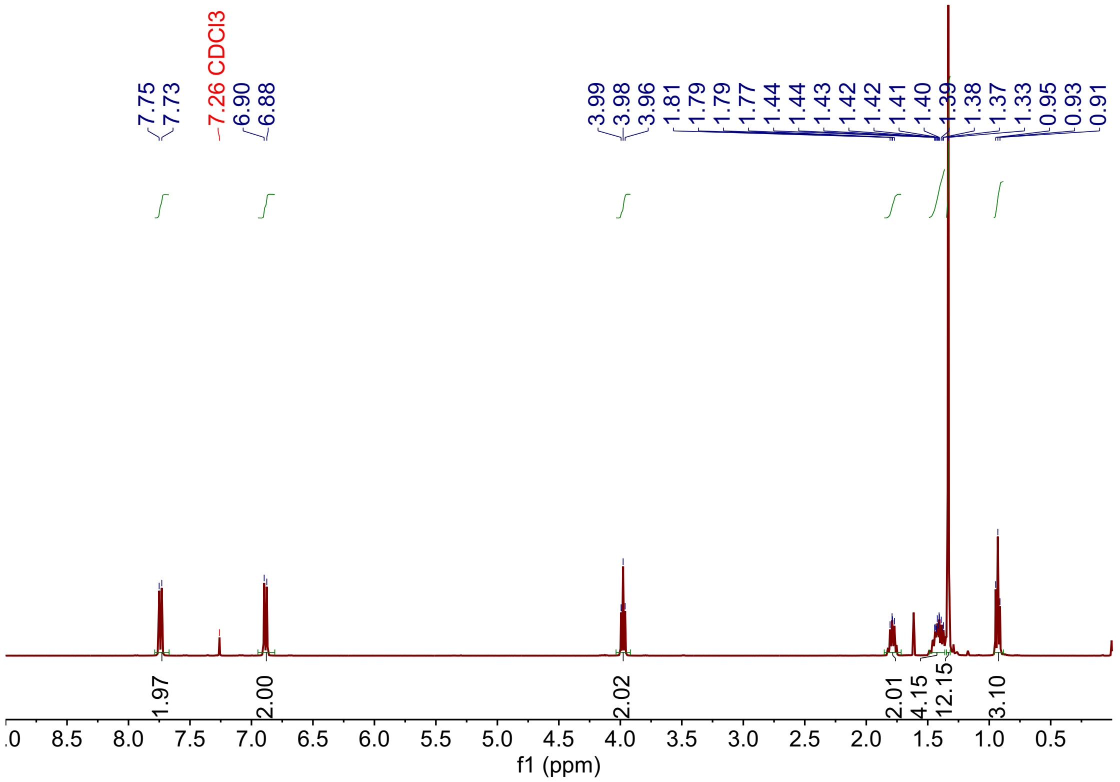

Supplementary Figure 7. ^{1}H NMR spectrum of **F6** in $CDCl_3$ (298 K).

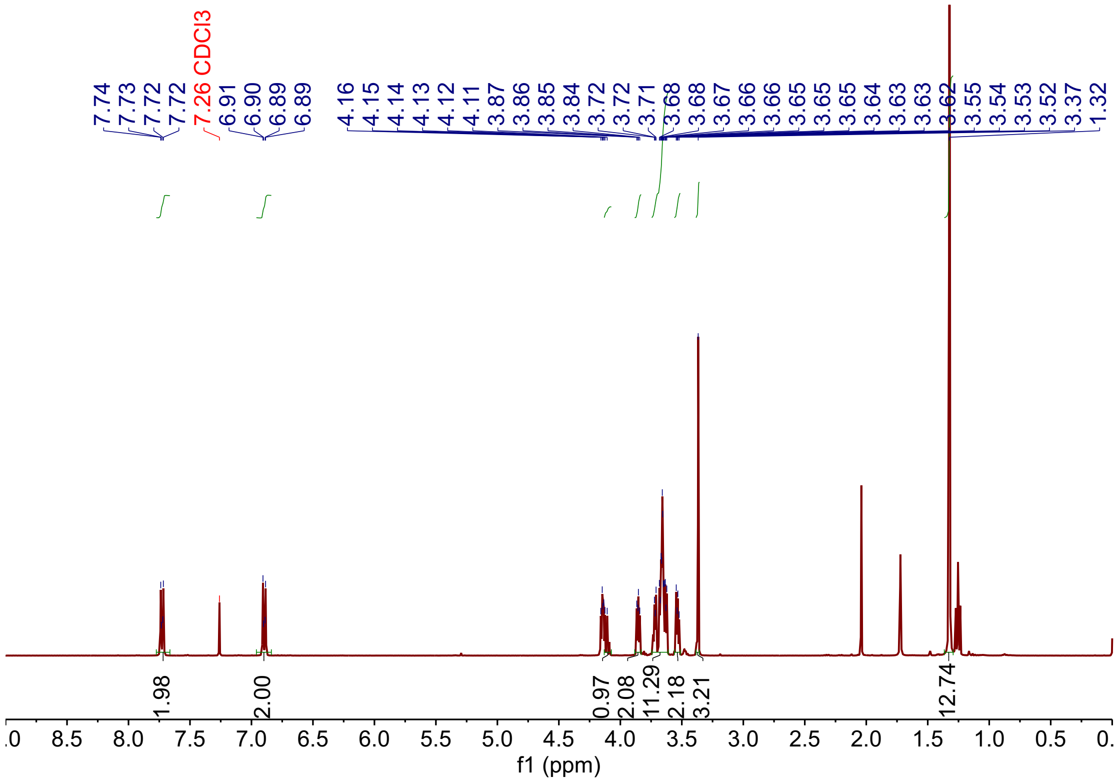

Supplementary Figure 8. ^{1}H NMR spectrum of **F8** in $CDCl_3$ (298 K).

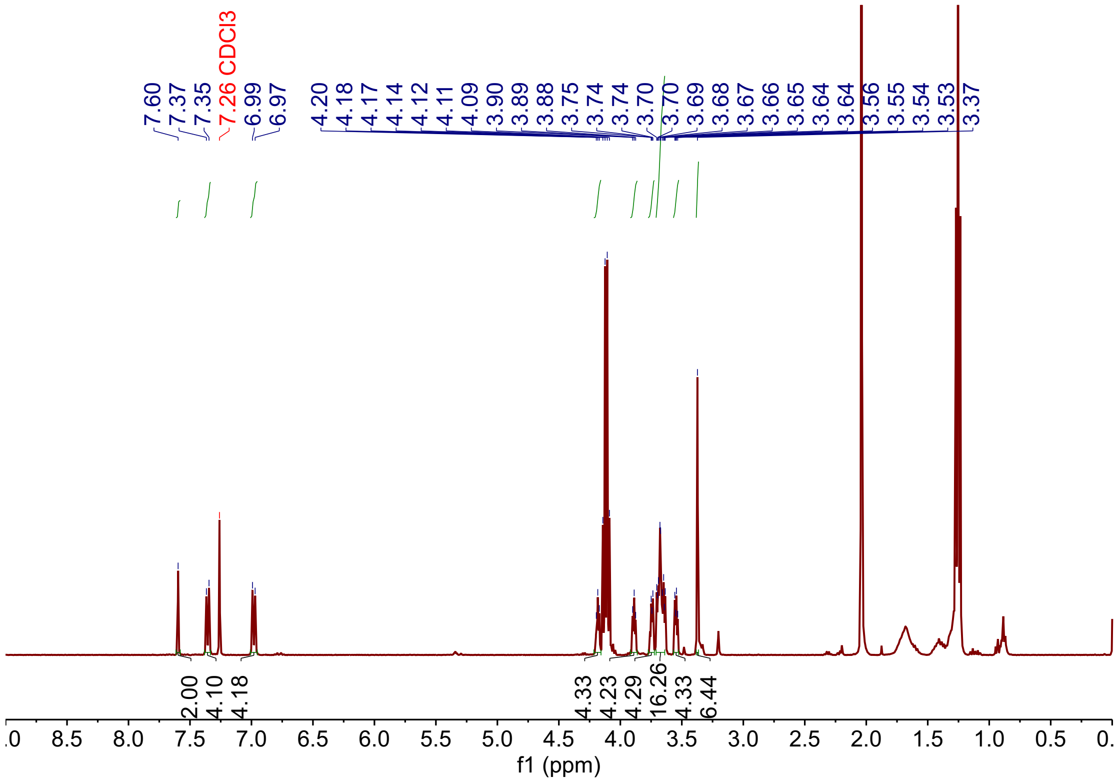

Supplementary Figure 9. ^{1}H NMR spectrum of **F9** in $CDCl_3$ (298 K).

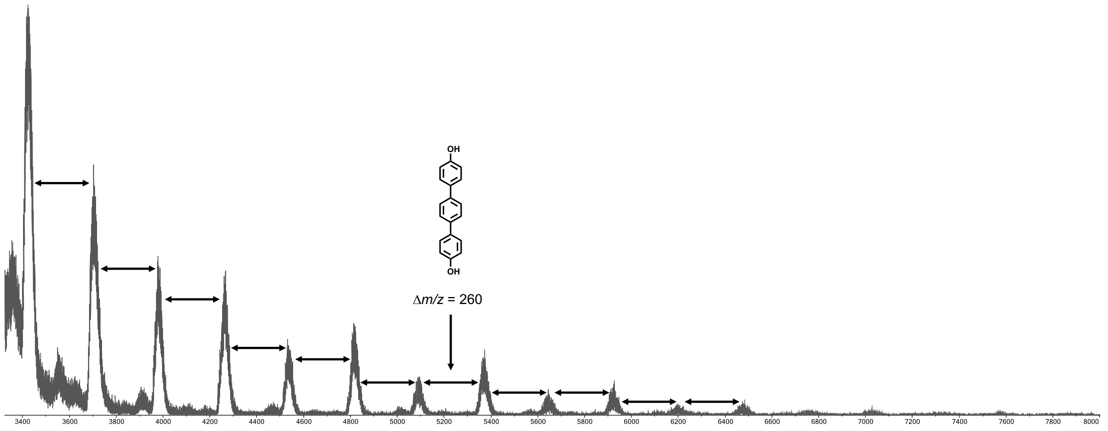

Supplementary Figure 10. MALDI-TOF-MS spectrum of the resulting polymerized products with the cleavage of the ether linkage.

4. Synthesis of terphenyl-based monomers with site-specific functional RGs

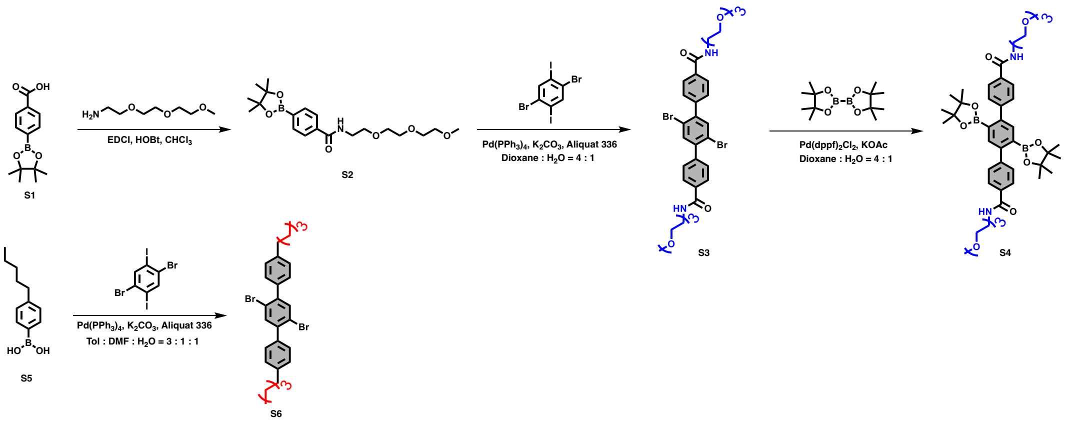

***N*-(2-(2-(2-methoxyethoxy)ethoxy)ethyl)-4-(4,4,5,5-tetramethyl-1,3,2-dioxaborolan-2-yl)benzamide, S2**

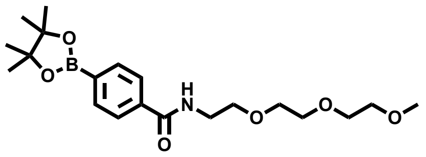

To an ice-cooled solution of 4-(4,4,5,5-tetramethyl-1,3,2-dioxaborolan-2-yl)benzoic acid (**S1**, 1.0 g, 4.03 mmol, 1.0 equiv.), EDCI (1.16 g, 6.05 mmol, 1.5 equiv.) in $CHCl_3$ (30 mL) was added HOBt (0.27 g, 2.02 mmol, 0.5 equiv.). The mixture was stirred at 0 °C for 10 min, followed by the addition of 2-(2-(2-methoxyethoxy)ethoxy)ethan-1-amine (0.99 g, 6.05 mmol, 1.5 equiv.). The ice bath was then removed and the resulting solution was stirred at room temperature for 3 h. The mixture was diluted with $CHCl_3$ (100 mL) then washed with half-sat. $NaHCO_3$ (50 mL × 3) and dried with Na_2SO_4. After removing the solvent, the resulting crude was purified by silica gel chromatography with a gradient of EtOAc-heptane (5/95 to 70/30, v/v). The eluent was concentrated in vacuo to give the target product (**S2**, 1.15 g, 73%) as a colourless oil.

R_f (EtOAc) = 0.4

^{1}H NMR (400 MHz, $CDCl_3$) δ = 7.86 (d, J = 8.2 Hz, 2H), 7.78 (d, J = 8.2 Hz, 1H), 3.72 – 3.60 (m, 10H), 3.57 – 3.48 (m, 2H), 3.32 (s, 3H), 1.34 (s, 12H).

^{13}C NMR (100 MHz, $CDCl_3$) δ = 167.55, 136.96, 134.96, 126.29, 84.20, 72.00, 70.64, 70.61, 70.36, 69.92, 59.10, 39.87, 24.99.

***m/z* (MALDI-TOF$^+$)** $[M+Na]^+$ = 416.23, calc. $C_{20}H_{32}BNO_6Na^+$ = 416.22

2',5'-dibromo-*N*^{4},*N*$^{4''}$-bis(2-(2-(2-methoxyethoxy)ethoxy)ethyl)-[1,1':4',1''-terphenyl]-4,4''-dicarboxamide, S3

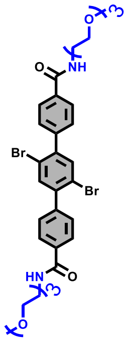

N-(2-(2-(2-methoxyethoxy)ethoxy)ethyl)-4-(4,4,5,5-tetramethyl-1,3,2-dioxaborolan-2-yl)benzamide (**S2**, 1.15 g, 2.92 mmol, 2.3 equiv.), 1,4-dibromo-2,5-diiodobenzene (620 mg, 1.27 mmol, 1.0 equiv.), $Pd(PPh_3)_4$ (147 mg, 0.127 mmol, 0.1 equiv.), K_2CO_3 (877 mg, 6.35 mmol, 5.0 equiv.) and few drops Aliquat 336 were placed in a Schlenk flask, and the flask was pump-filled with N_2 three times. Then a degassed mixture of dioxane (24 mL) and H_2O (6 mL) was added. The flask was sealed tightly and then heated to 80 °C for 24 h. After being cooled down to room temperature, the solvent was removed in vacuo and the residue was re-dissolved in CH_2Cl_2 (100 mL) and extracted with half-sat. brine (30 mL × 3). The organic phase was dried over anhydrous Na_2SO_4 and concentrated in vacuo. The resulting crude was purified by silica gel chromatography with a gradient of MeOH-EtOAc (1/99 to 5/95, v/v). The eluent was concentrated in vacuo to give the target product (**S3**, 0.83 g, 86%) as a yellow solid.

R_f (MeOH : EtOAc = 1 : 9) = 0.55

^{1}H NMR (400 MHz, $CDCl_3$) δ = 7.91 (d, J = 8.2 Hz, 4H), 7.64 (s, 2H), 7.51 (d, J = 8.3 Hz, 4H), 7.02 (br, 2H), 3.76 – 3.63 (m, 20H), 3.60 – 3.52 (m, 4H), 3.35 (s, 6H).

^{13}C NMR (100 MHz, $CDCl_3$) δ = 167.13, 142.60, 135.28, 134.53, 129.59, 127.19, 121.38, 72.06, 70.64, 70.40, 69.98, 59.16, 39.97.

***m/z* (MALDI-TOF$^+$)** $[M+H]^+$ = 767.13, calc. $C_{34}H_{43}Br_2N_2O_8^+$ = 767.13

***N*[4],*N*[4''] -bis(2-(2-(2-methoxyethoxy)ethoxy)ethyl)-2',5'-bis(4,4,5,5-tetramethyl-1,3,2-dioxaborolan-2-yl)-[1,1':4',1''-terphenyl]-4,4''-dicarboxamide, S4**

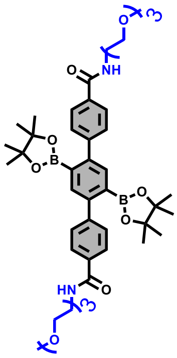

2',5'-dibromo-N^4,$N^{4''}$-bis(2-(2-(2-methoxyethoxy)ethoxy)ethyl)-[1,1':4',1''-terphenyl]-4,4''-dicarboxamide (S3, 0.28 g, 0.37 mmol, 1.0 equiv.), bis(pinacolato)diboron (0.38 g, 1.48 mmol, 4.0 equiv.), Pd(dppf)$_2$Cl$_2$ (54 mg, 0.074 mmol, 0.2 equiv.) and KOAc (362 mg, 3.69 mmol, 10.0 equiv.) were placed in a Schlenk flask, and the flask was pump-filled with N_2 three times. Then a degassed mixture of dioxane (8 mL) and H_2O (2 mL) was added. The flask was sealed tightly and then heated to 80 °C for 48 h. After being cooled down to room temperature, the solvent was removed in vacuo and the residue was re-dissolved in CH_2Cl_2 (100 mL) and extracted with half-sat. brine (30 mL × 3). The organic phase was dried over anhydrous Na_2SO_4 and concentrated in vacuo. The resulting crude was purified by silica gel chromatography with a gradient of MeOH-EtOAc (1/99 to 5/95, v/v). The eluent was concentrated in vacuo to give the target product (**S4**, 99 mg, 31.2%) as a yellow solid.

R_f (MeOH : EtOAc = 1 : 9) = 0.5

^{1}H NMR (400 MHz, CDCl$_3$) δ = 7.84 (d, J = 8.2 Hz, 4H), 7.72 (s, 2H), 7.50 (d, J = 8.3 Hz, 4H), 6.90 (br, 2H), 3.73 – 3.65 (m, 20H), 3.59 – 3.52 (m, 4H), 3.36 (s, 6H), 1.20 (s, 24H).

^{13}C NMR (100 MHz, CDCl$_3$) δ = 167.16, 136.18, 135.27, 133.64, 129.59, 127.20, 123.83, 121.38, 84.18, 72.04, 70.67, 70.65, 70.39, 59.17, 51.04, 39.97, 24.73.

***m/z* (MALDI-TOF$^+$)** [M+H]$^+$ = 861.48, calc. $C_{46}H_{67}B_2N_2O_{12}^+$ = 861.48

2',5'-dibromo-4,4''-dipentyl-1,1':4',1''-terphenyl, S6

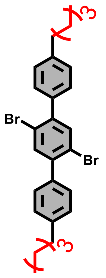

(4-pentylphenyl)boronic acid (1.0 g, 5.2 mmol, 2.3 equiv.), 1,4-dibromo-2,5-diiodobenzene (1.1 g, 2.26 mmol, 1.0 equiv.), $Pd(PPh_3)_4$ (265 mg, 0.23 mmol, 0.1 equiv.), K_2CO_3 (1.56 g, 11.3 mmol, 5.0 equiv.) and few drops Aliquat 336 were placed in a Schlenk flask, and the flask was pump-filled with N_2 three times. Then a degassed mixture of dioxane (24 mL) and H_2O (6 mL) was added. The flask was sealed tightly and then heated to 80 °C for 24 h. After being cooled down to room temperature, the solvent was removed in vacuo and the residue was re-dissolved in CH_2Cl_2 (100 mL) and extracted with half-sat. brine (30 mL × 3). The organic phase was dried over anhydrous Na_2SO_4 and concentrated in vacuo. The resulting crude was purified by a flash column with PE. The eluent was concentrated in vacuo to give the target product (**S6**, 1.45 g, 52.7%) as a white solid.

R_f (PE) = 0.6

^{1}H NMR (400 MHz, $CDCl_3$) δ = 7.63 (s, 2H), 7.36 (d, J = 8.1 Hz, 4H), 7.26 (d, J = 8.3 Hz, 4H), 2.66 (dd, J = 8.9, 6.7 Hz, 4H), 1.73 – 1.62 (m, 4H), 1.40 – 1.33 (m, 8H), 0.95 – 0.88 (m, 6H).

^{13}C NMR (100 MHz, $CDCl_3$) δ = 142.83, 136.91, 135.39, 129.29, 128.30, 121.51, 35.88, 31.75, 31.20, 22.71, 14.20.

***m/z* (MALDI-TOF$^+$)** $[M+H]^+$ = 528.08, calc. $C_{28}H_{33}Br_2^+$ = 528.09

5. Synthesis of sequence-regulated graphene nanoribbon, A_n-GNR

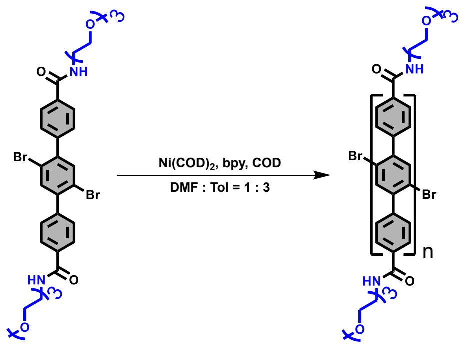

Synthesis of A_n-PT

A mixture of 2',5'-dibromo-N^4,$N^{4''}$-bis(2-(2-(2-methoxyethoxy)ethoxy)ethyl)-[1,1':4',1''-terphenyl]-4,4''-dicarboxamide (**S3**, 100 mg, 0.13 mmol, 1.0 equiv.), $Ni(COD)_2$ (179 mg, 0.65 mmol, 5.0 equiv.), cyclooctadiene (70 mg, 0.65 mmol, 5.0 equiv.), and 2,2'-bipyridine (102 mg, 0.65 mmol, 5.0 equiv.) were dissolved into the mixed solution of DMF (0.50 mL) and toluene (1.5 mL). After being stirred at 60 °C for 30 min, the resulting mixture was stirred at 80 °C for 48 h in dark. Then the reaction mixture was cooled down to room temperature and dropwise added into diethyl ether, yielding dark grey precipitate. The precipitate was collected by filtration, followed by vacuum drying, giving rise to a dark grey solid product (56 mg, 56 % yield).

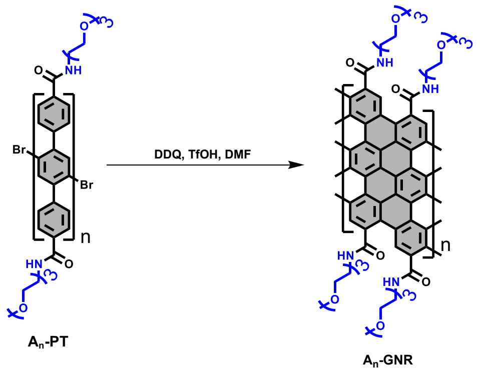

A_n-GNR synthesis via cyclodehydrogenation

To a solution of A_n-PT (23 mg, 4.6 μmol, 1.0 equiv.) and DDQ (16 mg, 69 μmol, 15.0 equiv.) dissolved in dry degassed DMF (5 mL) was added triflic acid (0.25 mL). The reaction mixture was stirred at room temperature overnight, then triethylamine (0.5 mL) was added via a syringe

to quench the reaction. After most DMF being removed under high vacuum, diethyl ether (1 mL) and the precipitate was collected by filtration to afford tittle compound **A_n-GNR** (20 mg, 87% yield) as dark brown solid.

6. Synthesis of sequence-regulated graphene nanoribbon, (AB)$_n$-GNR

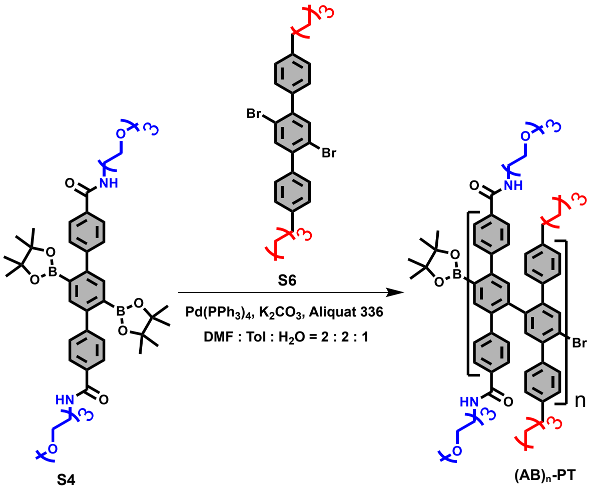

Synthesis of (AB)$_n$-PT

2',5'-dibromo-N^4,$N^{4''}$-bis(2-(2-(2-methoxyethoxy)ethoxy)ethyl)-[1,1':4',1''-terphenyl]-4,4''-dicarboxamide (**S3**, 50 mg, 0.058 mmol, 1.0 equiv.), 2',5'-dibromo-4,4''-dipentyl-1,1':4',1''-terphenyl (**S6**, 30 mg, 0.058 mmol, 1.0 equiv.), $Pd(PPh_3)_4$ (27 mg, 0.023 mmol, 0.4 equiv.), K_2CO_3 (160 mg, 1.16 mmol, 20.0 equiv.) and few drops Aliquat 336 were placed in a Schlenk flask, and the flask was pump-filled with N_2 three times. Then a degassed mixture of DMF (0.8 mL), toluene (0.8 mL) and H_2O (0.4 mL) were added. The flask was sealed tightly and then heated to 80 °C for 48 h. After being cooled down to room temperature, the reaction mixture was dropwise added into diethyl ether, yielding dark brown precipitate. The precipitate was collected by filtration, followed by vacuum drying, giving rise to dark brown solid product (31 mg, 39 % yield).

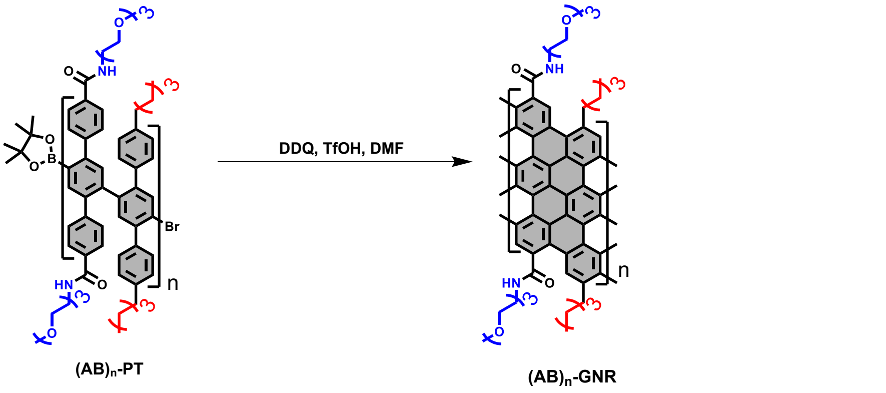

(AB)$_n$-GNR synthesis via cyclodehydrogenation

To a solution of (AB)$_n$-PT (25 mg, 5.6 μmol, 1.0 equiv.) and DDQ (19 mg, 83 μmol, 15.0 equiv.) dissolved in dry degassed DMF (5 mL) was added triflic acid (0.25 mL). The reaction mixture was stirred at room temperature overnight, then triethylamine (0.5 mL) was added via a syringe to quench the reaction. After most DMF being removed under high vacuum, diethyl ether (1 mL) and the precipitate was collected by filtration to afford tittle compound **(AB)$_n$-GNR** (19 mg, 76% yield) as dark brown solid.

7. Synthesis of sequence-regulated graphene nanoribbon, B$_m$A$_n$B$_m$-GNR

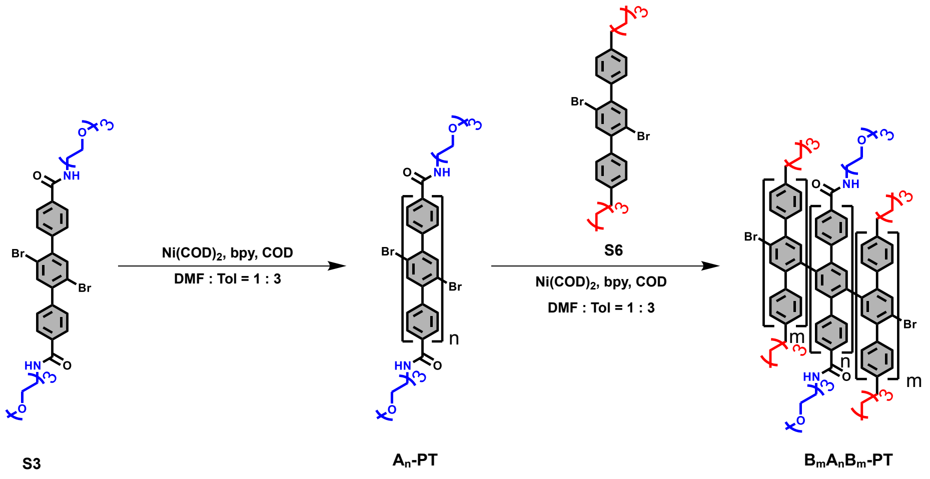

Synthesis of B$_m$A$_n$B$_m$-PT

A mixture of 2',5'-dibromo-N^4,$N^{4''}$-bis(2-(2-(2-methoxyethoxy)ethoxy)ethyl)-[1,1':4',1''-terphenyl]-4,4''-dicarboxamide (**S3**, 50 mg, 0.065 mmol, 1.0 equiv.), Ni(COD)$_2$ (179 mg, 0.65 mmol, 10.0 equiv.), cyclooctadiene (70 mg, 0.65 mmol, 10.0 equiv.), and 2,2'-bipyridine (102

mg, 0.65 mmol, 10.0 equiv.) were dissolved into the mixed solution of DMF (0.50 ml) and toluene (1.5 ml). After being stirred at 60 °C for 30 min, the resulting mixture was stirred at 80 °C for another 12 h in dark. Then, 2',5'-dibromo-4,4''-dipentyl-1,1':4',1''-terphenyl (**S6**, 34.5 mg, 0.065 mmol, 1.0 equiv.) was added to the reaction mixture under N_2 atmosphere. Subsequently, the reaction mixture was stirred at 80 °C for 36 h in dark. Then the reaction mixture was cooled down to room temperature and dropwise added into hexane, yielding dark brown precipitate. The precipitate was collected by filtration, followed by vacuum drying, giving rise to an off-white solid product (43 mg, 51 % yield).

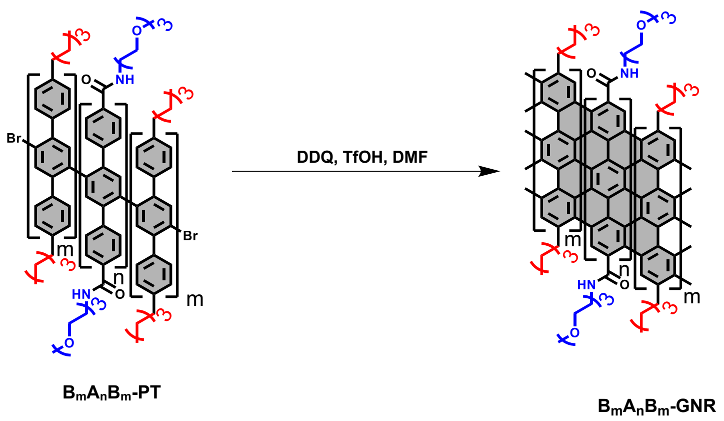

$B_mA_nB_m$-GNR synthesis via cyclodehydrogenation

To a solution of $B_mA_nB_m$-PT (9 mg, 1.7 μmol, 1.0 equiv.) and DDQ (6 mg, 26 μmol, 15.0 equiv.) dissolved in dry degassed DMF (2 mL) was added triflic acid (0.1 mL). The reaction mixture was stirred at room temperature overnight, then triethylamine (0.2 mL) was added via a syringe to quench the reaction. After most DMF being removed under high vacuum, hexane (0.5 mL) and the precipitate was collected by filtration to afford tittle compound **$B_mA_nB_m$-GNR** (5 mg, 56% yield) as dark brown solid.

8. Synthesis of sequence-regulated graphene nanoribbon, $(A_xB_{1-x})_n$-GNR

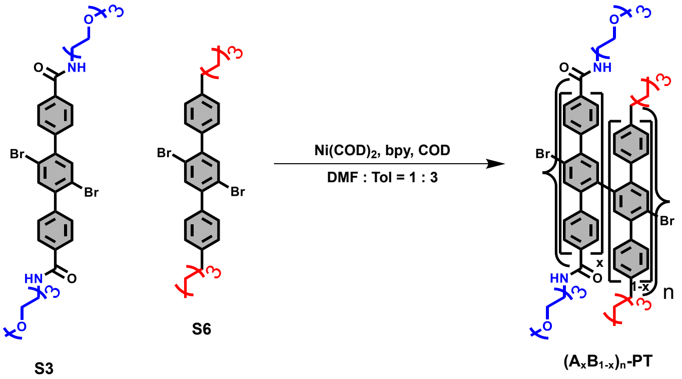

Synthesis of $(A_xB_{1-x})_n$-PT

A mixture of 2',5'-dibromo-*N*[4],*N*[4"]-bis(2-(2-(2-methoxyethoxy)ethoxy)ethyl)-[1,1':4',1"-terphenyl]-4,4"-dicarboxamide (**S3**, 50 mg, 0.065 mmol, 1.0 equiv.), 2',5'-dibromo-4,4"-dipentyl-1,1':4',1"-terphenyl (**S6**, 34.5 mg, 0.065 mmol, 1.0 equiv.), $Ni(COD)_2$ (179 mg, 0.65 mmol, 10.0 equiv.), cyclooctadiene (70 mg, 0.65 mmol, 10.0 equiv.), and 2,2'-bipyridine (102 mg, 0.65 mmol, 10.0 equiv.) were dissolved into the mixed solution of DMF (0.50 ml) and toluene (1.5 ml). After being stirred at 60 °C for 30 min, the resulting mixture was stirred at 80 °C for 48 h in dark. After reaction, the reaction mixture was cooled down to room temperature and dropwise added into diethyl ether, yielding dark brown precipitate. The precipitate was collected by filtration, followed by vacuum drying, giving rise to a dark brown solid product (56 mg, 56 % yield).

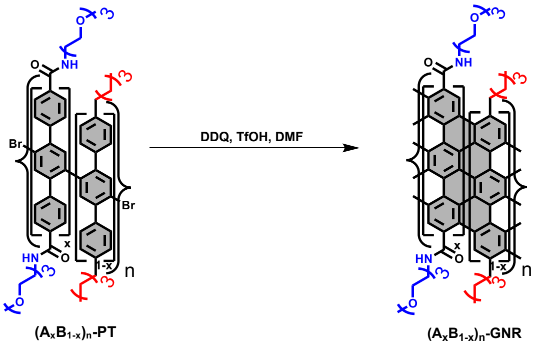

$(A_xB_{1-x})_n$-GNR synthesis via cyclodehydrogenation

To a solution of $(A_xB_{1-x})_n$-PT (7 mg, 1.5 μmol, 1.0 equiv.) and DDQ (5 mg, 22 μmol, 15.0 equiv.) dissolved in dry degassed DMF (2 mL) was added triflic acid (0.1 mL). The reaction mixture

was stirred at room temperature overnight, then triethylamine (0.2 mL) was added via a syringe to quench the reaction. After most DMF being removed under high vacuum, hexane (0.5 mL) and the precipitate was collected by filtration to afford tittle compound **$(A_xB_{1-x})_n$-GNR** (5 mg, 71% yield) as dark brown solid.

9. Supplementary SEC spectra

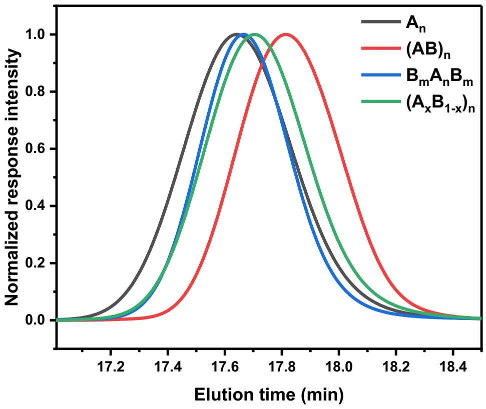

Side-chian sequenced GNRs	M_n (g/mol)	PDI
A_n-GNR	5300	1.10
$(AB)_n$-GNR	4700	1.08
$B_mA_nB_m$-GNR	5200	1.04
$(A_xB_{1-x})_n$-GNR	4900	1.06

Supplementary Figure 11. SEC traces (DMF eluent) of A_n-PT, $(AB)_n$-PT, $B_mA_nB_m$-PT and $(A_xB_{1-x})_n$-PT, with number-average molecular weights (M_n) and polydispersity indices (PDI) determined by SEC.

10. Supplementary UV-vis and PL spectra

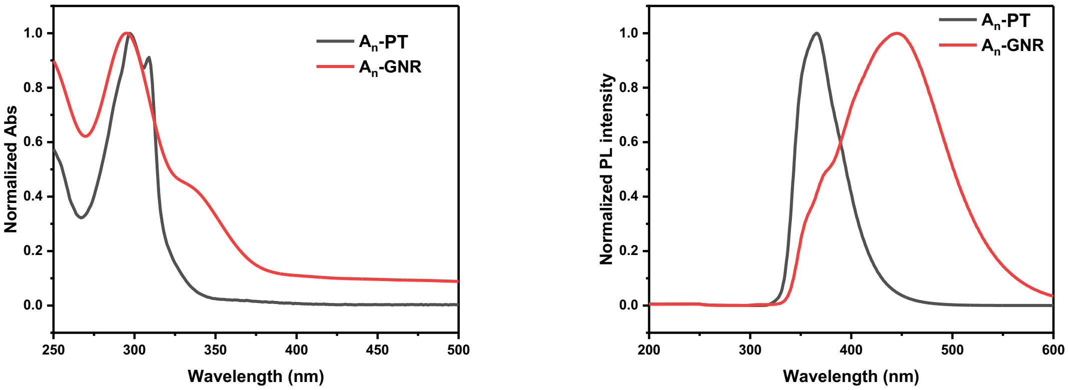

Supplementary Figure 12. UV-vis absorption spectra (left) and steady-state fluorescence spectra (right) of A_n-PT and A_n-GNR in MeOH.

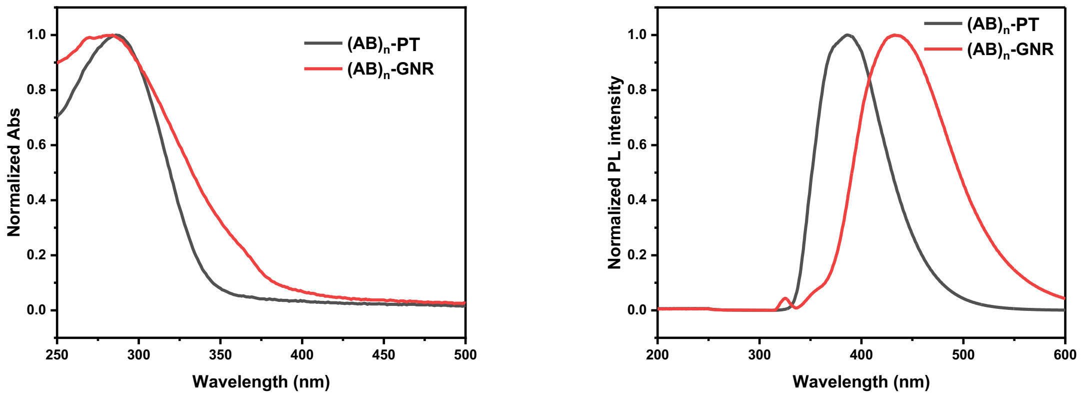

Supplementary Figure 13. UV-vis absorption spectra (left) and steady-state fluorescence spectra (right) of $(AB)_n$-PT and $(AB)_n$-GNR in MeOH.

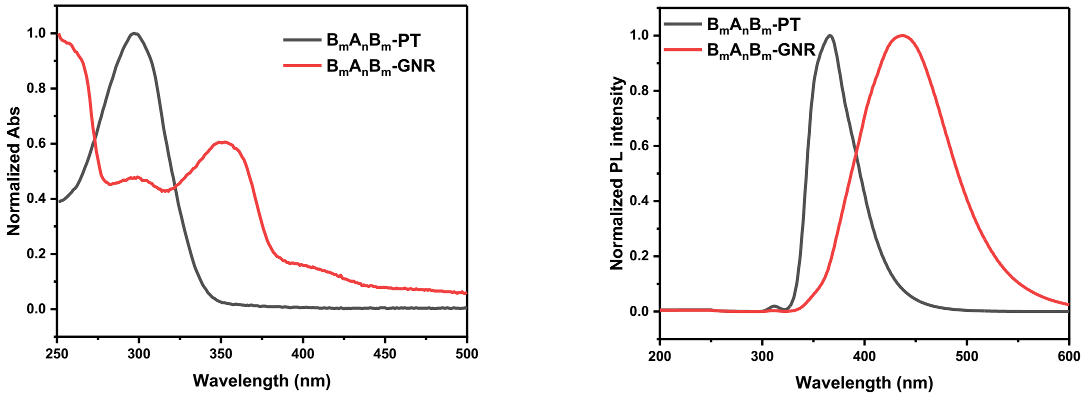

Supplementary Figure 14. UV-vis absorption spectra (left) and steady-state fluorescence spectra (right) of $B_mA_nB_m$-PT and $B_mA_nB_m$-GNR in MeOH.

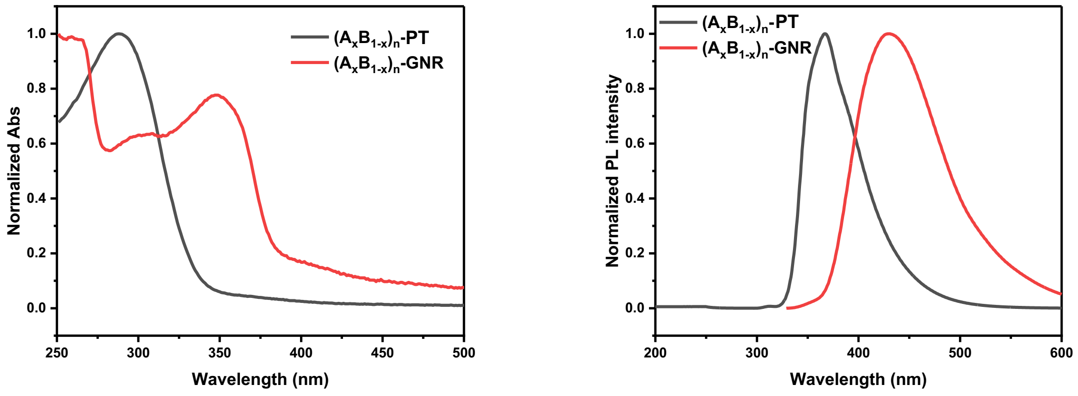

Supplementary Figure 15. UV-vis absorption spectra (left) and steady-state fluorescence spectra (right) of $(A_xB_{1-x})_n$-PT and $(A_xB_{1-x})_n$-GNR in MeOH.

11. Supplementary FTIR spectra

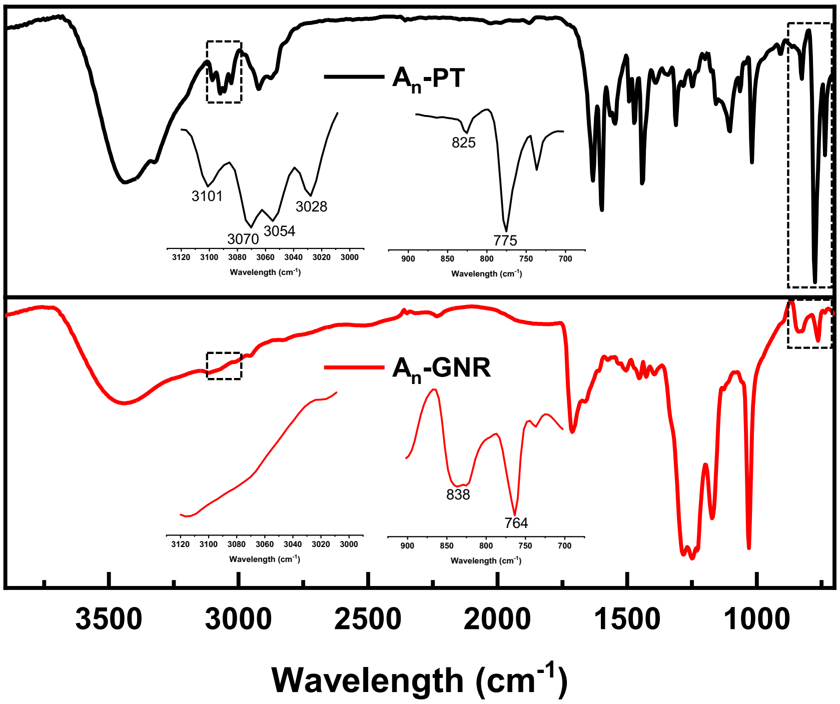

Supplementary Figure 16. FTIR spectra of A_n-PT and A_n-GNR after cyclodehydrogenation. The evident attenuation of the peaks from aromatic C–H stretching vibrations at 3101, 3070, 3054 and 3028 cm^{-1} in the spectrum of A_n-GNR, the disappearance of bands at 825 and 775 cm^{-1} that attributed to mono- and di-substituted benzene rings, and the appearance of bands for aromatic C–H at the edge positions at 838 and 764 cm^{-1} confirm the cyclodehydrogenation of the precursor polyterphenyls[1-2].

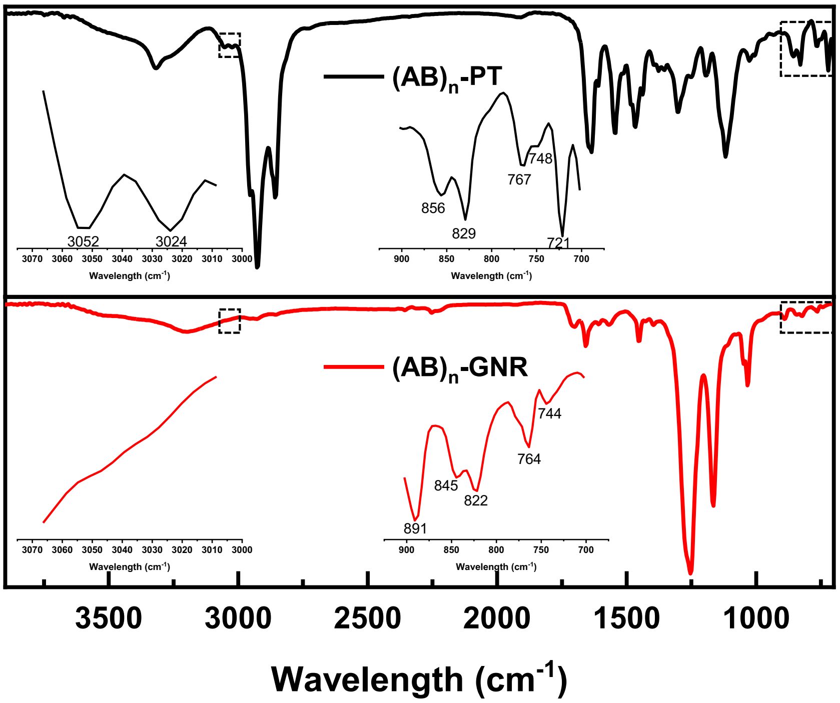

Supplementary Figure 17. FTIR spectra of $(AB)_n$-PT and $(AB)_n$-GNR after cyclodehydrogenation. The evident attenuation of the peaks from aromatic C–H stretching vibrations at 3052 and 3024 cm^{-1} in spectrum of $(AB)_n$-GNR, the disappearance of bands at 856, 829 and 721 cm^{-1} that attributed to mono- and di-substituted benzene rings, and the appearance of bands for aromatic C–H at the edge positions at 891, 845 and 822 cm^{-1} confirm the cyclodehydrogenation of the precursor polyterphenyls[1-2].

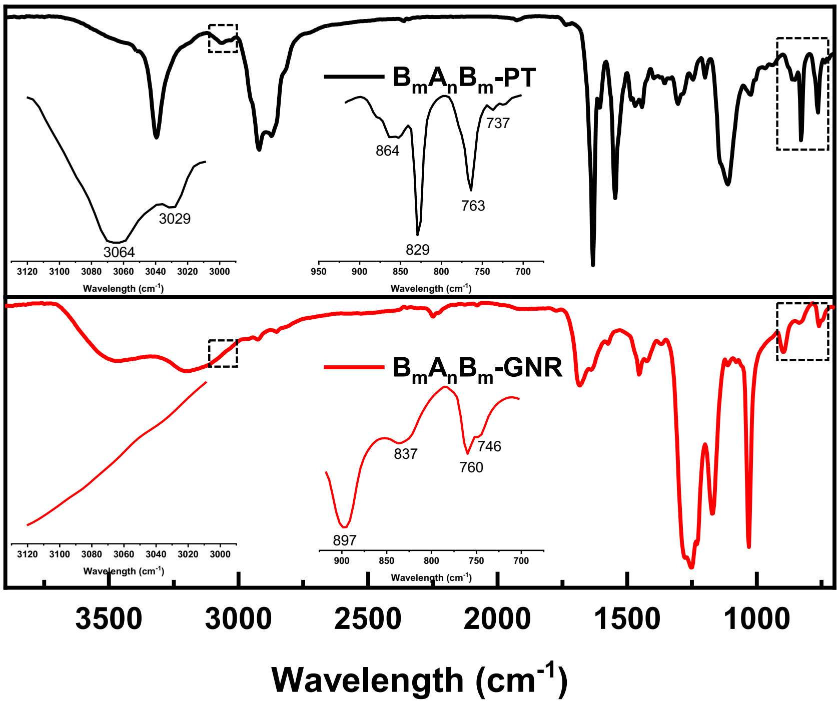

Supplementary Figure 18. FTIR spectra of $B_mA_nB_m$-PT and $B_mA_nB_m$-GNR after cyclodehydrogenation. The evident attenuation of the peaks from aromatic C–H stretching vibrations at 3064 and 3029 cm^{-1} in spectrum of $B_mA_nB_m$-GNR, the disappearance of bands at 864, 829 and 737 cm^{-1} that attributed to mono- and di-substituted benzene rings, and the appearance of bands for aromatic C–H at the edge positions at 897, 837 and 746 cm^{-1} confirm the cyclodehydrogenation of the precursor polyterphenyls[1-2].

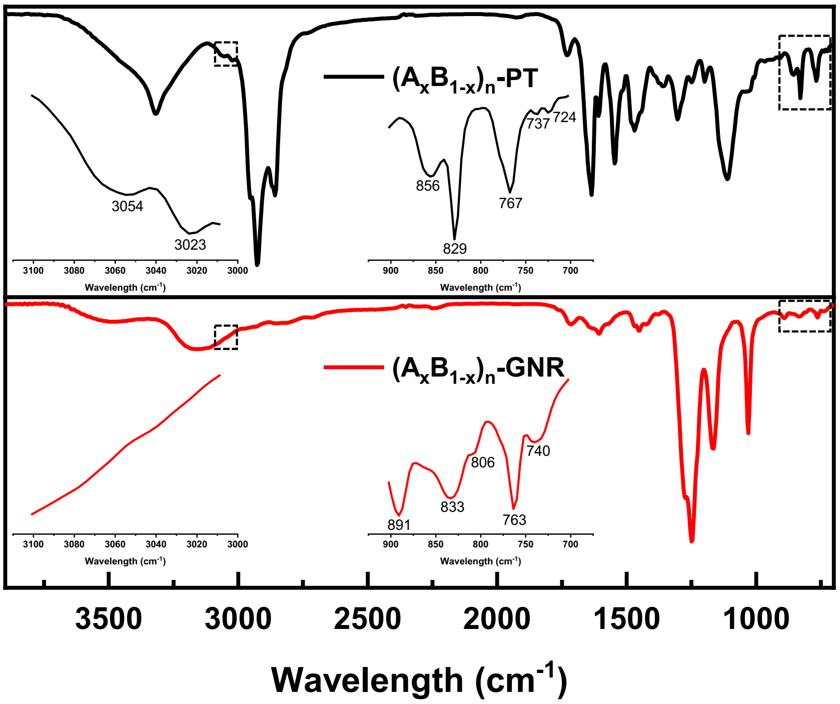

Supplementary Figure 19. FTIR spectra of $(A_xB_{1-x})_n$-PT and $(A_xB_{1-x})_n$-GNR after cyclodehydrogenation. The evident attenuation of the peaks from aromatic C–H stretching vibrations at 3054 and 3023 cm^{-1} in spectrum of $(A_xB_{1-x})_n$-GNR, the disappearance of bands at 856, 829, 737 and 724 cm^{-1} that attributed to mono- and di-substituted benzene rings, and the appearance of bands for aromatic C–H at the edge positions at 891, 833, 806 and 740 cm^{-1} confirm the cyclodehydrogenation of the precursor polyterphenyls[1-2].

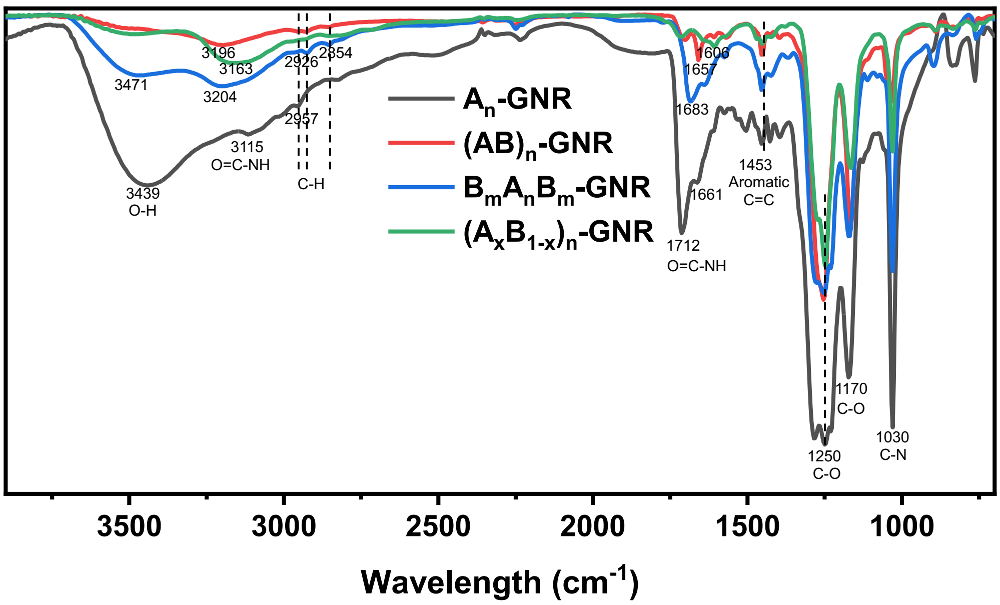

Supplementary Figure 20. FTIR spectra of A_n-GNR, $(AB)_n$-GNR, $B_mA_nB_m$-GNR, and $(A_xB_{1-x})_n$-GNR.

12. Supplementary Raman spectra

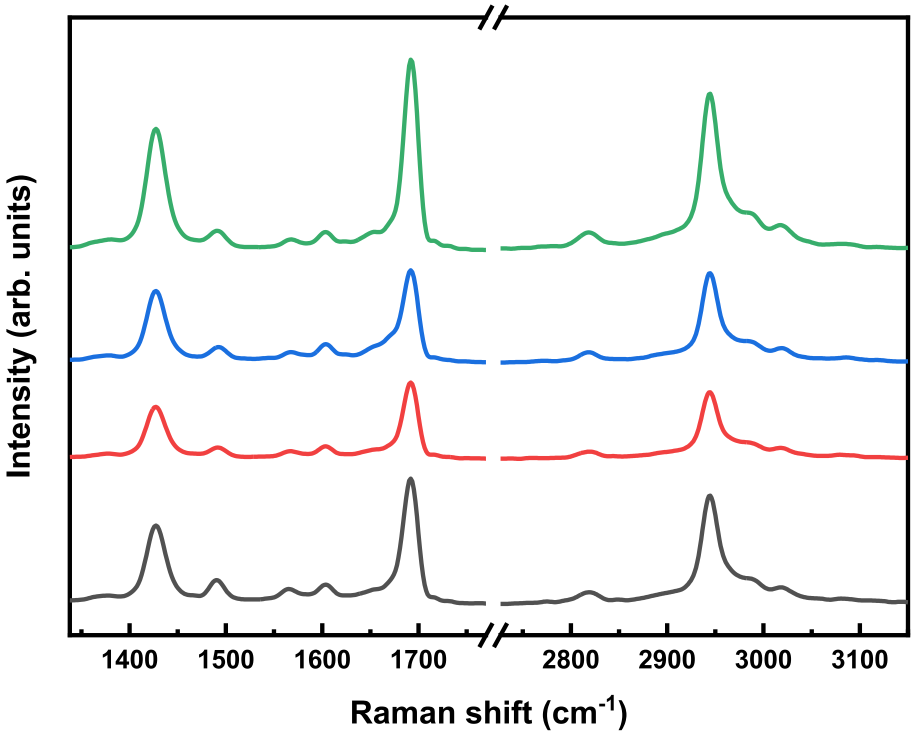

Supplementary Figure 21. Raman spectra of four side-chain sequence-regulated GNRs (A_n-GNR, $(AB)_n$-GNR, $B_mA_nB_m$-GNR, and $(A_xB_{1-x})_n$-GNR) upon excitation at 532 nm.

13. Supplementary TEM images

We have performed statistical analysis of the TEM data and the statistical results (size distributions and polydispersity index, PDI) are inserted in the corresponding TEM image. The size distributions of the self-assembled nanostructures are analyzed via FiberApp software[3] and the formula for PDI is presented as following equation:

$$\mathrm{PDI} = (\sigma/\mathrm{d})^2$$

Where, σ is the standard deviation divided and d is the mean particle diameter[4].

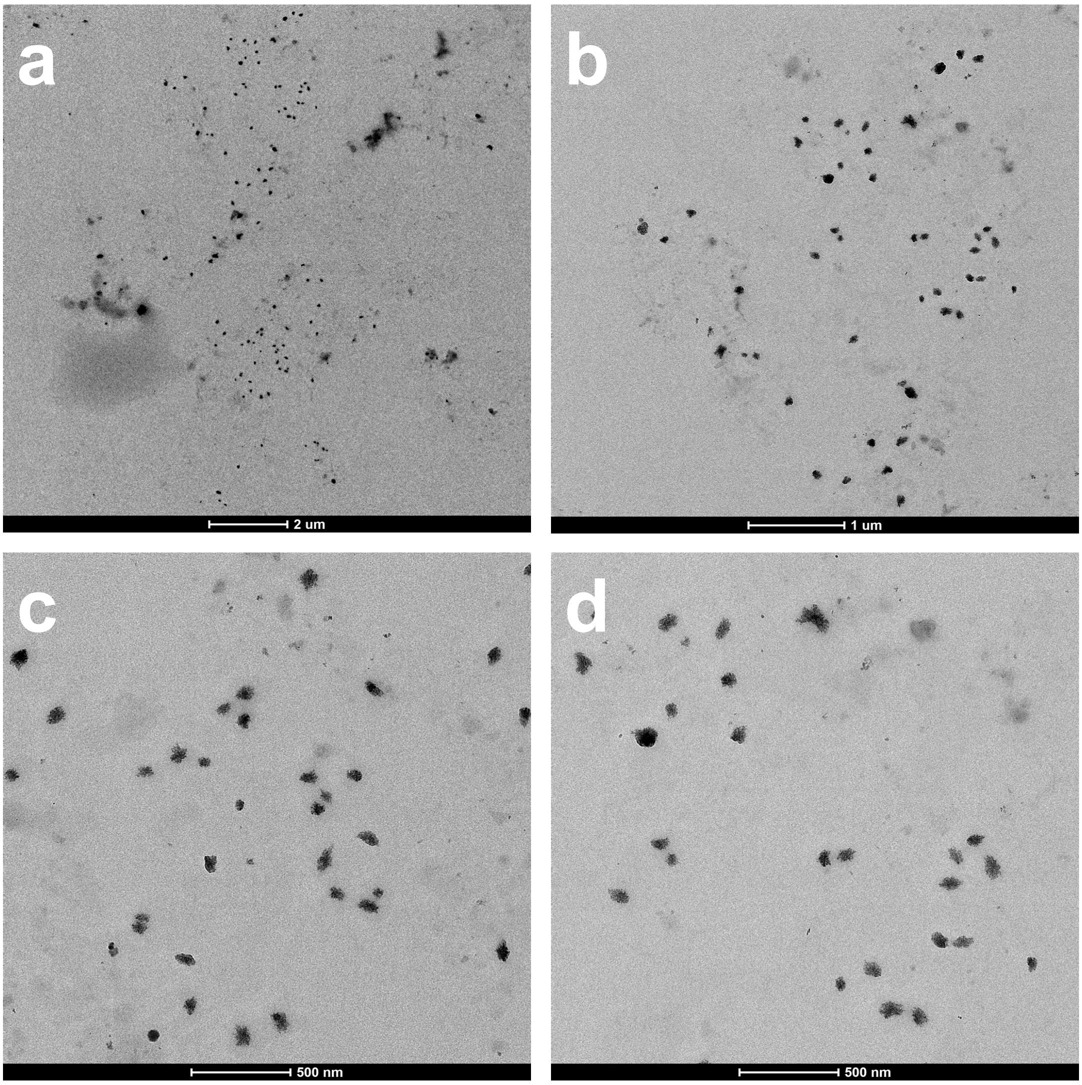

Supplementary Figure 22. (a-d) TEM images of nanostructures self-assembled from A_n-GNR in H_2O/DMF (v/v = 1:1).

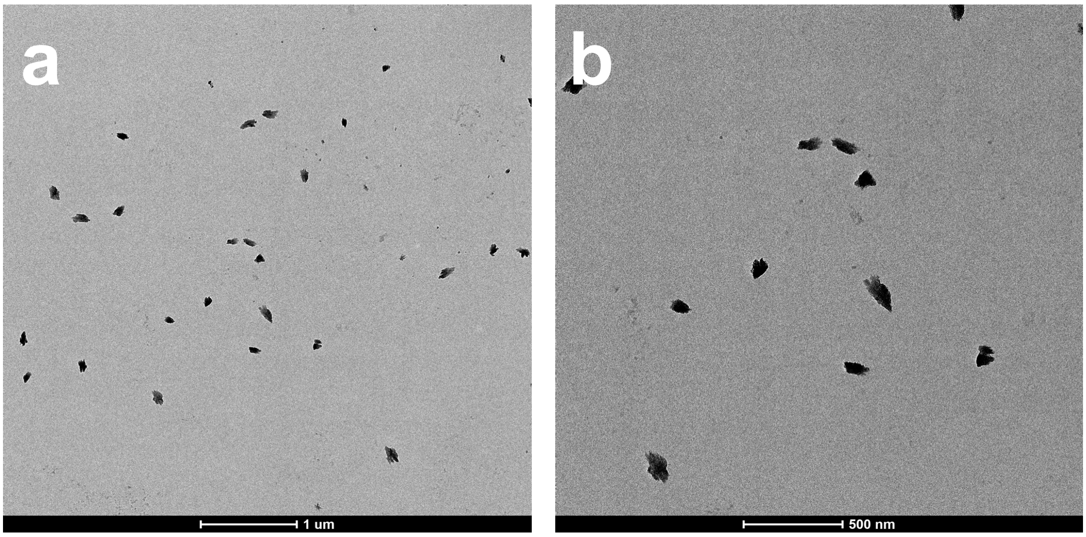

Supplementary Figure 23. (a-b) TEM images of nanostructures self-assembled from A_n-GNR in H_2O/DMF (v/v = 3:1).

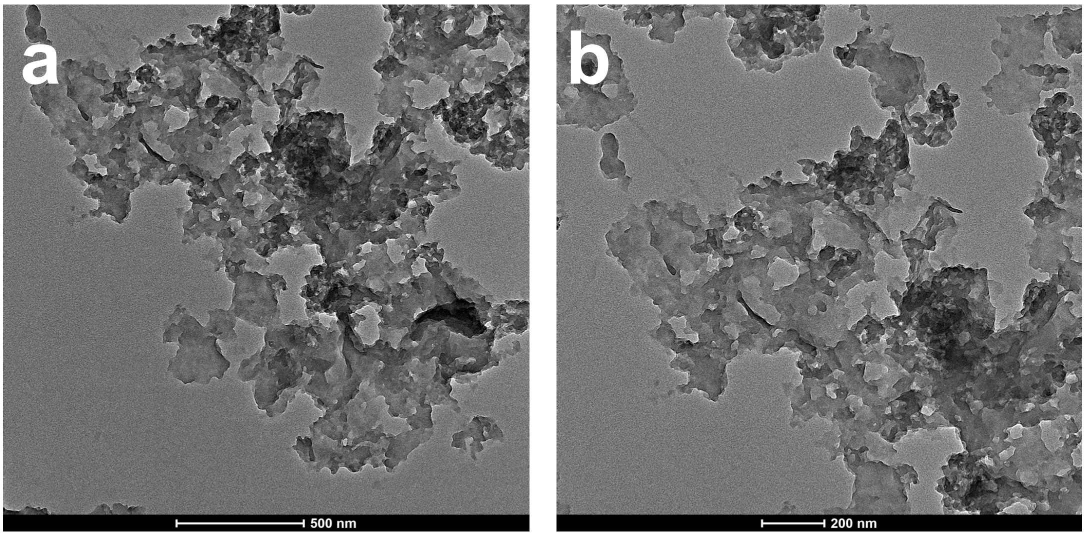

Supplementary Figure 24. (a-b) TEM images of nanostructures self-assembled from A_n-GNR in 100% H_2O.

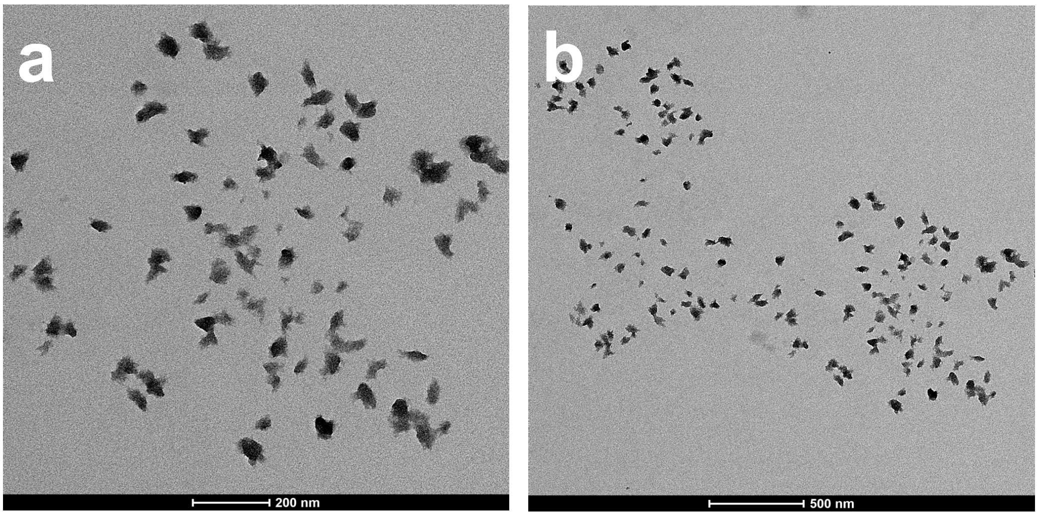

Supplementary Figure 25. (a-b) TEM images of nanostructures self-assembled from $(AB)_n$-GNR in H_2O/DMF (v/v = 1:1).

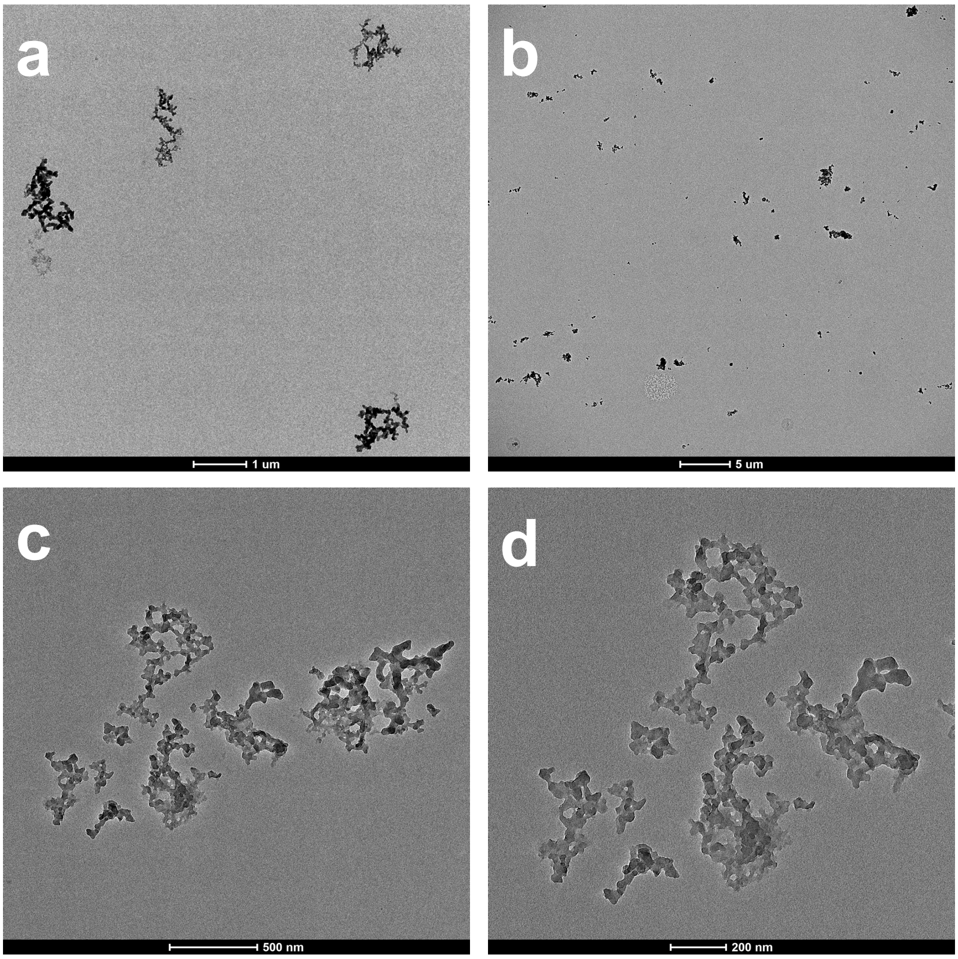

Supplementary Figure 26. (a-d) TEM images of nanostructures self-assembled from $(AB)_n$-GNR in H_2O/DMF (v/v = 3:1).

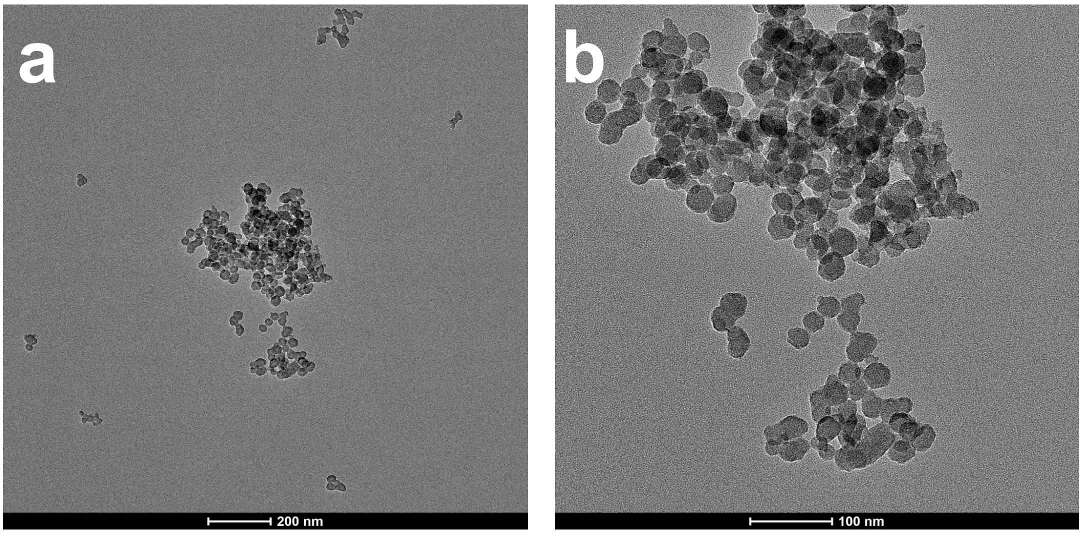

Supplementary Figure 27. (a-b) TEM images of nanostructures self-assembled from $(AB)_n$-GNR in 100% H_2O.

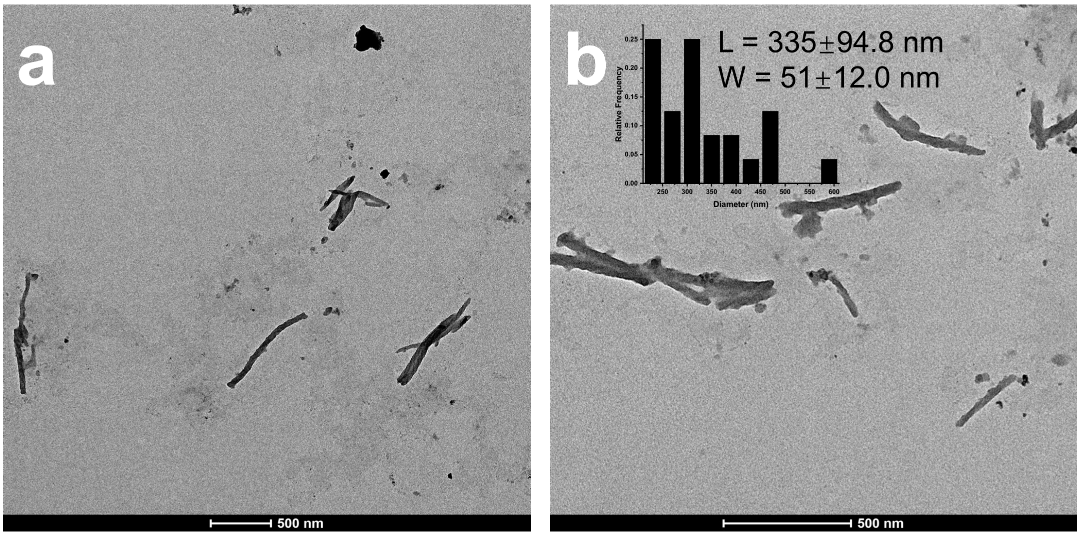

Supplementary Figure 28. (a-b) TEM images of nanostructures self-assembled from $B_mA_nB_m$-GNR in H_2O/DMF (v/v = 1:1).

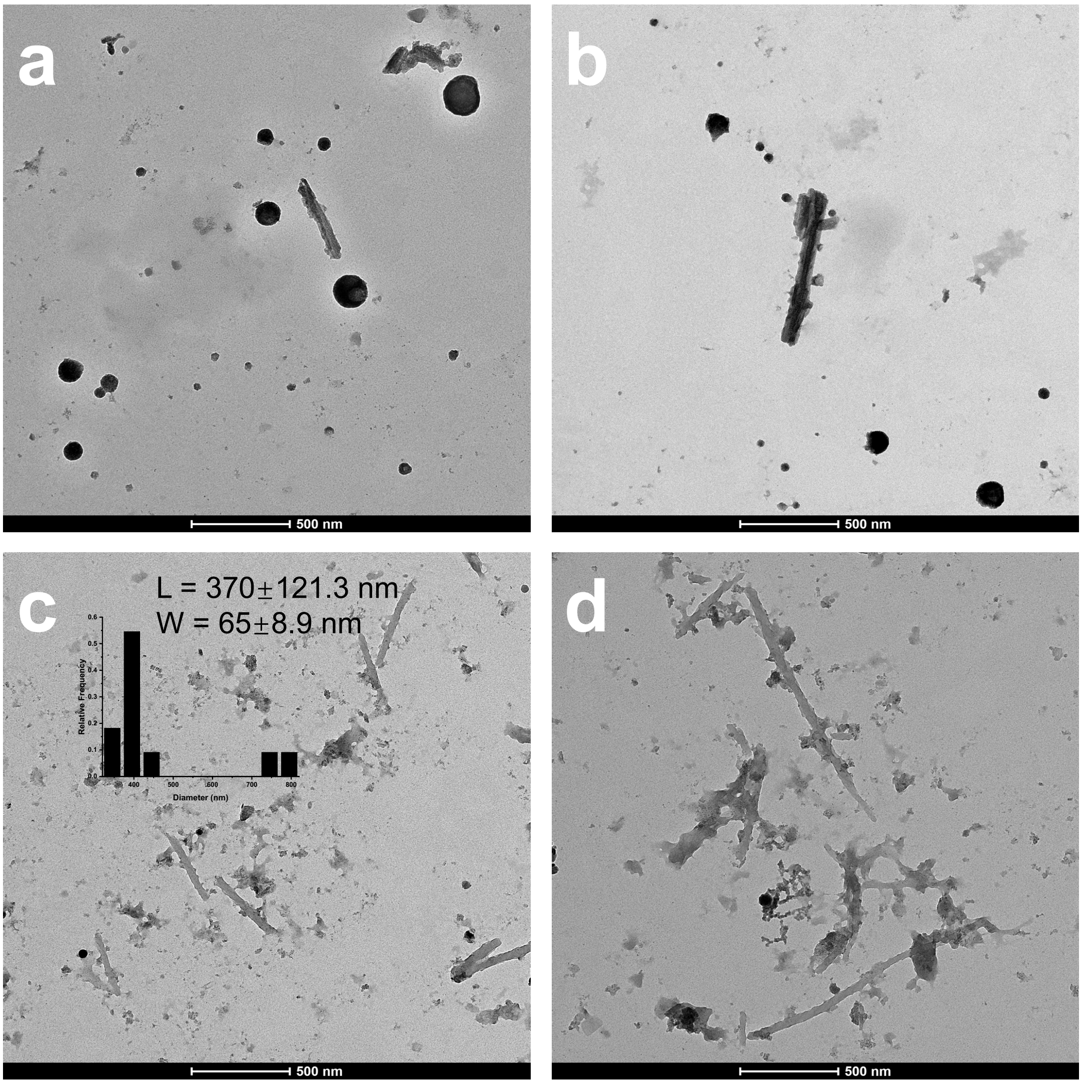

Supplementary Figure 29. (a-d) TEM images of nanostructures self-assembled from $B_mA_nB_m$-GNR in H_2O/DMF (v/v = 3:1).

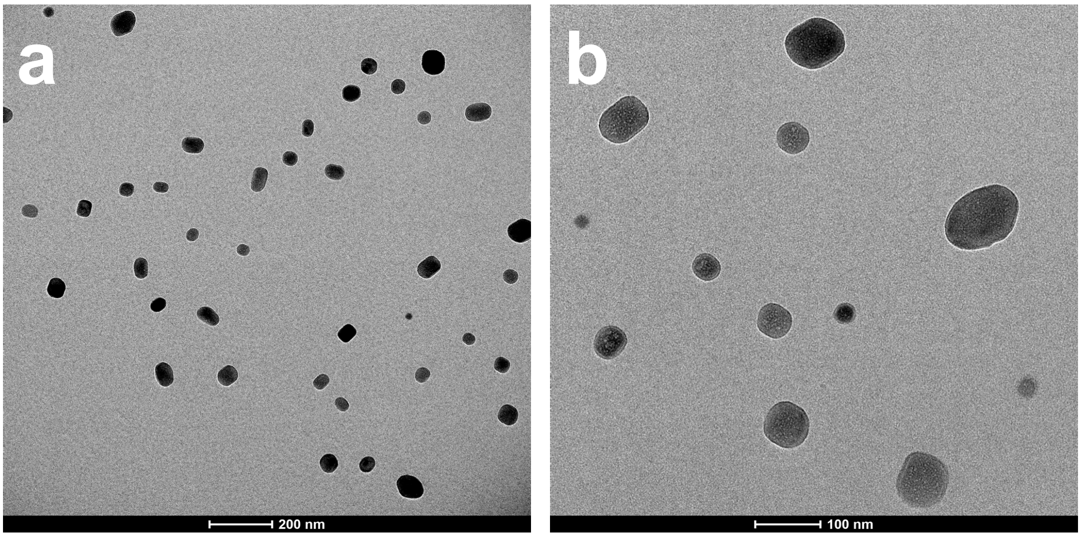

Supplementary Figure 30. (a-b) TEM images of nanostructures self-assembled from $B_mA_nB_m$-GNR in 100% H_2O.

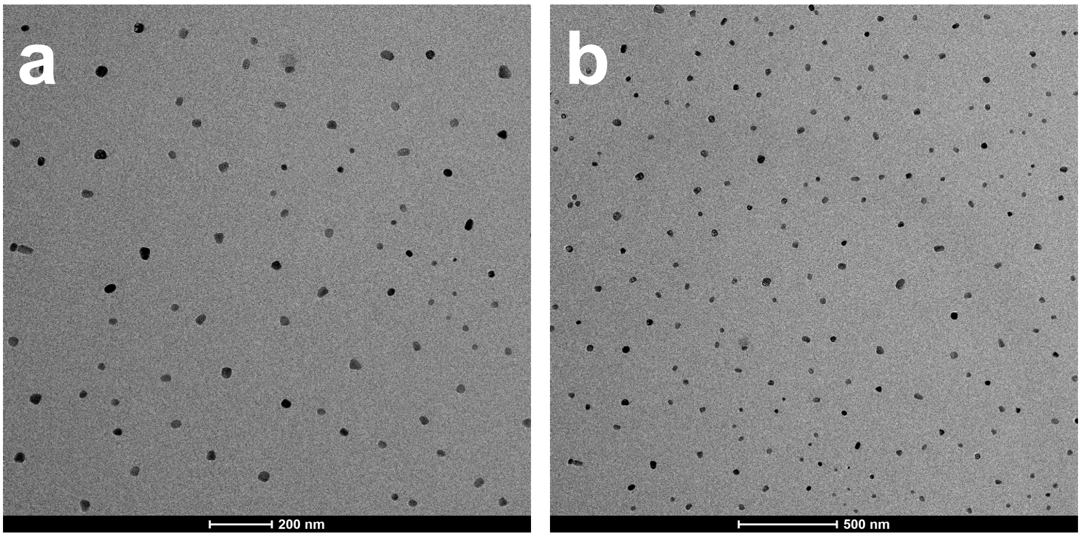

Supplementary Figure 31. (a-b) TEM images of nanostructures self-assembled from $(A_xB_{1-x})_n$-GNR in H_2O/DMF (v/v = 1:1).

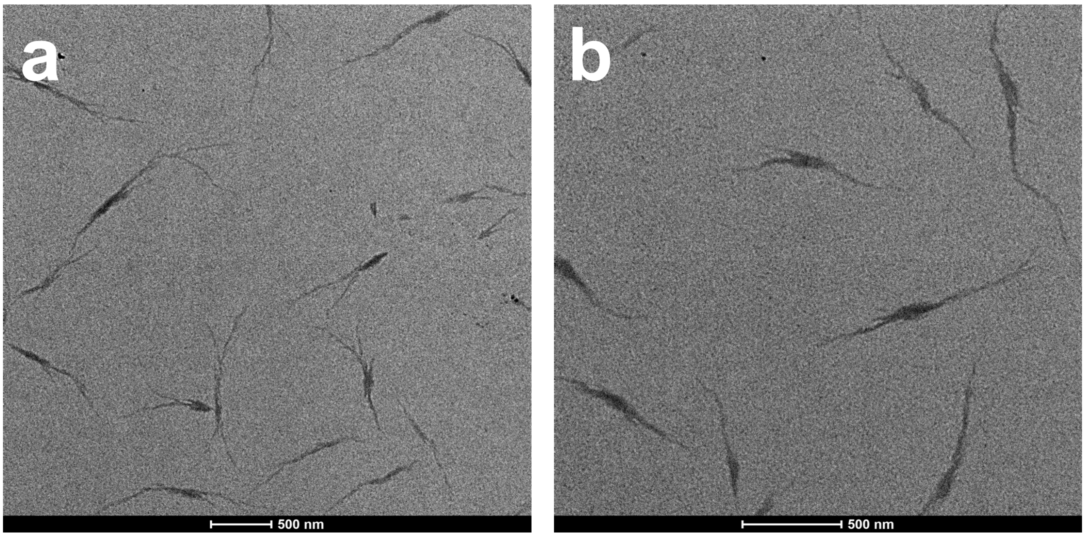

Supplementary Figure 32. (a-b) TEM images of nanostructures self-assembled from $(A_xB_{1-x})_n$-GNR in H_2O/DMF (v/v = 3:1).

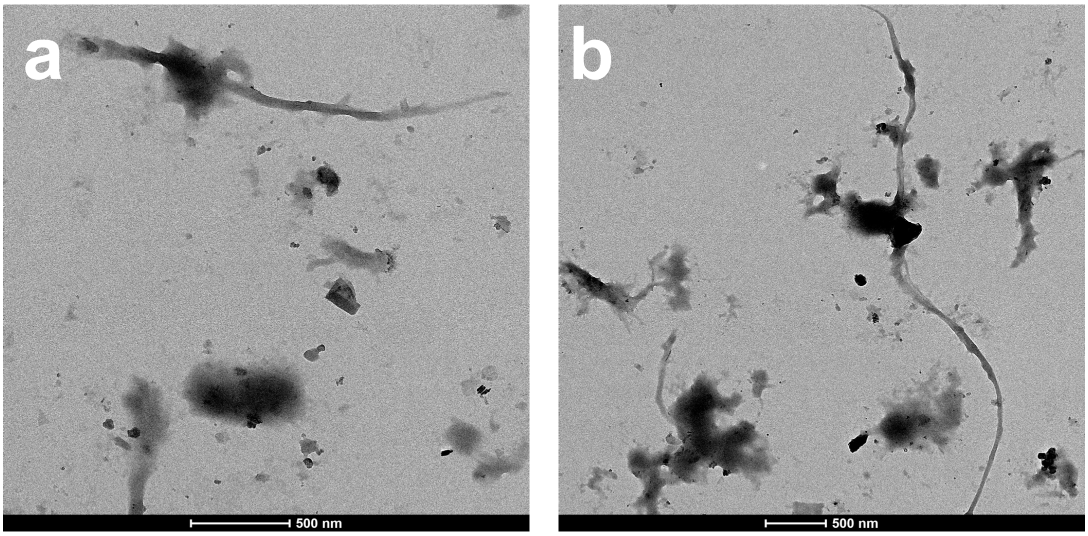

Supplementary Figure 33. (a-b) TEM images of nanostructures self-assembled from $(A_xB_{1-x})_n$-GNR in 100% H_2O/DMF.

14. Solubility and long-term stability of sequence-regulated GNRs

We investigated the solubility of four side-chain sequence-regulated graphene nanoribbons (GNRs) (i.e., A_n-, $(AB)_n$-, $B_mA_nB_m$-, and $(A_xB_{1-x})_n$-GNR) in H_2O/DMF mixed solutions. (**Supplementary Figure 34**).

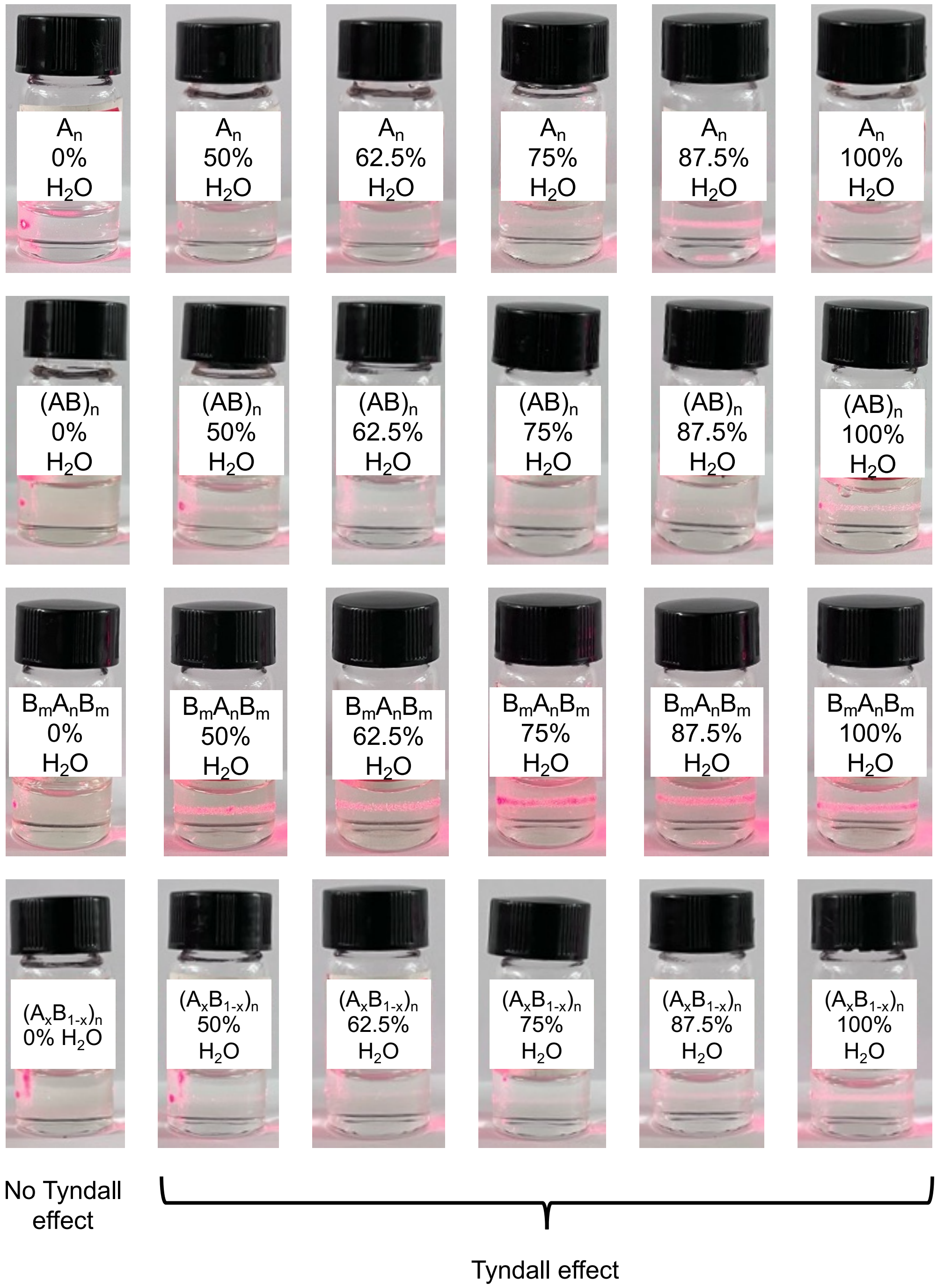

Supplementary Figure 34. Photographs of the four side-chain sequence-regulated graphene nanoribbons (i.e., A_n-, $(AB)_n$-, $B_mA_nB_m$-, and $(A_xB_{1-x})_n$-GNR) in H_2O/DMF mixed solutions.

As illustrated in Supplementary Figure 34, all side-chain sequence-regulated GNRs (A_n-, $(AB)_n$-, $B_mA_nB_m$-, and $(A_xB_{1-x})_n$-GNR) were soluble in DMF, to form optically transparent solutions showing no Tyndall effect (Willis-Tyndall scattering) with laser pen irradiation. The results confirmed the homogeneous nature of the DMF solutions of these GNRs.

We observed that these side-chain sequence-regulated GNRs (A_n-, $(AB)_n$-, $B_mA_nB_m$-, and $(A_xB_{1-x})_n$-GNR) were readily dispersed in H_2O/DMF mixed solutions, to form stable transparent colloidal solutions. Meanwhile, the resultant H_2O/DMF mixed solutions demonstrated a typical Tyndall effect, verifying the existence of molecular stacking behaviours of GNRs. In addition, these nanostructures would be readily dispersed in water to form stable milky colloidal solutions, showing typical Tyndall effect. This observation provided additional evidence for the formation of nanoscale aggregates in aqueous media, potentially attributing to their amphiphilic nature of these GNRs.

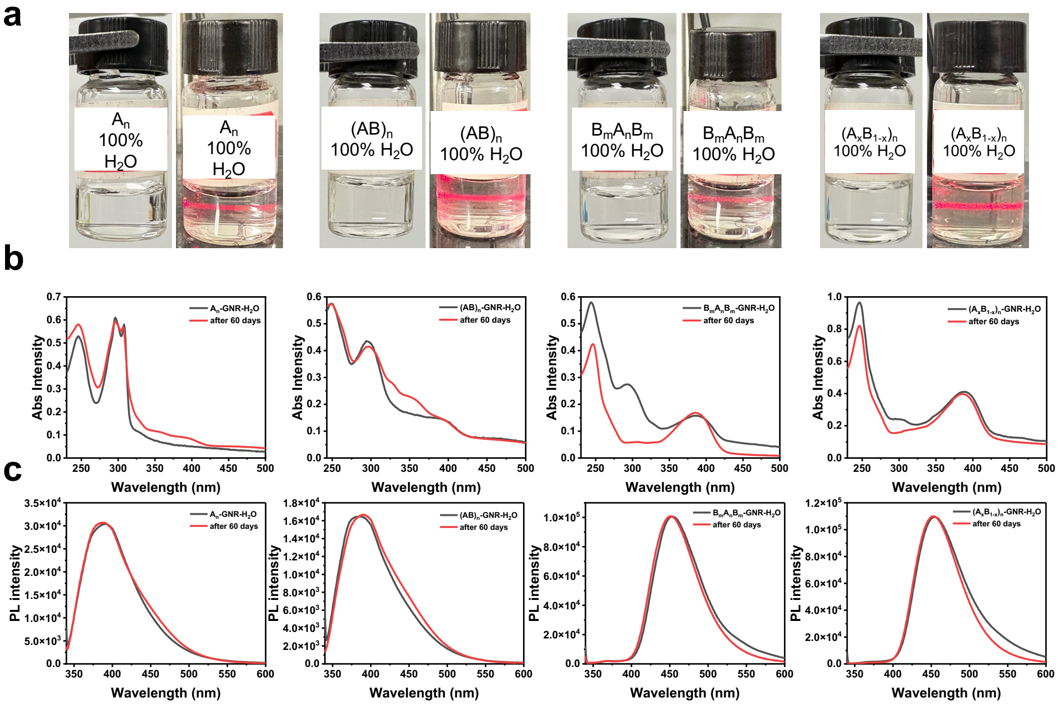

Supplementary Figure 35. (a) Photographs, (b) UV–vis absorption spectra, and (c) steady-state fluorescence spectra of four side-chain sequence-regulated GNRs (i.e., A_n-, $(AB)_n$-, $B_mA_nB_m$-, and $(A_xB_{1-x})_n$-GNR) in water after 60 days of storage.

Toward potential biomedical applications, the long-term colloidal stability and spectral

reproducibility of the GNRs were evaluated (Supplementary Figure 35). After approximately 60 days of storage, the aqueous GNR dispersions (50 μg/mL) remained clear and transparent, with no visible precipitation (Supplementary Figure 35a). The persistent Tyndall effect further confirmed that the GNRs remained well dispersed in the aqueous phase throughout this period, indicating stable colloidal behavior (Supplementary Figure 35a). Collectively, the photographs of the GNR dispersions and the observed Tyndall effect after 60 days demonstrate the macroscopic colloidal stability of the GNRs.

We further examined the long-term reproducibility of the UV–vis and photoluminescence (PL) spectra of the GNR dispersions (50 μg/mL). As shown in Supplementary Figure 35b,c, UV–vis and PL spectra with absolute intensities were recorded over a 60-day period. While the PL spectra of the side-chain-regulated GNRs remained almost unchanged over 60 days, their UV–vis spectra exhibited varying degrees of intensity change during storage. These spectral variations are likely attributable to gradual aggregation of the colloids in aqueous solution, a trend that is consistent with the optical data presented in Figure 5 of the main manuscript. Specifically, the aggregation states of GNR nanostructures are sensitive to the composition of mixed solution systems, leading to corresponding changes in their optical responses. Notably, the UV–vis spectra of $B_mA_nB_m$-GNR exhibited more pronounced variations than those of the other sequence-controlled GNRs, revealing a clear sequence dependence in the long-term optical and colloidal stability. This observation suggests that further optimization of GNR side-chain sequences represents a promising strategy for enhancing dispersion stability. Alternatively, incorporation of longer polyethylene glycol (PEG) chains has been shown to improve aqueous dispersibility and thereby enhance both colloidal and optical stability[5]. Beyond molecular design, non-covalent modification offers another effective approach to improving dispersion stability[6]. For example, surfactants such as cetyltrimethylammonium bromide (CTAB) and sodium dodecyl sulfate (SDS) can adsorb onto graphene surfaces via van der Waals and hydrophobic interactions, forming a stabilizing surfactant monolayer that introduces electrostatic repulsion and suppresses π–π stacking, thus maintaining stable aqueous dispersions[7].

15. Quantitative analysis of UV-Vis, PL and transient PL

a

GNR	Stacking mode	UV-Vis shift	PL shift
A_n	H-stacking (π-π)	0	0 (quenching)
$(AB)_n$	H-stacking (π-π)	0	0 (quenching)
$B_mA_nB_m$	J-stacking	30 nm (red-shift) from 356 to 386 nm	77 nm (red-shift) from 377 to 454 nm
$(A_xB_{1-x})_n$	J-stacking	38 nm (red-shift) from 352 to 390 nm	76 nm (red-shift) from 379 to 455 nm

b

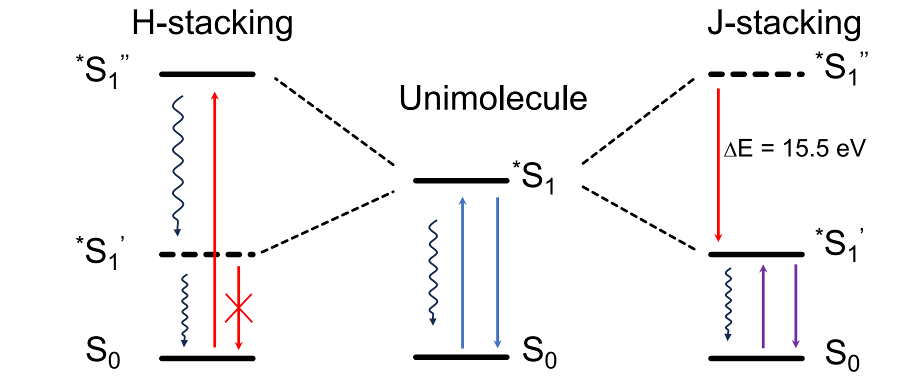

c

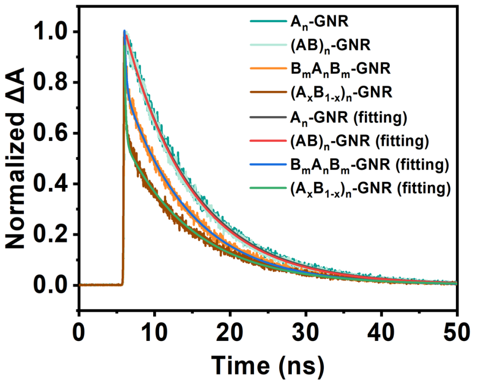

GNR	t_{av}	R-Square (COD)
A_n	8.873±1.008	0.999
$(AB)_n$	9.130±0.337	0.999
$B_mA_nB_m$	0.241±0.006	0.998
$(A_xB_{1-x})_n$	0.179±0.003	0.997

Supplementary Figure 36. (a) Quantification of UV–vis and PL peak shifts. (b) Proposed energy-level diagram of side-chain sequence-regulated GNRs in unimolecular states and H- and J-type stacking modes. (c) Lifetime fitting of transient PL decays for four side-chain sequence-regulated GNRs in water.

We summarized the absorption and emission peak shifts of the GNRs in Supplementary Figure 36a. A_n- and $(AB)_n$-GNRs predominantly adopt an H-stacking configuration and exhibit

no appreciable shifts in either the UV–vis absorption or PL spectra. However, pronounced PL quenching is observed, indicating that non-radiative relaxation processes dominate in the H-stacking mode. In contrast, $B_mA_nB_m$- and $(A_xB_{1-x})_n$-GNRs primarily adopt a J-stacking configuration and display clear red shifts in both absorption and emission spectra. Specifically, red shifts of 30 and 38 nm are observed in the UV–vis spectra, while larger red shifts of 77 and 76 nm are detected in the PL spectra, respectively. To facilitate interpretation of these spectral shifts, the corresponding optical peak positions are additionally provided in Supplementary Figure 36a. Notably, no significant PL quenching is observed for the J-stacked GNRs, suggesting that radiative relaxation processes dominate in this stacking state.

To facilitate interpretation of the spectral shifts, the corresponding optical peak positions are summarized in Supplementary Figure 36a. The associated energy difference (≈15.5 eV) was calculated from the ~80 nm PL red shift observed for $B_mA_nB_m$- and $(A_xB_{1-x})_n$-GNRs using the standard wavelength–energy conversion. To avoid potential ambiguity, the term "radiative decay" has been removed from the energy-level diagrams in Figure 5e and Supplementary Figure 36b. The wavelength-to-photon-energy conversion formula (E, in eV) is provided below:

$$E(eV) = \frac{hc}{\lambda}$$

Where, h is Planck's constant (6.626×10^{-34} J·s), c is speed of light (2.998×10^{8} m/s) and 1 eV is equal to 1.602×10^{-19} J.

Finally, transient PL decay curves were fitted to extract the PL lifetimes of the GNRs (Supplementary Figure 36c). The radiative lifetimes of $B_mA_nB_m$- and $(A_xB_{1-x})_n$-GNRs (0.24 and 0.18 ns, respectively) are markedly shorter than those of A_n- and $(AB)_n$-GNRs (8.9 and 9.1 ns, respectively), further corroborating that J-stacking is the dominant aggregation mode in $B_mA_nB_m$- and $(A_xB_{1-x})_n$-GNRs.

16. Synthesis of the triblock precursor $B_mA_nB_m$-PT and $(A_xB_{1-x})_n$-PT

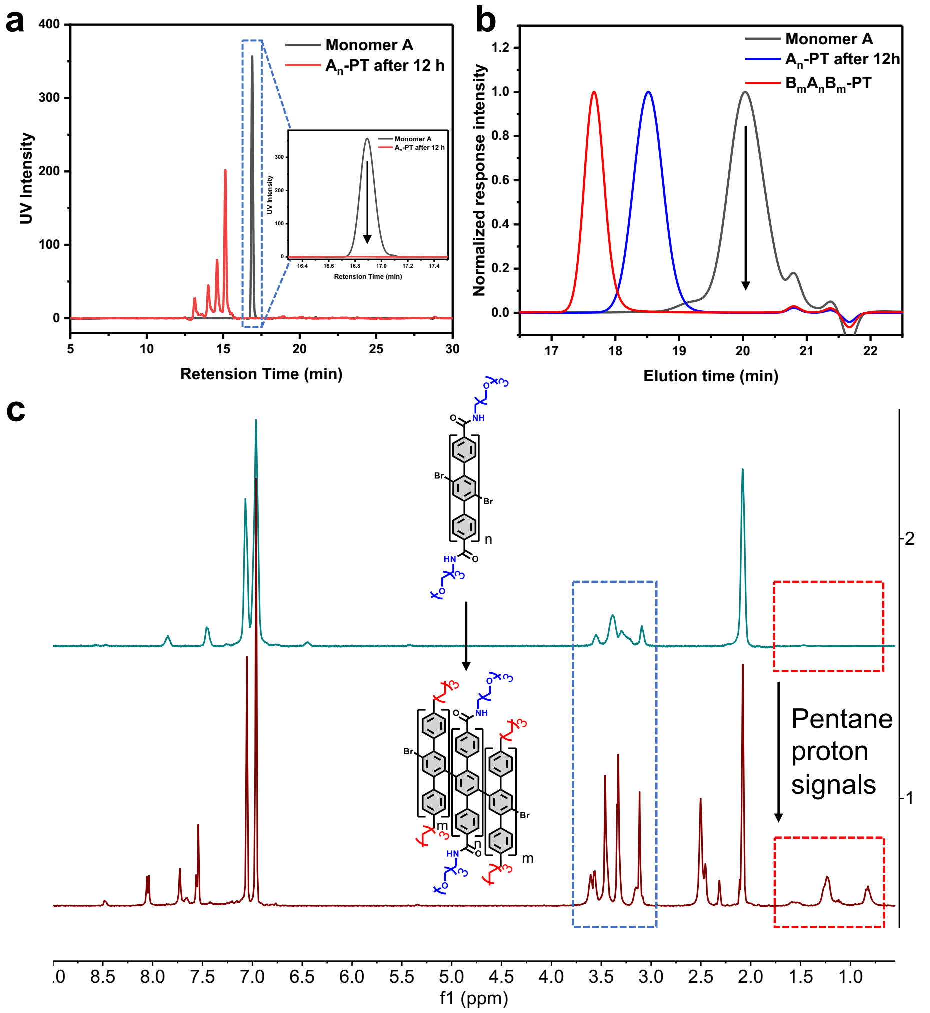

Supplementary Figure 37. (a) HPLC traces of monomer A and the intermediate A_n-PT. (b) SEC traces (DMF eluent) of monomer A, A_n-PT, and $B_mA_nB_m$-PT. (c) ^{1}H NMR spectra of A_n-PT and $B_mA_nB_m$-PT in toluene-d_8 at 378 K.

Accordingly, high-performance liquid chromatography (HPLC) and size-exclusion chromatography (SEC) were used to monitor the first-stage polymerization of monomer A (Supplementary Figure 37). After 12 h, the HPLC peak of monomer A disappeared completely, yielding the intermediate A_n-PT and confirming near-quantitative conversion before addition of monomer B (Supplementary Figure 37a). SEC analysis likewise showed complete disappearance of the monomer A peak with a clear shift to higher molecular weight, confirming formation of A_n-PT, demonstrating preserved chain-end reactivity (Supplementary Figure 37b).

Upon addition of monomer B, A_n-PT was fully consumed within 24 h, yielding higher-molecular-weight species with a clear peak shift, consistent with chain extension to triblock $B_mA_nB_m$-PT (Supplementary Figure 37b). The ^{1}H NMR spectrum of A_n-PT showed only EG_3 side-chain signals, whereas $B_mA_nB_m$-PT displayed additional resonances from pentane side chains, confirming incorporation of B_m-PT blocks at both chain ends (Supplementary Figure 37c). We note that the reported 51% yield corresponds to the isolated yield after precipitation-based separation, which typically results in product loss. Collectively, HPLC, SEC, and ^{1}H NMR analyses validate near-quantitative monomer conversion and high chain-extension efficiency.

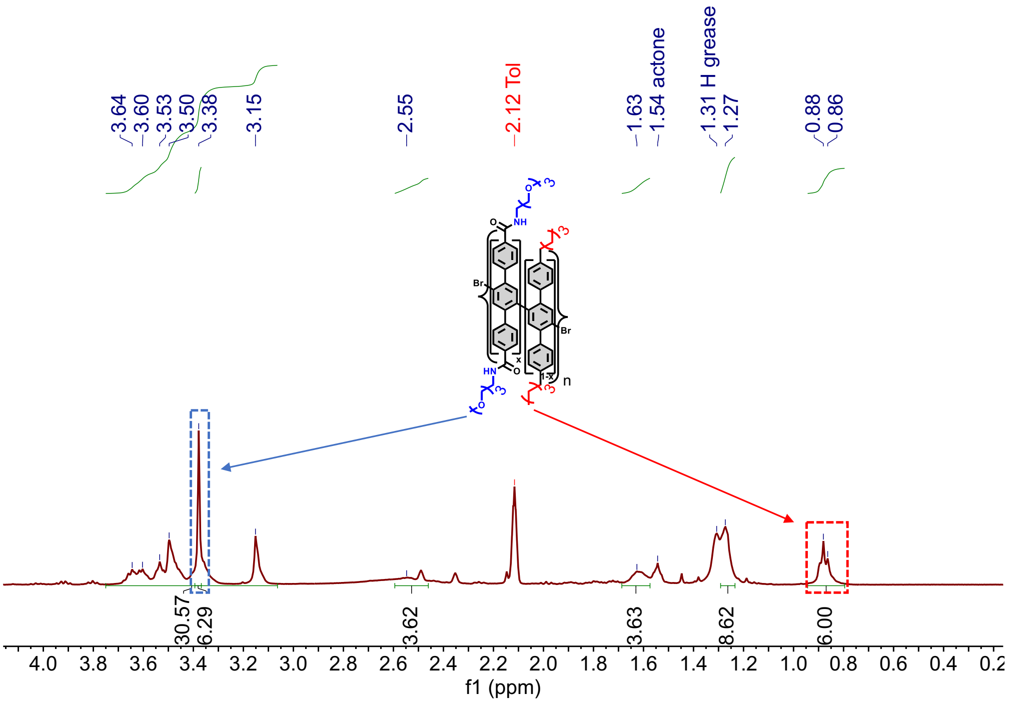

Supplementary Figure 38. ^{1}H NMR spectrum of $(A_xB_{1-x})_n$-PT showing integration of terminal methyl (CH_3) groups from EG_3 and pentane side chains in toluene-d_8 at 378 K.

The proton nuclear magnetic resonance (^1H NMR) signals corresponding to the EG_3 and pentane side-chain terminals of $(A_xB_{1-x})_n$-PT were integrated, yielding an EG_3-to-pentane ratio of approximately 1.05 : 1. This value closely matches the 1 : 1 feeding ratio of monomers A and B (Supplementary Figure 38). The ^{1}H NMR results indicate that monomers A and B exhibit comparable reactivities under the employed Yamamoto coupling conditions. This observation

confirms that the reactivity of terphenyl-based monomers is not significantly influenced by structural variations in their side chains.

17. Correlation between the block length and the red-shift degree of PL

To investigate the quantitative relationship between block length and the red-shift of PL spectra, we systematically tuned the block lengths *m* and *n* in $B_mA_nB_m$-GNR by adjusting the feed amount of monomer B from 1.0 to 2.0 equivalents. In our standard synthesis, $B_mA_nB_m$-GNR was prepared via sequential Yamamoto polymerization, in which 1.0 equiv. of monomer A was first added, followed by 1.0 equiv. of monomer B. To selectively increase the block length *m* while maintaining *n* constant, identical reaction conditions were used, except that the amount of monomer B added in the second step was increased. For example, after the addition of 1.0 equiv. of monomer A, 2.0 equiv. of monomer B was introduced.

^{1}H NMR spectroscopy confirms the successful extension of block *m*. By integrating the characteristic signals corresponding to the EG_3 side chains and pentane units, the block length ratio of A to B was found to increase from approximately 1:0.42 to 1:0.79 (Supplementary Figure 39a,b), indicating a significantly longer B block in the resulting $B_mA_nB_m$-GNR. Despite this clear increase in block length, no noticeable difference in the degree of red-shift was observed in the PL spectra of $B_mA_nB_m$-GNR with different *m* values (Supplementary Figure 39c). These results indicate that the PL red-shift does not exhibit a direct quantitative correlation with block length in this system.

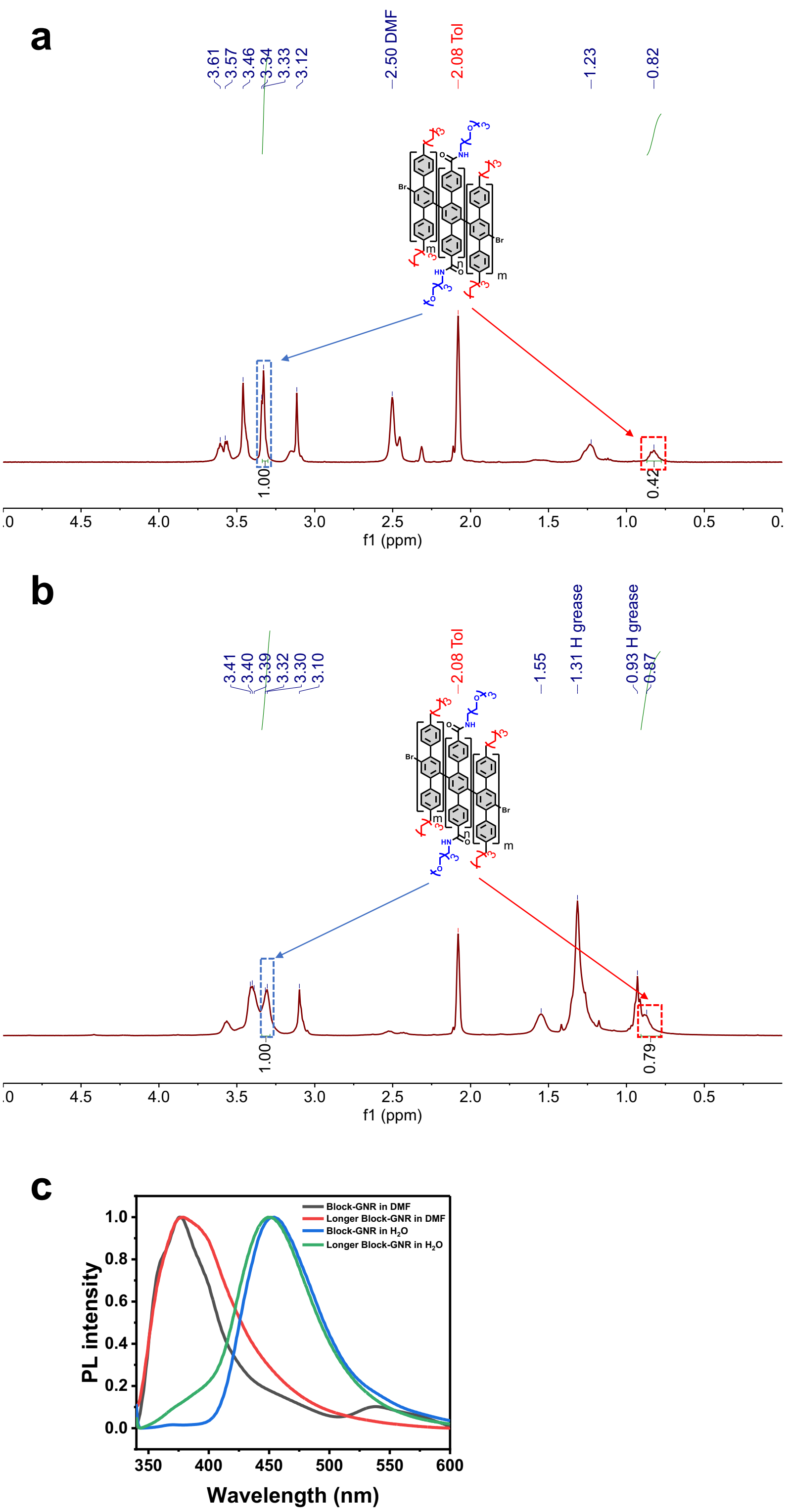

Supplementary Figure 39. (a) ^{1}H NMR spectrum of $B_mA_nB_m$-PT with a monomer A/B feed ratio of 1:1 in toluene-d_8 at 378 K. (b) ^{1}H NMR spectrum of $B_mA_nB_m$-PT with a monomer A/B feed ratio of 1:2 in toluene-d_8 at 378 K. (c) PL spectra of $B_mA_nB_m$-PT with different A/B block-length ratios.

18. Potential applications in fields of biomedicine and optoelectronics

Well-controlled self-assembled nanostructures are directly associated with regulated molecular stacking modes. Previous studies have shown that GNRs in solution typically suffer from severe fluorescence quenching in the absence of controllable hierarchical organization[5]. Notably, the J-stacking mode, characterized by pronounced spectral red shifts without significant fluorescence quenching, confers GNRs with considerable potential for use as fluorescent probes in biological environments. Moreover, owing to the excellent dispersibility of side-chain-modified GNRs in common organic solvents such as DMF, THF, and DCM, uniform thin films were readily fabricated on glass substrates (1 × 1 cm) via spin coating. This result further demonstrates the good film-forming capability of these GNR systems.

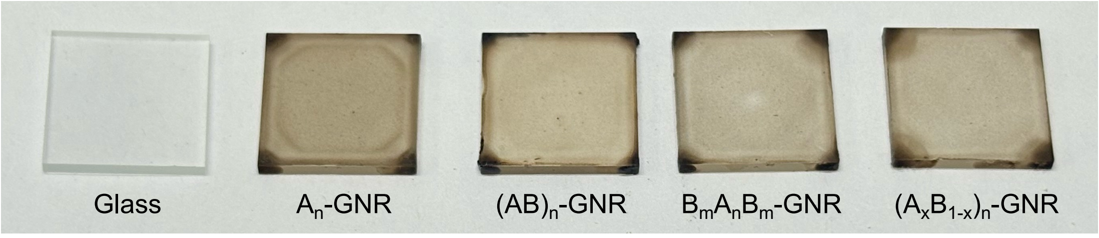

Supplementary Figure 40. Photographs showing the formation of thin films of four types of GNRs with regulated side-chain sequences (A_n-, $(AB)_n$-, $B_mA_nB_m$-, and $(A_xB_{1-x})_n$-GNR) deposited on glass substrates by spin coating from DCM solutions.

19. Molecular dynamics simulations

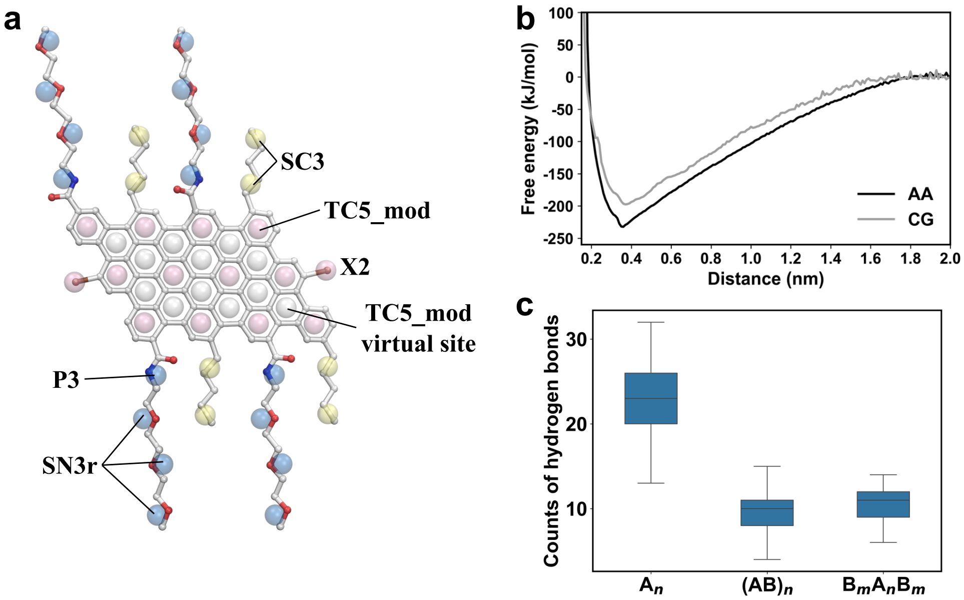

Supplementary Figure 41. (a) All-atom (AA) to coarse-grained (CG) mapping scheme and CG bead choices. The self-interactions of TC5 beads were modified to reproduce the dimer free energy between graphene sheets. (b) The dimer free energy of AA and CG graphene sheets estimated using the meta-dynamics method. (c) The number of hydrogen bonds formed between the H-stacked GNR models.

All-atom and coarse-grained model parameters

All simulations were performed using GROMACS[8] 2021.3 and 2025.2. The all-atom and coarse-grained models of GNRs were constructed based on the GAFF[9] and MARTINI 3[10] force fields. For all-atom models, the RESP[11] charges were calculated using the ORCA 5.0.4[12] and Multiwfn[13] packages. The single point energy calculations were performed using the B3LYP/G functional with the def2-TZVP basis set and D3 dispersion corrections implemented in ORCA 5.0.4, based on the geometries optimized using the GFN2-xTB method implemented in the xtb[14-15] program. The topology files were generated using the sobtop[16] package.

To develop CG parameters of GNRs, we referred to the standard MARTINI 3 parametrization workflow[17-18] and recently developed coarse-grained models for carbon nanomaterials[19]. The AA to CG mapping scheme was shown in Supplementary Figure 35a. The bonded parameters of GNR CG models were derived by fitting to atomistic simulations.

We further refined the nonbonded parameters between the graphene sheets in the GNRs CG models to reproduce the dimerization free energy obtained from well-tempered meta-dynamics (WT-MetaD) simulations[20] (Supplementary Figure 35b). In WT-MetaD simulations, the distance between the center-of-geometry (COG) of two graphene sheets and a torsion angle formed by particles in graphene sheets and COGs were selected as the collective variables (CV). The Gaussians with a height of 1.2 kJ/mol were deposited every 500 steps. The bias factor was set to 20. The widths of Gaussian hills were set to 0.05 nm for the COG distance and 0.3 rad for the torsion angle. A harmonic potential wall with a force constant of 1500 kJ/mol was placed at the COG distance of 2.0 nm to avoid exploring irrelevant conformations.

Coarse-grained MD simulation details

To investigate the assembly process, coarse-grained systems containing 650 A_n-GNR (10 wt%), 1400 $(AB)_n$-GNR (20 wt%), and 1400 $B_mA_nB_m$-GNR (20 wt%) molecules were constructed. The GNR CG models were randomly placed in a cubic box of ~30 × 30 × 30 nm^3, solvated with CG water beads. After energy minimization using the steepest descent algorithm, the system was equilibrated for 1 ns in the NPT ensemble. The temperature was maintained at 298 K using the velocity rescale thermostat[21]. The pressure was maintained at 1 bar using the stochastic cell rescaling barostat[22]. Subsequently, a 2-μs simulated annealing simulation was performed under constant volume conditions. The system was gradually heated from 298 K to 500 K over 200 ps, maintained at 500 K for 300 ps, and then symmetrically cooled to 298 K. Finally, a 2-μs production run was performed in the NPT ensemble. The reaction field method was used to deal with the electrostatic interactions.

All-atom MD simulation details

In all-atom MD simulations, the energy-minimization process was carried out using the steepest descent algorithm. After heating from 50 K to 298 K over 200 ps, the system was equilibrated at 1 bar in the NPT ensemble. The temperature and pressure were maintained using the velocity rescaling thermostat and the stochastic cell rescaling barostat, respectively. After equilibration, the production run was carried out.

The explore mode of the on-the-fly probability enhanced sampling (OPES) method[23-24]

was employed to estimate the stacking free energy and explore conformations. In OPES simulations, the initial models consist of four stacked GNR models. The coordination number and two distances between the center of mass (COM) of the two GNR sheets at the bottom and the two GNR sheets at the top were biased as collective variables. The bias interval was set to 10 ps. The barrier parameter was set to 80 kJ/mol.

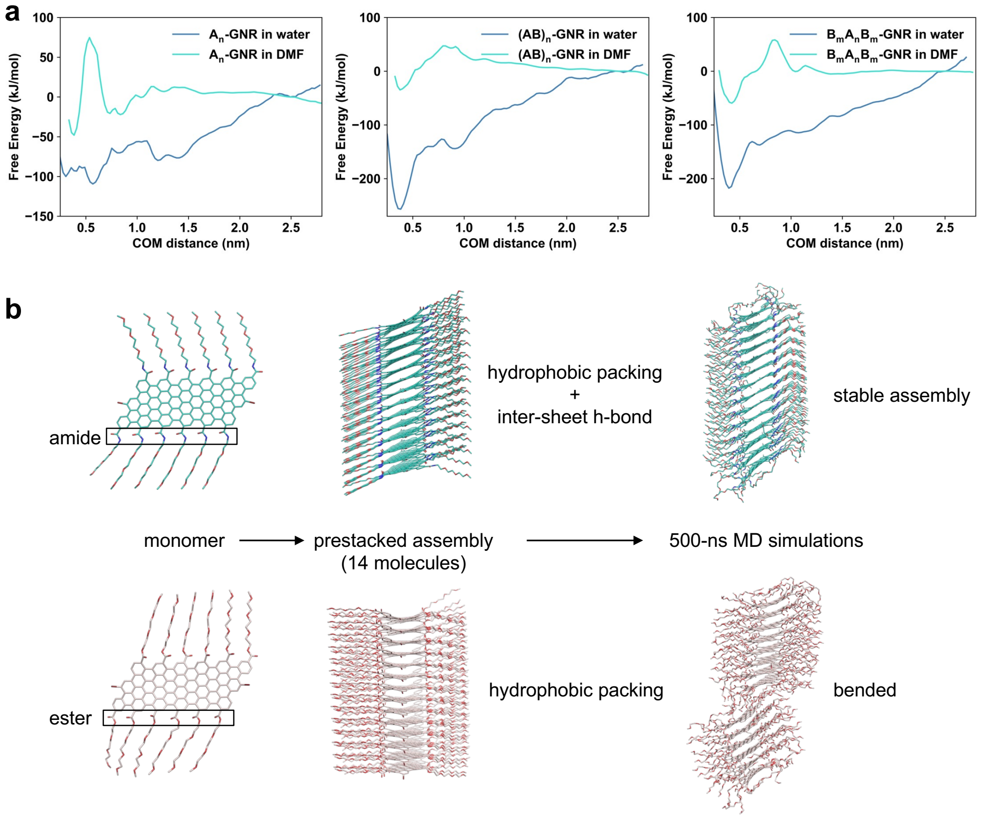

Supplementary Figure 42. (a) Dimerization free-energy landscapes of A_n-, $(AB)_n$-, and $B_mA_nB_m$-GNR. The distance between the centers of mass (COMs) of graphene cores was used as the collective variable in OPES-EXPLORE simulations. (b) MD snapshots of pre-stacked A_n- and $A^{ester}{}_n$-GNR models.

The transformations of GNR assemblies were investigated in mixed DMF–water solvents with varying water fractions. As the water content increased from 50% to 100%, BmAnBm-GNR assemblies underwent a clear morphological transition from helices to vesicles, indicating that solute–solvent interactions and solvent quality play a decisive role in regulating assembly behavior. To elucidate the solvent effect at the molecular level, dimerization free energies of

GNRs were calculated in pure DMF and pure water. In both solvents, the dimer was identified as the most thermodynamically stable configuration, characterized by tight packing of the hydrophobic graphene cores with a center-of-mass distance of approximately 0.35–0.5 nm (Supplementary Figure 42a).

Variations in solvent composition can reshape the free-energy landscape by introducing or removing metastable states and energy barriers, although the extent of this effect is system dependent. For $B_mA_nB_m$-GNR at a water fraction of 75%, the coexistence of rod-like and micellar structures is likely attributable to kinetic trapping. In the CG-MD simulations, GNR monomers were initially randomly distributed, and simulated annealing was applied by repeatedly heating the system to 500 K and cooling it to 298 K. This procedure facilitates barrier crossing and effectively suppresses kinetic trapping. Consequently, the vesicle and micelle morphologies observed for $B_mA_nB_m$- and $(AB)_n$-GNR in pure water are considered thermodynamically favored states. Furthermore, CG-MD simulations reveal that lateral hydrophobic packing between short GNR stacks plays a critical role in vesicle formation for $B_mA_nB_m$-GNR. Increasing the DMF fraction is therefore expected to weaken these hydrophobic interactions, disfavoring vesicle formation.

Beyond their role as synthetic linkers, the amide groups enable interlayer hydrogen bonding, which enhances the stability of GNR assemblies. All-atom MD simulations show that the H-aggregate of A_n-GNR, which contains the largest number of consecutive hydrogen bonds among the three GNR systems, exhibits the highest stability (Figure 6a–c). Motivated by previous studies on hydrogen-bond-assisted amplification in 1,3,5-benzenetricarboxamide (BTA) assemblies, we further constructed a pre-stacked A_n-GNR model comprising 14 molecules, along with an $A^{ester}{}_n$-GNR model in which the amide groups were replaced by ester groups. After 500 ns of MD simulation, the An-GNR stack remained stable and well ordered, whereas the $A^{ester}{}_n$-GNR stack displayed pronounced bending (Supplementary Figure 42b). Taken together, these results demonstrate that π–π stacking and hydrophobic interactions between graphene cores are dominant and sufficient to drive GNR self-assembly, while interlayer hydrogen bonding plays a reinforcing role that is critical for stabilizing larger GNR stacks.

20. Supplementary NMR spectra

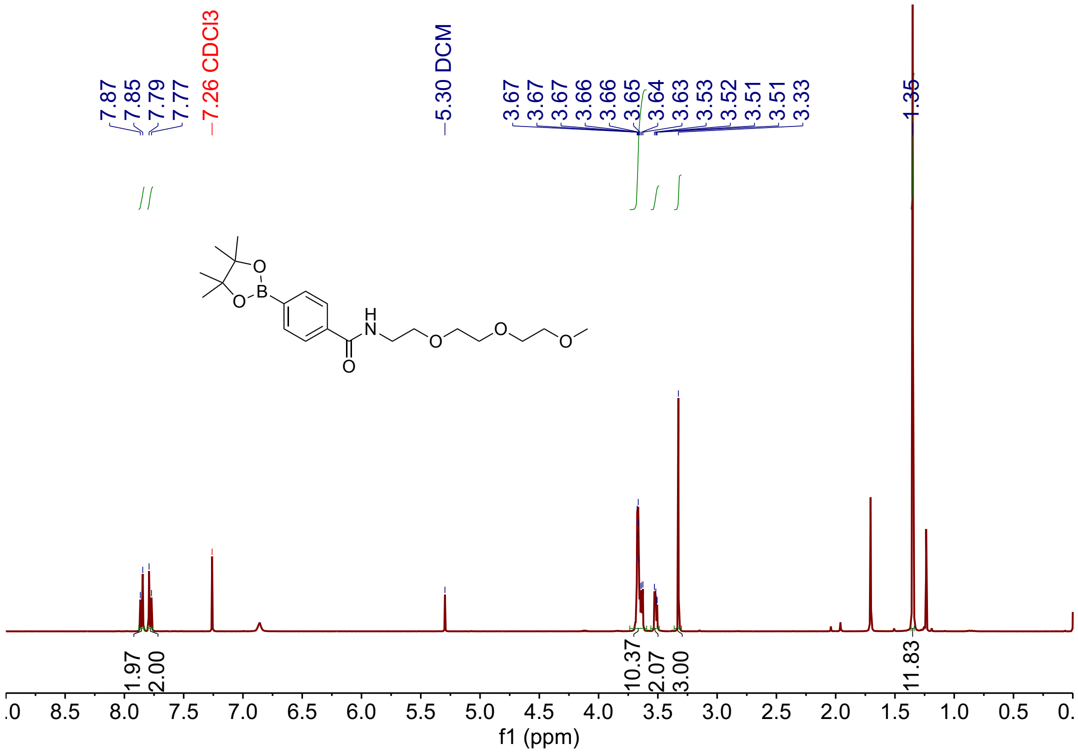

Supplementary Figure 43. ^{1}**H NMR spectrum of S2 in** $CDCl_3$ **(298 K).**

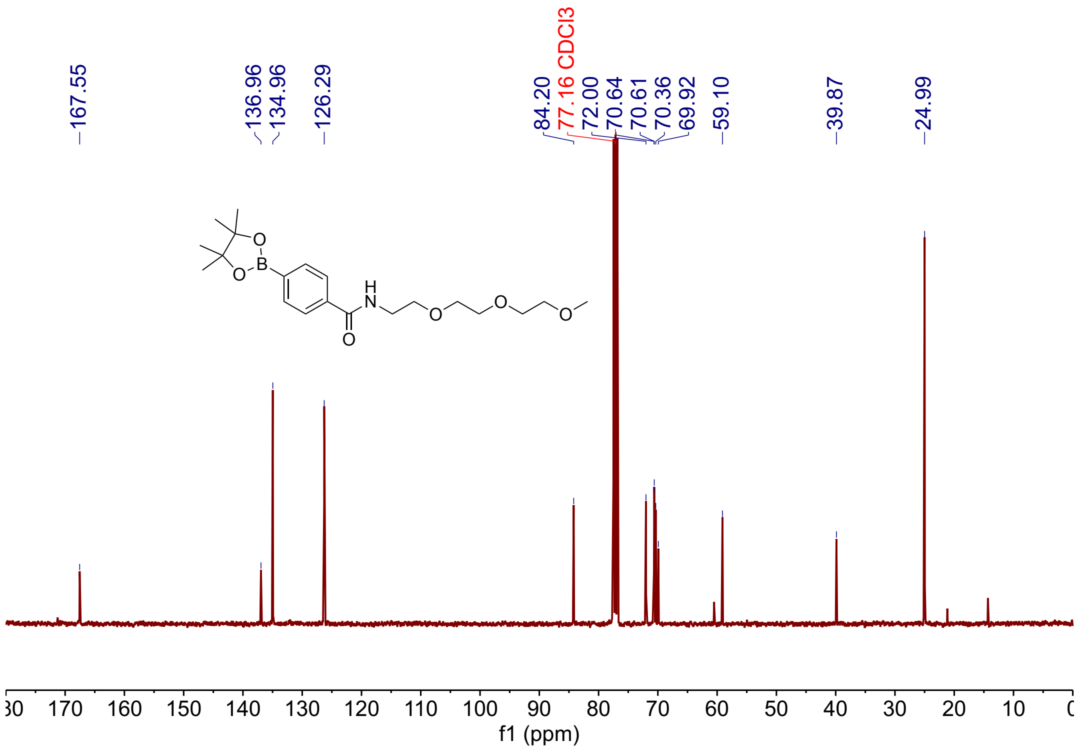

Supplementary Figure 44. ^{13}**C NMR spectrum of S2 in** $CDCl_3$ **(298 K).**

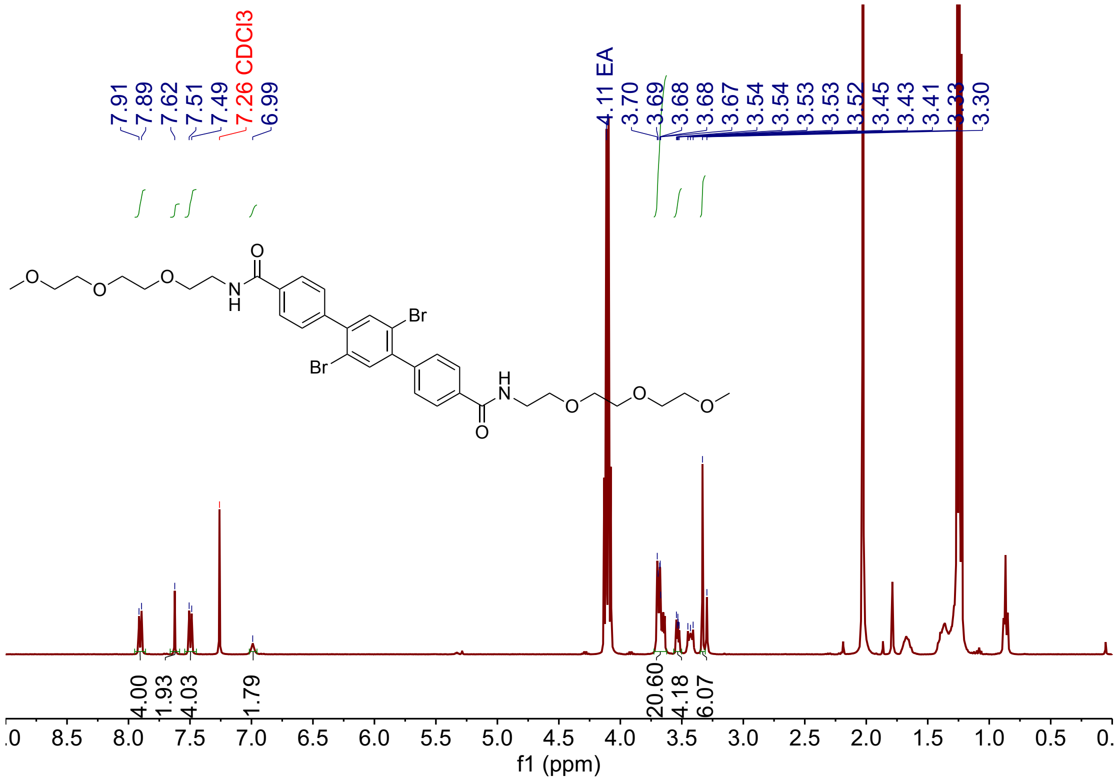

Supplementary Figure 45. ^{1}H NMR spectrum of **S3** in $CDCl_3$ (298 K).

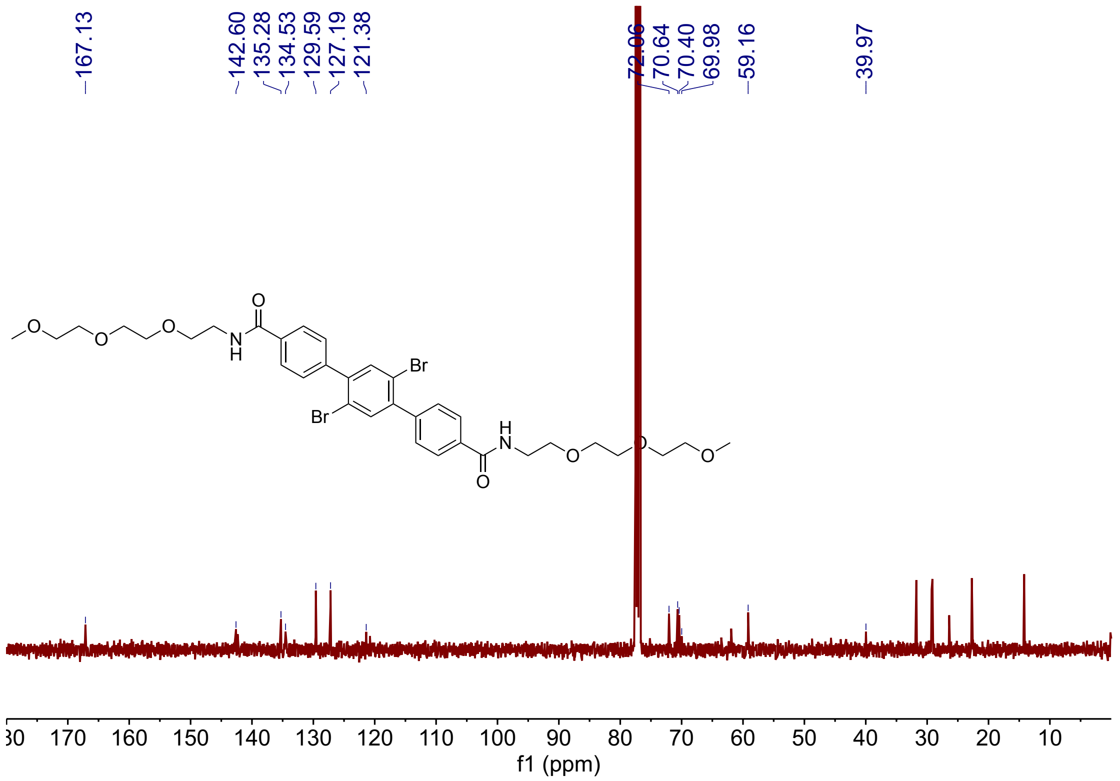

Supplementary Figure 46. ^{13}C NMR spectrum of **S3** in $CDCl_3$ (298 K).

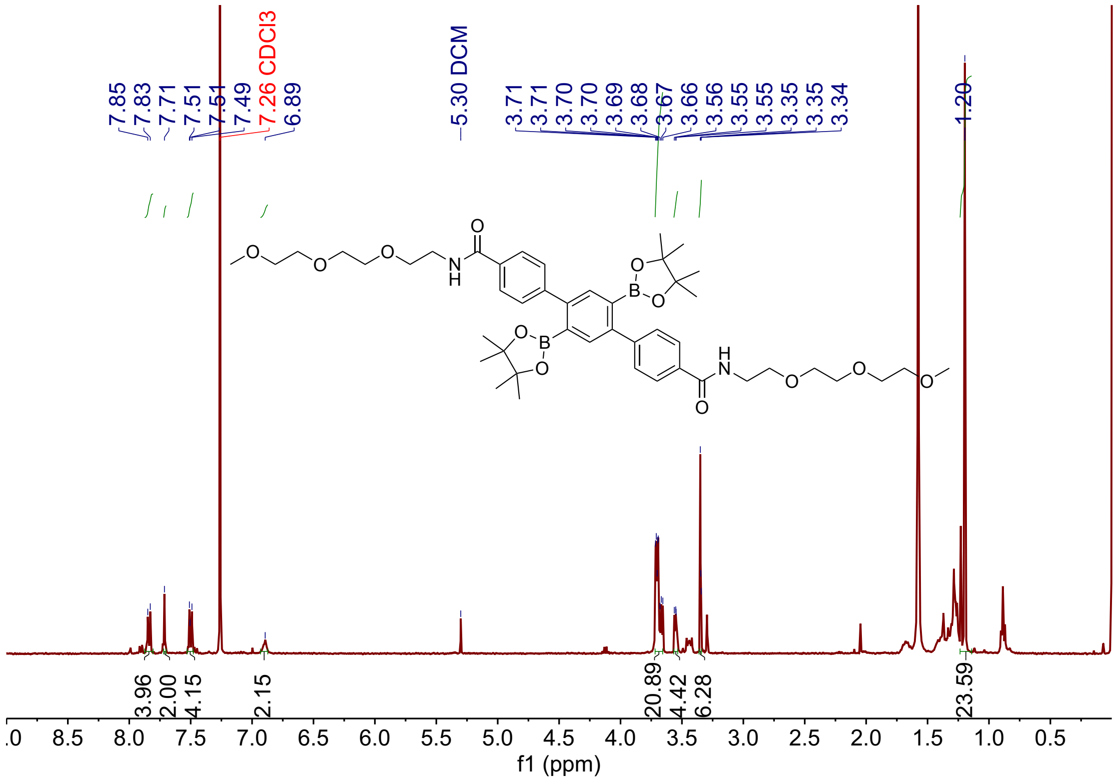

Supplementary Figure 47. ^{1}H NMR spectrum of **S4** in $CDCl_3$ (298 K).

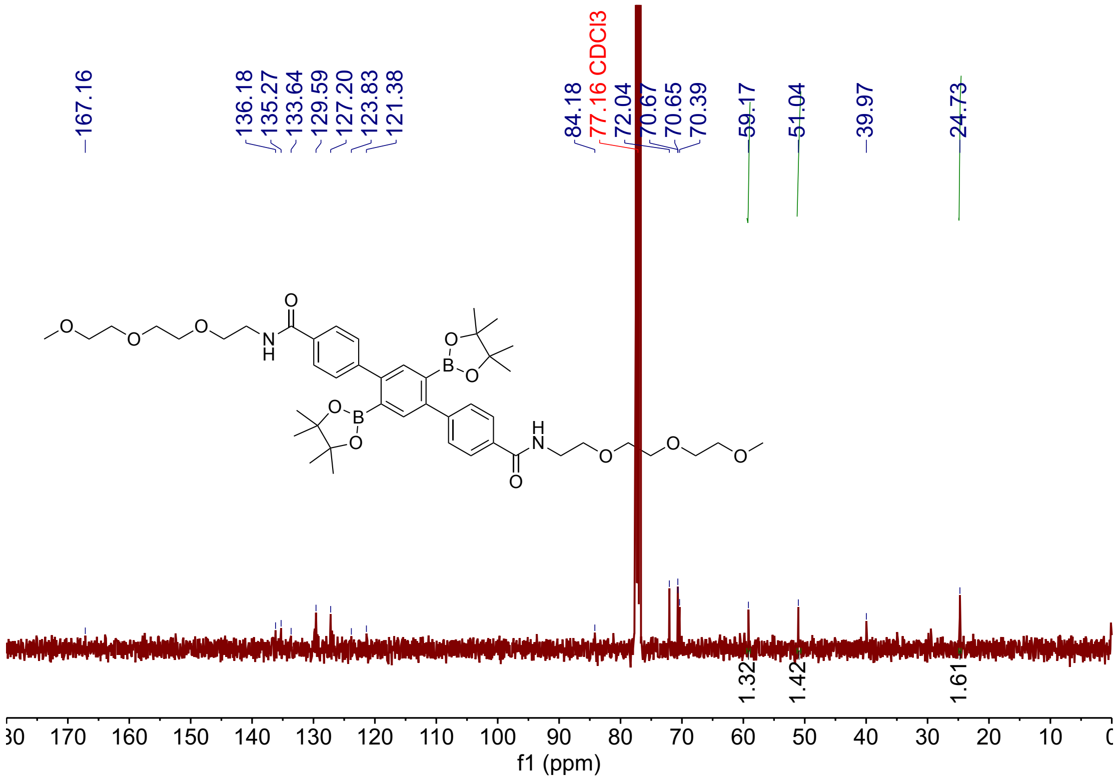

Supplementary Figure 48. ^{13}C NMR spectrum of **S4** in $CDCl_3$ (298 K).

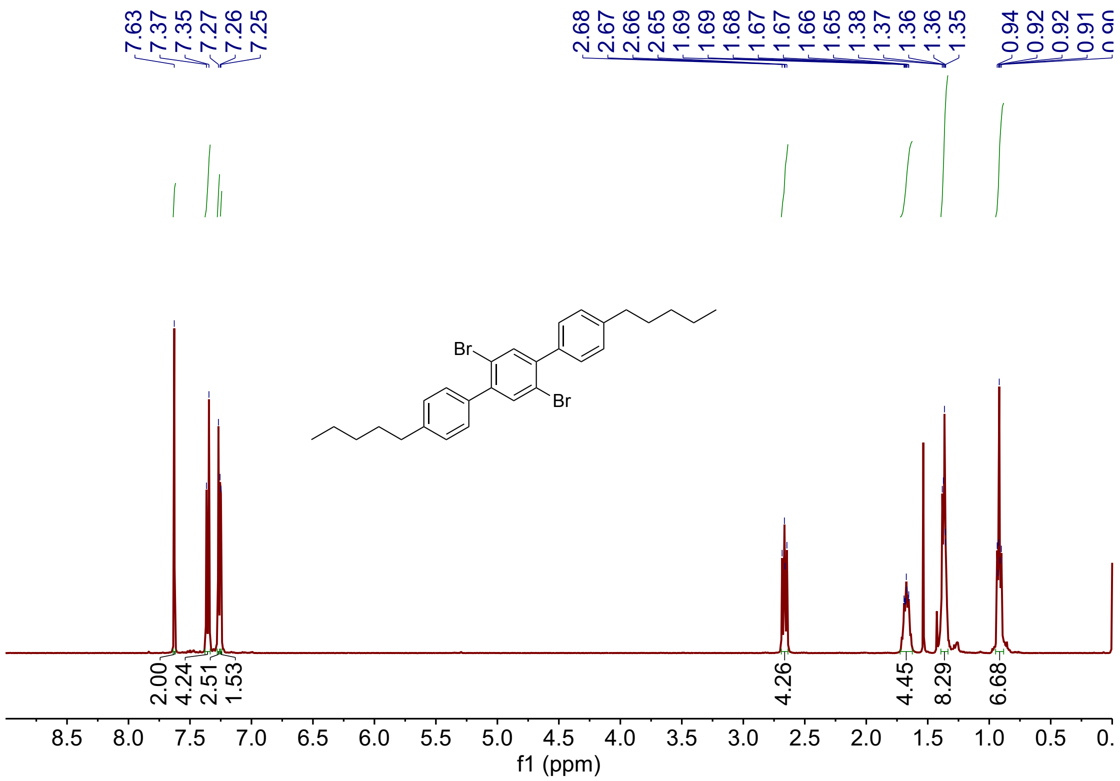

Supplementary Figure 49. ^{1}H NMR spectrum of **S6** in $CDCl_3$ (298 K).

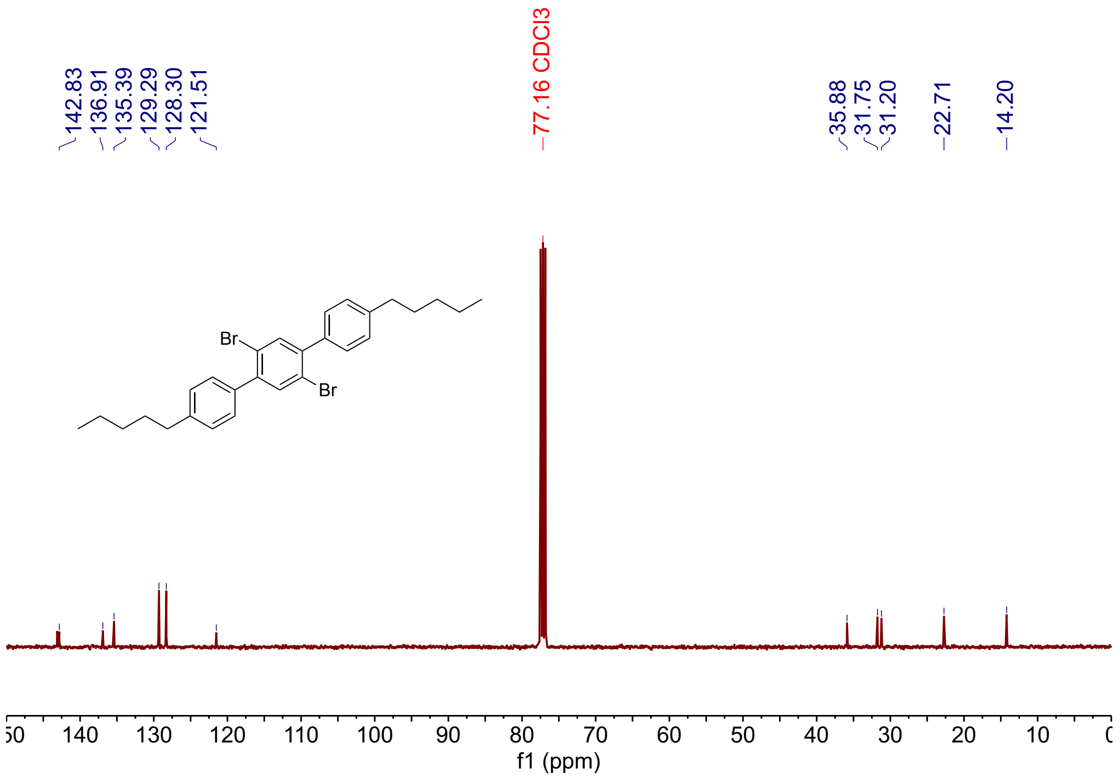

Supplementary Figure 50. ^{13}C NMR spectrum of **S6** in $CDCl_3$ (298 K).

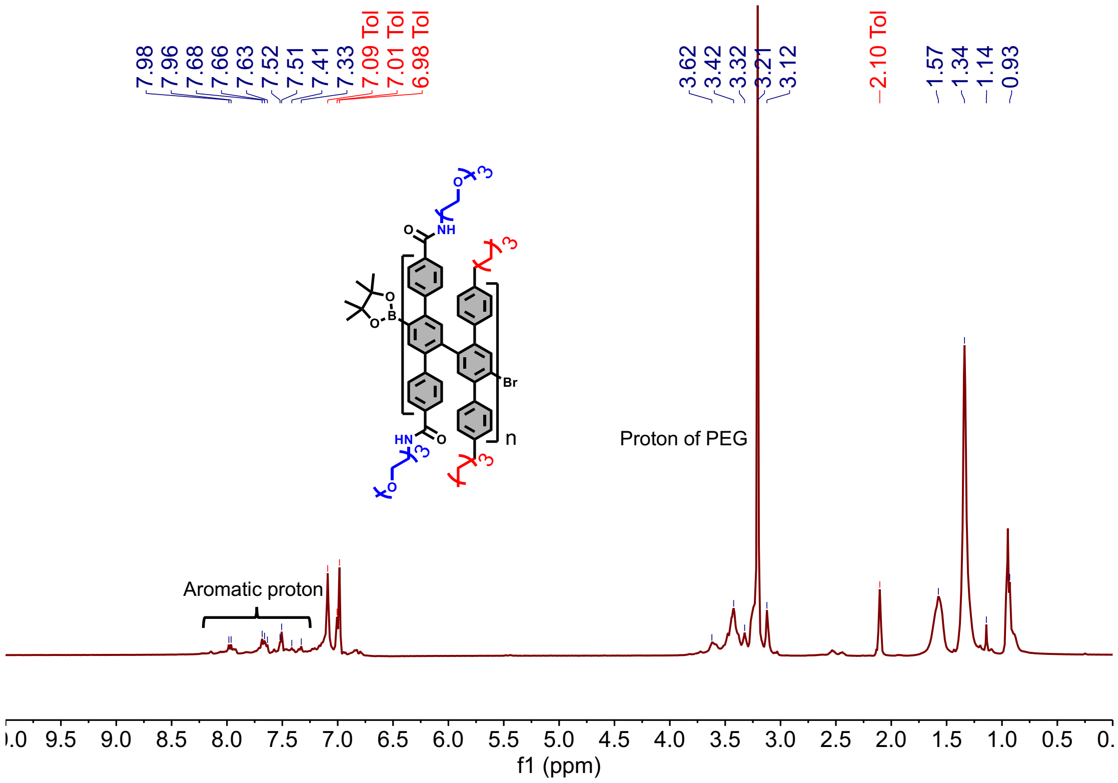

Supplementary Figure 51. ^{1}H NMR spectrum of $(AB)_n$-PT in Toluene-d_8 (378 K).

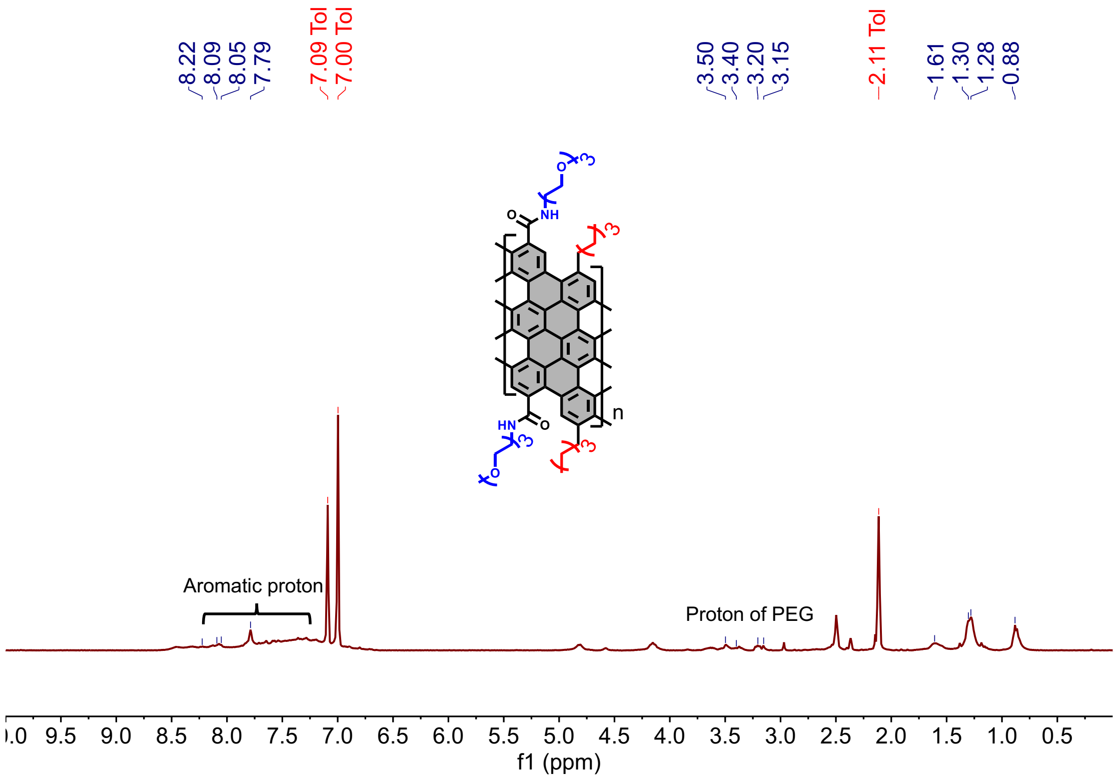

Supplementary Figure 52. ^{1}H NMR spectrum of $(AB)_n$-GNR in Toluene-d_8 (378 K).

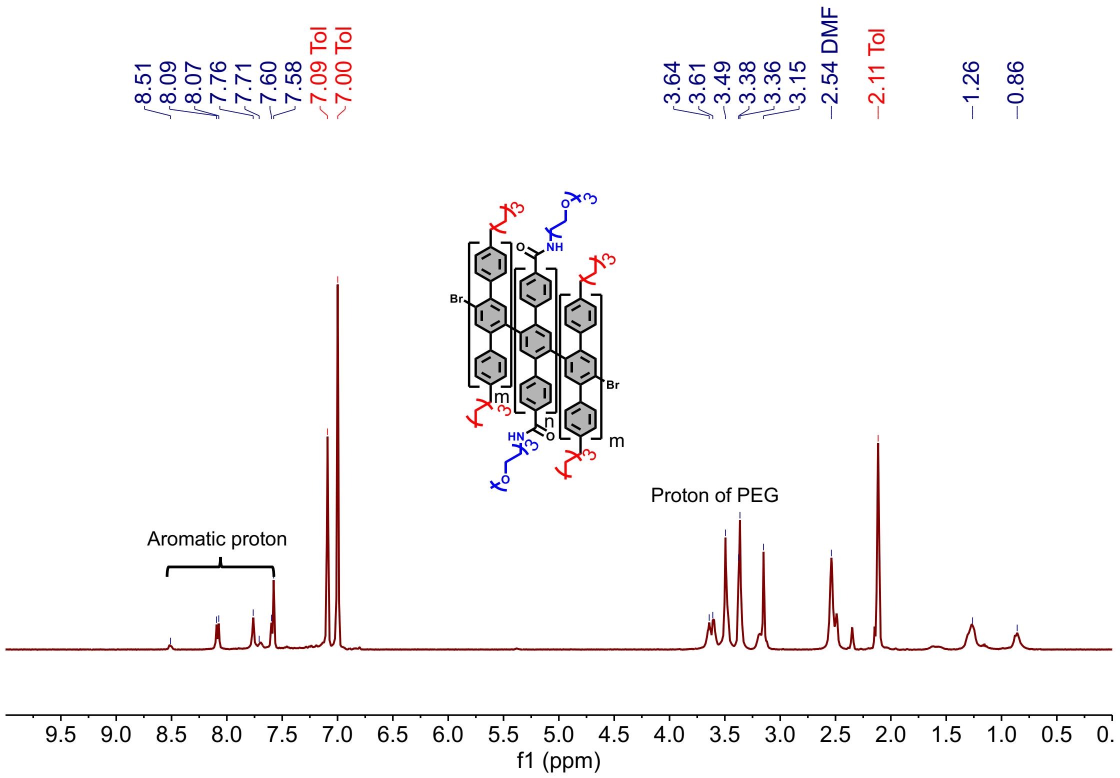

Supplementary Figure 53. ^1H NMR spectrum of $B_mA_nB_m$-PT in Toluene-d_8 (378 K).

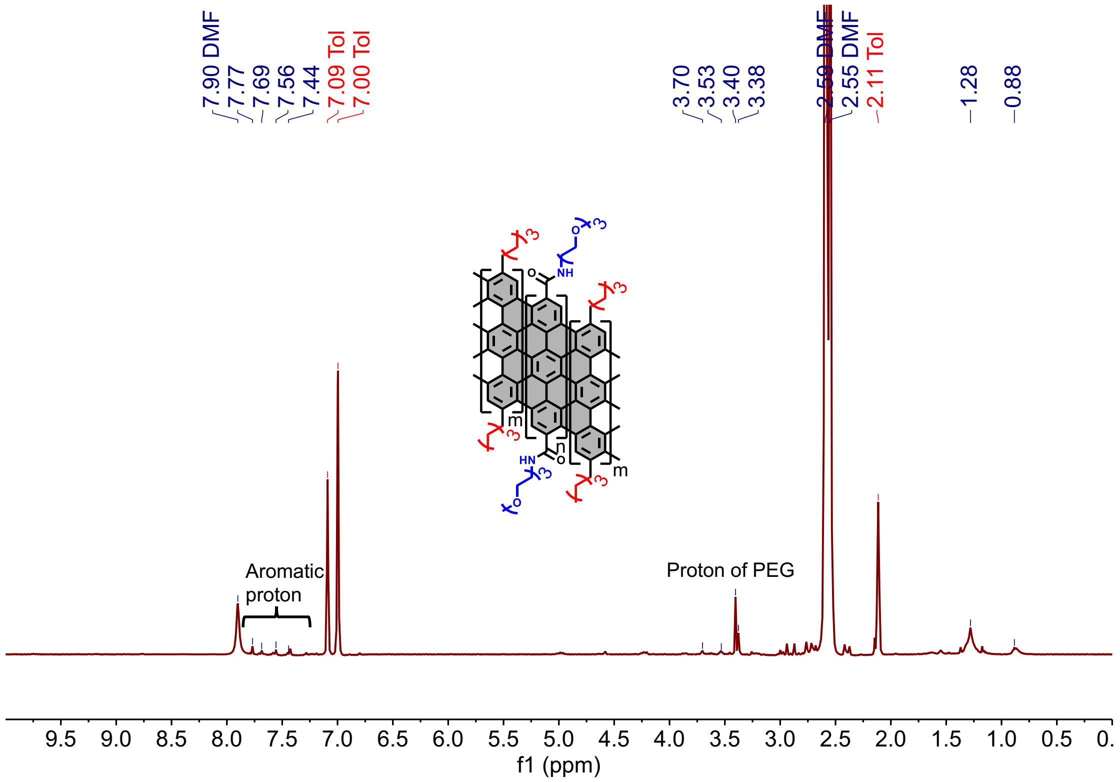

Supplementary Figure 54. ^1H NMR spectrum of $B_mA_nB_m$-GNR in Toluene-d_8 (378 K).

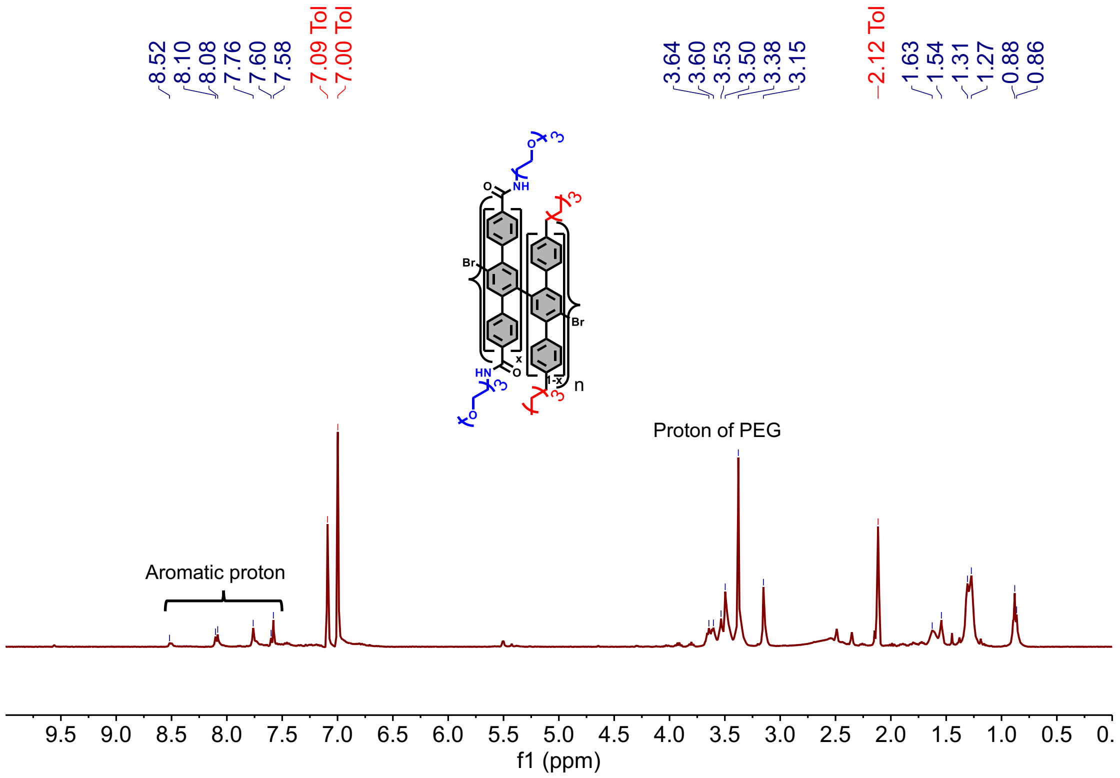

Supplementary Figure 55. ^{1}H NMR spectrum of $(A_xB_{1-x})_n$-PT in Toluene-d_8 (378 K).

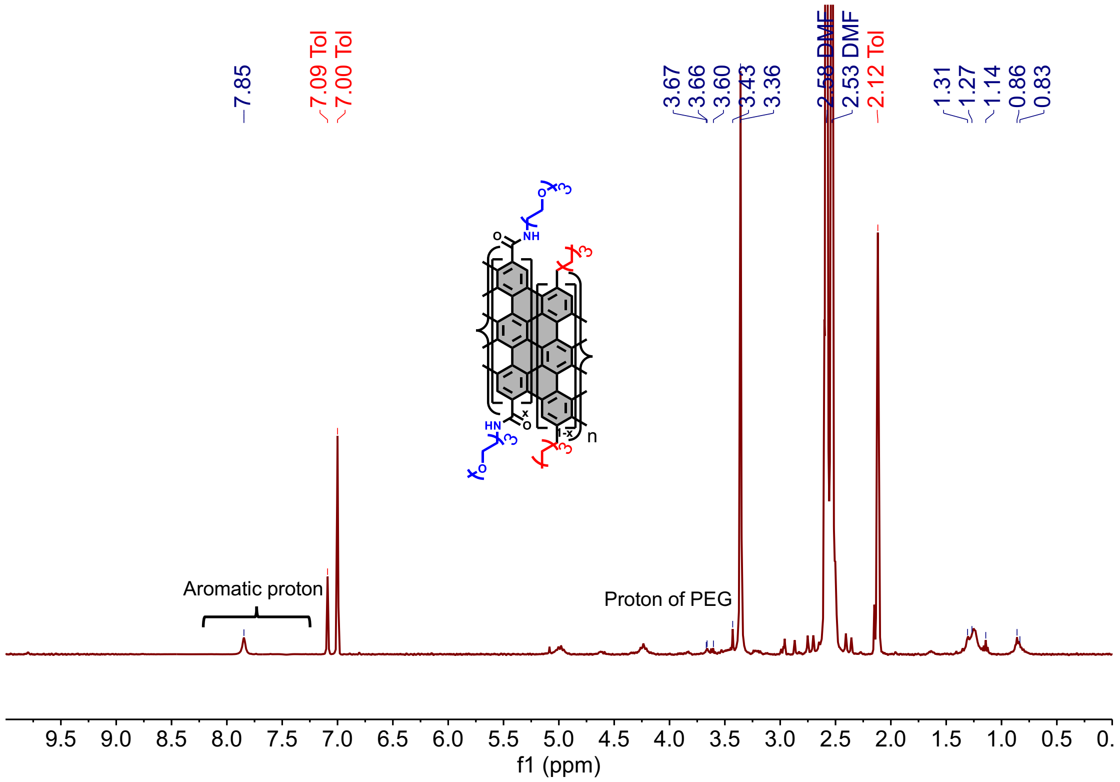

Supplementary Figure 56. ^{1}H NMR spectrum of $(A_xB_{1-x})_n$-GNR in Toluene-d_8 (378 K).

21. Supplementary mass spectra

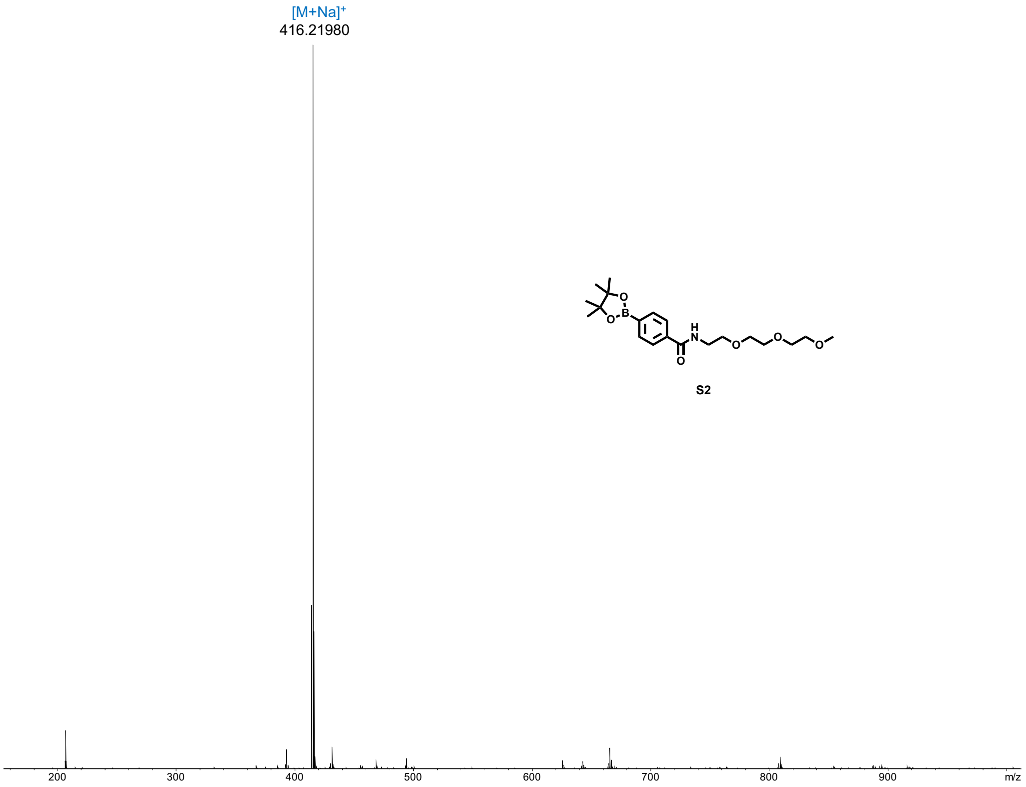

Supplementary Figure 57. MALDI-TOF-MS spectrum of **S2**.

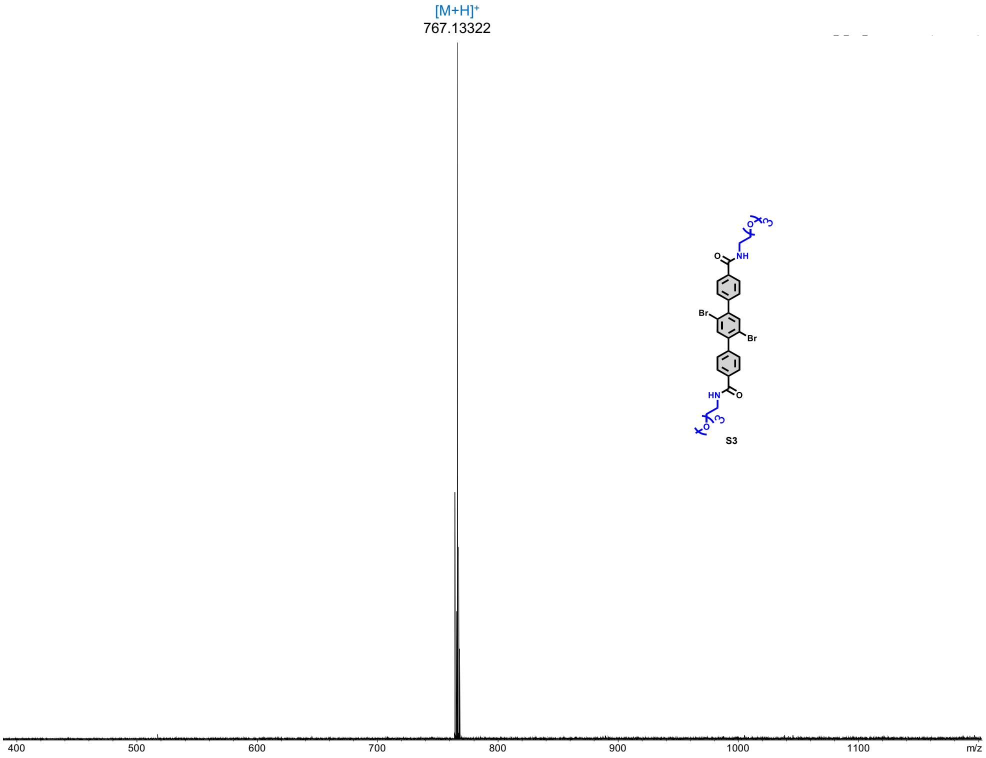

Supplementary Figure 58. MALDI-TOF-MS spectrum of **S3**.

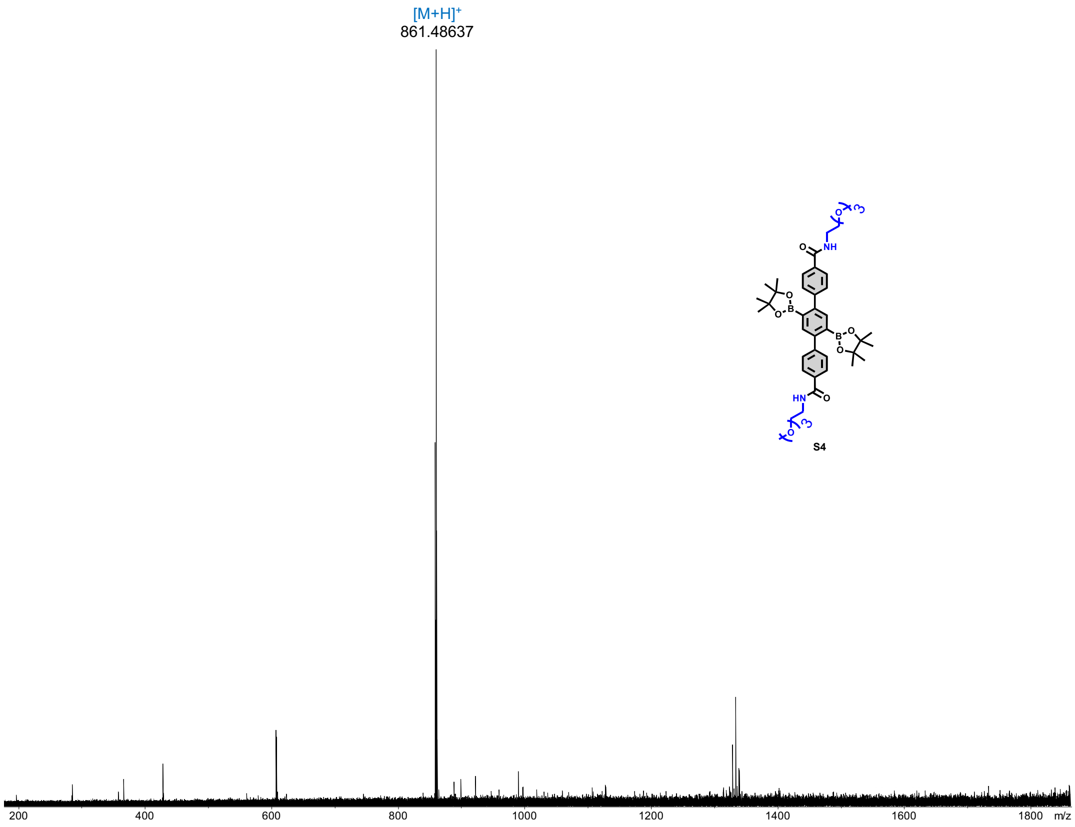

Supplementary Figure 59. MALDI-TOF-MS spectrum of **S4**.

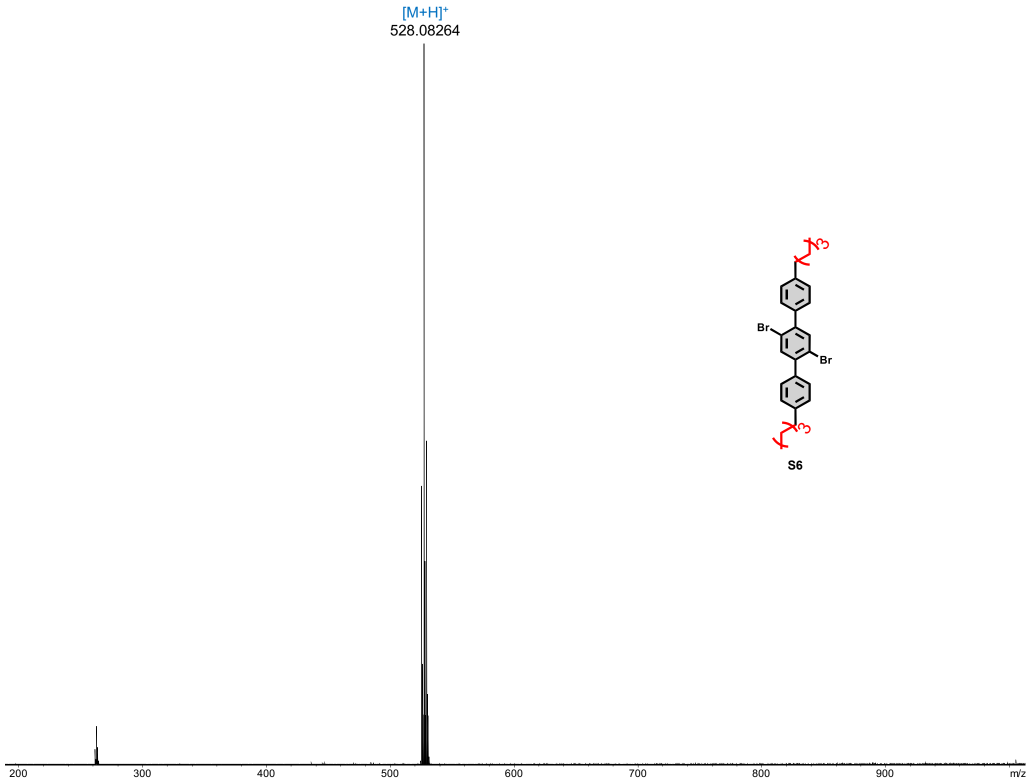

Supplementary Figure 60. MALDI-TOF-MS spectrum of **S6**.

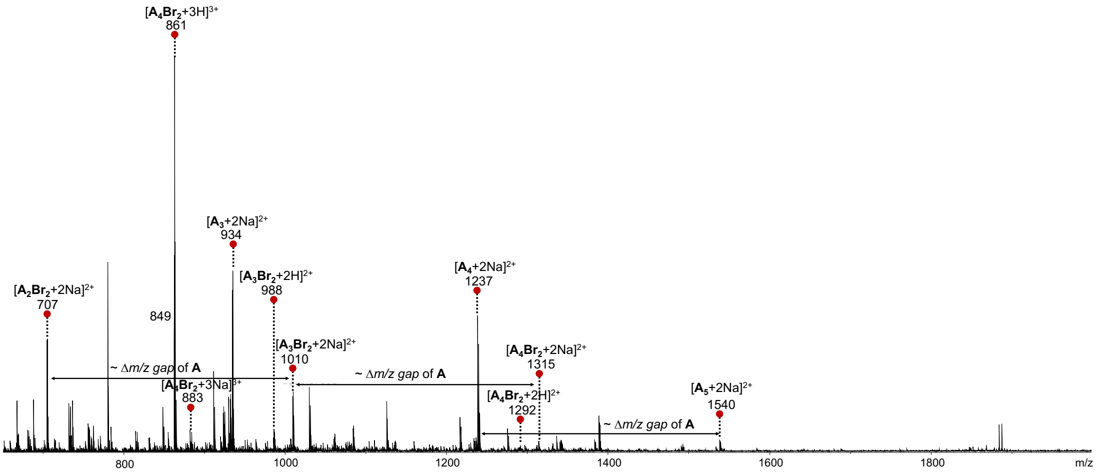

Supplementary Figure 61. ESI FT-ICR MS spectrum of A_n-PT.

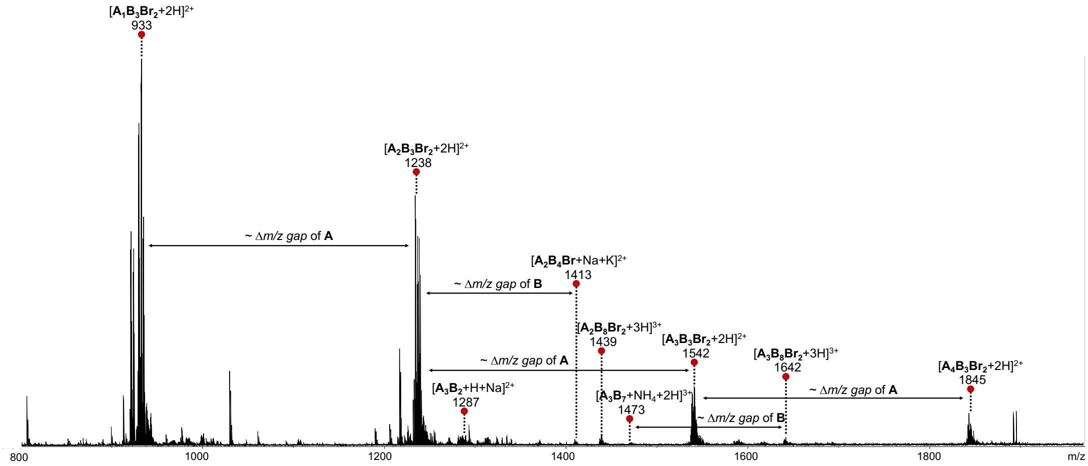

Supplementary Figure 62. ESI FT-ICR MS spectrum of $B_mA_nB_m$-PT.

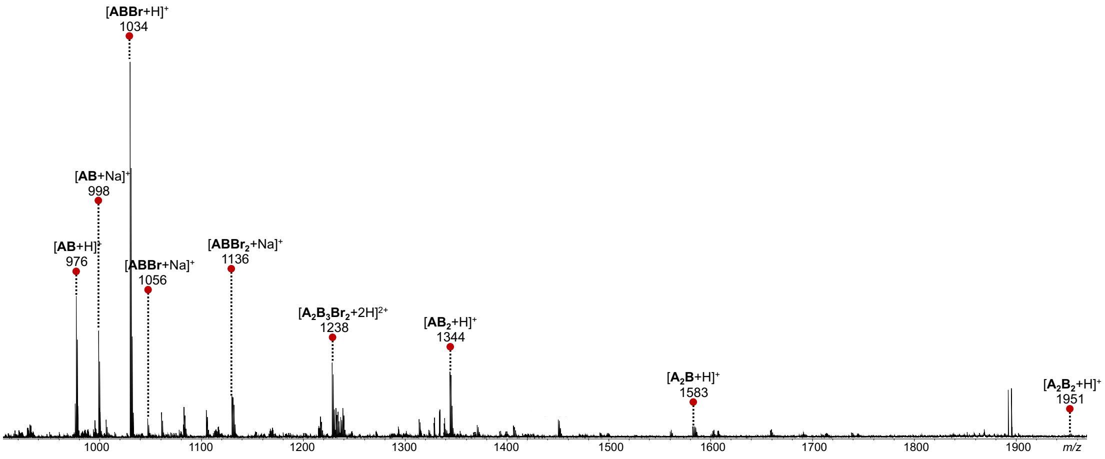

Supplementary Figure 63. ESI FT-ICR MS spectrum of $(A_xB_{1-x})_n$-PT.

22. Supplementary references

[1] Z. B. Shifrina, M. S. Averina, A. L. Rusanov, M. Wagner, K. Müllen, Branched polyphenylenes by repetitive diels−alder cycloaddition, *Macromolecules* **2000**, 33, 3525.

[2] A. Centrone, L. Brambilla, T. Renouard, L. Gherghel, C. Mathis, K. Müllen, G. Zerbi, Structure of new carbonaceous materials: the role of vibrational spectroscopy, *Carbon* **2005**, 43, 1593.

[3] I. Usov, R. Mezzenga, FiberApp: an open-source software for tracking and analyzing polymers, filaments, biomacromolecules, and fibrous objects, *Macromolecules* **2015**, 48, 1269.

[4] N. Raval, R. Maheshwari, D. Kalyane, S. R. Youngren-Ortiz, M. B. Chougule, R. K. Tekade, in *Basic Fundamentals of Drug Delivery*, (Ed: R. K. Tekade), Academic Press, 2019.

[5] Y. Huang, W. T. Dou, F. Xu, H. B. Ru, Q. Gong, D. Wu, D. Yan, H. Tian, X. P. He, Y. Mai, Supramolecular nanostructures of structurally defined graphene nanoribbons in the aqueous phase, *Angew. Chem. Int. Ed.* **2018**, 57, 3366.

[6] M. R. S. Garcia, J. D. S. Alcantara, G. V. Alea, V. D. Ebajo Jr, Non-covalent strategies for the preparation of stable aqueous graphene dispersions, *J. Chin. Chem. Soc.* **2025**, 72, 1250.

[7] N. J. Walch, A. Nabok, F. Davis, S. P. Higson, Characterisation of thin films of graphene–surfactant composites produced through a novel semi-automated method, *Beilstein J. Nanotechnol.* **2016**, 7, 209.

[8] M. J. Abraham, T. Murtola, R. Schulz, S. Páll, J. C. Smith, B. Hess, E. Lindahl, GROMACS: High performance molecular simulations through multi-level parallelism from laptops to supercomputers, *SoftwareX* **2015**, 1, 19.

[9] J. Wang, R. M. Wolf, J. W. Caldwell, P. A. Kollman, D. A. Case, Development and testing of a general amber force field, *J. Comput. Chem.* **2004**, 25, 1157.

[10] P. C. T. Souza, R. Alessandri, J. Barnoud, S. Thallmair, I. Faustino, F. Grunewald, I. Patmanidis, H. Abdizadeh, B. M. H. Bruininks, T. A. Wassenaar, P. C. Kroon, J. Melcr, V. Nieto, V. Corradi, H. M. Khan, J. Domanski, M. Javanainen, H. Martinez-Seara, N. Reuter, R. B. Best, I. Vattulainen, L. Monticelli, X. Periole, D. P. Tieleman, A. H. de Vries, S. J. Marrink, Martini 3: a general purpose force field for coarse-grained molecular dynamics, *Nat. Methods* **2021**, 18, 382.

[11] C. I. Bayly, P. Cieplak, W. Cornell, P. A. Kollman, A well-behaved electrostatic potential based method using charge restraints for deriving atomic charges: the RESP model, *J. Phys. Chem.* **1993**, 97, 10269.

[12] F. Neese, Software update: The ORCA program system-Version 5.0, *Wiley Interdisciplinary Reviews-Computational Molecular Science* **2022**, 12, e1606.

[13] T. Lu, F. Chen, Multiwfn: A multifunctional wavefunction analyzer, *J. Comput. Chem.* **2012**, 33, 580.

[14] C. Bannwarth, E. Caldeweyher, S. Ehlert, A. Hansen, P. Pracht, J. Seibert, S. Spicher, S. Grimme, Extended tight-binding quantum chemistry methods, *WIREs Computational Molecular Science* **2021**, 11, e1493.

[15] C. Bannwarth, S. Ehlert, S. Grimme, GFN2-xTB—An accurate and broadly parametrized

self-consistent tight-binding quantum chemical method with multipole electrostatics and density-dependent dispersion contributions, *J. Chem. Theory Comput.* **2019**, 15, 1652.
[16] T. Lu.
[17] P. C. Souza, R. Alessandri, J. Barnoud, S. Thallmair, I. Faustino, F. Grünewald, I. Patmanidis, H. Abdizadeh, B. M. Bruininks, T. A. Wassenaar, Martini 3: a general purpose force field for coarse-grained molecular dynamics, *Nat. Methods* **2021**, 18, 382.
[18] R. Alessandri, J. Barnoud, A. S. Gertsen, I. Patmanidis, A. H. De Vries, P. C. Souza, S. J. Marrink, Martini 3 coarse-grained force field: Small molecules, *Advanced Theory and Simulations* **2022**, 5, 2100391.
[19] R. Shrestha, R. Alessandri, M. Vögele, C. Hilpert, P. Souza, S.-J. Marrink, L. Monticelli, Martini 3 coarse-grained models for carbon nanomaterials, *ChemRxiv* **2025**.
[20] A. Barducci, G. Bussi, M. Parrinello, Well-tempered metadynamics: a smoothly converging and tunable free-energy method, *Phys. Rev. Lett.* **2008**, 100, 020603.
[21] G. Bussi, D. Donadio, M. Parrinello, Canonical sampling through velocity rescaling, *J. Chem. Phys.* **2007**, 126.
[22] M. Bernetti, G. Bussi, Pressure control using stochastic cell rescaling, *J. Chem. Phys.* **2020**, 153.
[23] M. Invernizzi, M. Parrinello, Rethinking metadynamics: from bias potentials to probability distributions, *J. Phys. Chem. Lett.* **2020**, 11, 2731.
[24] M. Invernizzi, M. Parrinello, Exploration vs convergence speed in adaptive-bias enhanced sampling, *J. Chem. Theory Comput.* **2022**, 18, 3988.
